# Material basis and molecular mechanisms of Chaihuang Qingyi Huoxue Granule in the treatment of acute pancreatitis based on network pharmacology and molecular docking-based strategy

**DOI:** 10.3389/fimmu.2024.1353695

**Published:** 2024-05-03

**Authors:** Jia Yang, Yu-Hong Jiang, Xin Zhou, Jia-Qi Yao, Yang-Yang Wang, Jian-Qin Liu, Peng-Cheng Zhang, Wen-Fu Tang, Zhi Li

**Affiliations:** ^1^ School of Integrated Traditional Chinese and Western Medicine, Southwest Medical University, Luzhou, Sichuan, China; ^2^ Department of Integrated Traditional Chinese and Western Medicine, National Clinical Research Center for Geriatrics, West China Hospital, Sichuan University, Chengdu, China; ^3^ Department of Spleen and Stomach Diseases, Chinese Medicine Hospital Affiliated to Southwest Medical University, Luzhou, Sichuan, China; ^4^ The Key Laboratory of Integrated Traditional Chinese and Western Medicine for Prevention and Treatment of Digestive System Diseases of Luzhou city, Affiliated Traditional Medicine Hospital of Southwest Medical University, Luzhou, China

**Keywords:** acute pancreatitis, Chaihuang Qingyi Huoxue Granule, network pharmacology, molecular docking, pancreatic acinar cells, Traditional Chinese

## Abstract

**Objectives:**

This study aimed to analyze active compounds and signaling pathways of CH applying network pharmacology methods, and to additionally verify the molecular mechanism of CH in treating AP.

**Materials and methods:**

Network pharmacology and molecular docking were firstly used to identify the active components of CH and its potential targets in the treatment of AP. The pancreaticobiliary duct was retrogradely injected with sodium taurocholate (3.5%) to create an acute pancreatitis (AP) model in rats. Histological examination, enzyme-linked immunosorbent assay, Western blot and TUNEL staining were used to determine the pathway and mechanism of action of CH in AP.

**Results:**

Network pharmacological analysis identified 168 active compounds and 276 target proteins. In addition, there were 2060 targets associated with AP, and CH had 177 targets in common with AP. These shared targets, including STAT3, IL6, MYC, CDKN1A, AKT1, MAPK1, MAPK3, MAPK14, HSP90AA1, HIF1A, ESR1, TP53, FOS, and RELA, were recognized as core targets. Furthermore, we filtered out 5252 entries from the Gene Ontology(GO) and 186 signaling pathways from the Kyoto Encyclopedia of Genes and Genomes(KEGG). Enrichment and network analyses of protein-protein interactions predicted that CH significantly affected the PI3K/AKT signaling pathway, which played a critical role in programmed cell death. The core components and key targets showed strong binding activity based on molecular docking results. Subsequently, experimental validation demonstrated that CH inhibited the phosphorylation of PI3K and AKT in pancreatic tissues, promoted the apoptosis of pancreatic acinar cells, and further alleviated inflammation and histopathological damage to the pancreas in AP rats.

**Conclusion:**

Apoptosis of pancreatic acinar cells can be enhanced and the inflammatory response can be reduced through the modulation of the PI3K/AKT signaling pathway, resulting in the amelioration of pancreatic disease.

## 1 Introduction

Acute pancreatitis (AP) is a digestive disorder that has a widespread occurrence and can be extremely serious, even leading to death. The condition is distinguished by abrupt abdominal ache and increased levels of pancreatic enzymes in the blood (1). Despite the progress made in comprehending the development of AP and enhancing its medical treatment, the occurrence and fatality rates of AP persistently rise (2, 3). Currently, Western medical treatments for AP mainly focus on acid suppression, inhibition of pancreatic enzyme secretion, antispasmodics, and pain relief. Further studies are required to explore multiple methods of treating AP. Several research studies have shown the effectiveness of traditional Chinese medicine (TCM) for treating AP. TCM is a cost-effective option with minimal side effects (4, 5), deserving further study.

The Department of Gastroenterology at the Affiliated Traditional Chinese Medicine Hospital of Southwest Medical University utilizes an in-house remedy called Chaihuang Qingyi Huoxue Granule (CH) to effectively treat cases of severe acute pancreatitis (6). It contains 14 Chinese herbs, Chaihu (Bupleurum), Houpu (Magnolia Bark), Chishao (Red Peony Root), Dahuang (Rhubarb), Taoren (Peach Kernel), Danshen (Salvia miltiorrhiza), Gancao (Licorice Root), Yanhusuo (Corydalis Yanhusuo), Huangqi (Astragalus Root), Huangqin (Scutellaria Baicalensis Root), Zhishi (Immature Bitter Orange), Zhizi (Gardenia Fruit), Baishao (White Peony Root), and Pugongying (Dandelion). For numerous years, CH has been utilized in clinical settings to treat AP. Evidence (7, 8) has shown that CH can regulate gastrointestinal motility and inhibit the production and release of pro-inflammatory factors, thereby alleviating AP. However, the precise chemical composition and exact mechanisms of action have yet to be fully elucidated.

**Figure 1:**
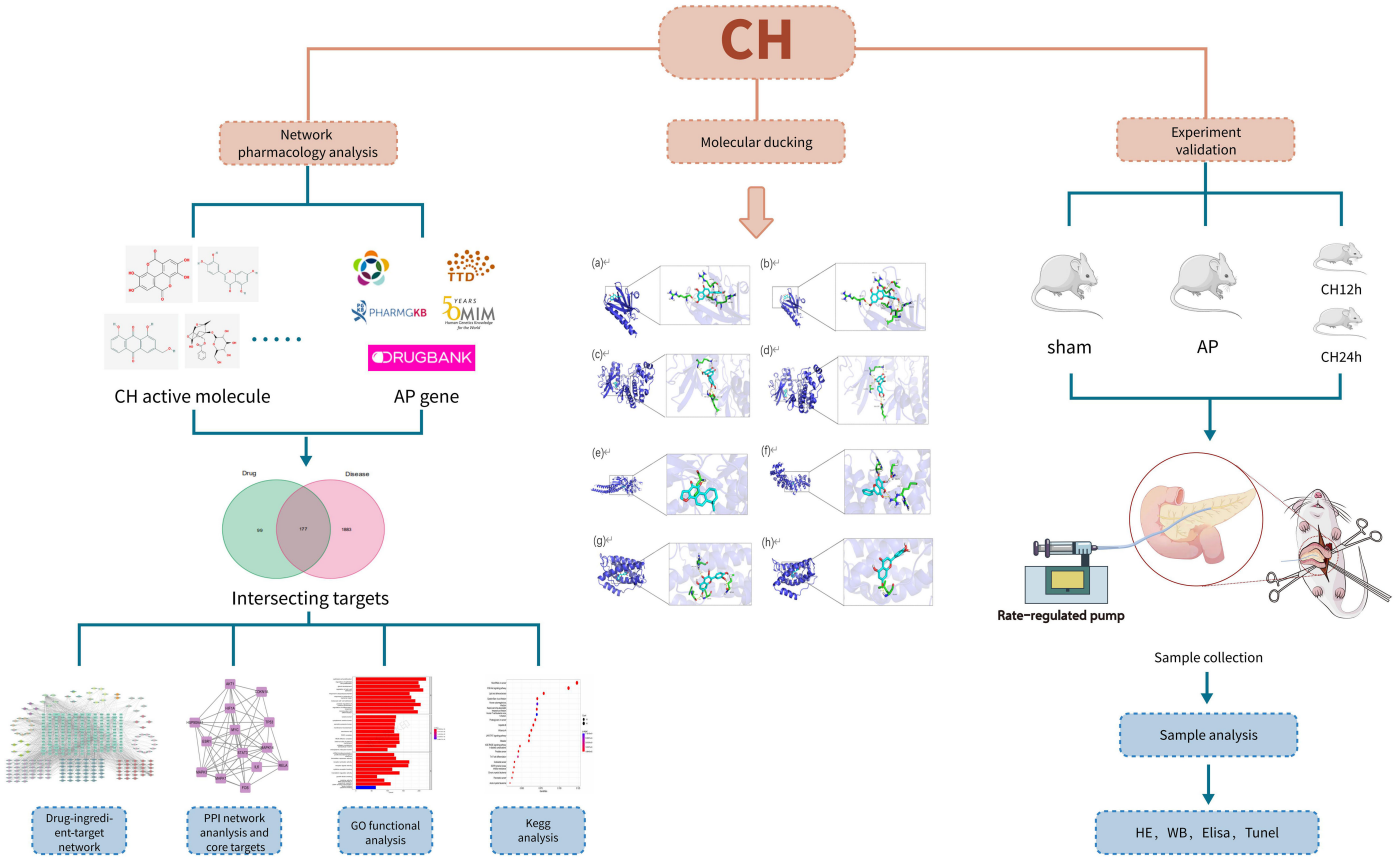
All technical approaches rely on network pharmacology and experimental verification.

Network pharmacology and molecular docking were effective methods that utilize advanced computer simulations to identify components and predict drug targets. In this study, the active ingredients of CH were identified through data mining, targeting identification, and the establishment of a comprehensive network. Additionally, enrichment analysis using GO and KEGG revealed the involvement of the PI3K/Akt signaling pathway, thereby predicting the potential mechanism of CH in treating AP. Subsequently, a series of animal experiments, including HE staining, WB, ELISA, TUNEL, etc., were conducted to validate that CH could inhibit the activation of the PI3K/Akt signaling pathway, induce apoptosis of pancreatic acinar cells, reduce inflammatory reactions, and mitigate damage in AP model rats. This suggests that network pharmacology can be an effective approach to elucidate the mechanism of action of CH in treating AP. The research route is illustrated in **Figure 1**.

## 2 Materials and methods

### 2.1 Network pharmacology predictions

#### 2.1.1 Data mining for chemical ingredients

Information regarding the specific plants in CH was extracted from the Traditional Chinese Medicine System Pharmacology Database (TCMSP) (9). The molecular structures of every compound were obtained from the NCBI PubChem database (10) and confirmed through thorough research in literature. It is crucial to evaluate the absorption, distribution, metabolism, and excretion characteristics of every compound in order to identify the active components of CH and examine their functional foundation. For this investigation, we opted for oral absorption (OA) and drug similarity (DS). For the purpose of further analysis, molecules with OB ≥ 30% were chosen in this particular section (11). In TCM research, the DL threshold of 0.18, which is based on the average value of all compounds in the DrugBank database (12), is widely used as a screening standard for identifying ‘drug-like’ compounds.

#### 2.1.2 Compounds target fishing

In this process, the active ingredients filtered out early were used as bait to identify the corresponding targets in the TCMSP and DrugBank databases. Afterwards, the UniProt KB (13) was used to map all stationary objectives and acquire their official gene symbols.

#### 2.1.3 Disease targets database building

The Gene Cards database (14), OMIM database (15), PharmGkb database (16), TTD (17, 18), and DrugBank database were used to screen AP-related targets. We combined the targets from these five databases, removed duplicate targets, and identified the disease targets related to AP. In this section, the search was conducted using the keywords “acute pancreatitis” and selection of species condition for “homo sapiens” as a supplement. Subsequently, the retrieved targets were mapped to UniProt KB to obtain the corresponding official gene symbols.

In the end, we compared the projected goals of CH active components with the targets related to AP and chose the common targets as potential therapeutic targets for CH. A Venn diagram of the targets was plotted and visualized using FunRich software in the CH-AP network. Subsequently, the common targets were processed for additional analysis.

#### 2.1.4 Enrichment analysis of gene ontology and KEGG pathway

In order to delve deeper into the possible molecular mechanism of CH, we conducted GO enrichment analysis and KEGG signaling pathway analysis. This involved connecting the chosen possible targets to the Database Visualization and Integrated Discovery system (DAVID) Bioinformatics Resources 6.8 (19). Only the terms with *P* < 0.05 significance level were taken into account in this particular section. The overlapping targets were plotted using R language, and the GO results were represented using a bar chart, while the KEGG analysis results were represented using a bubble chart.

#### 2.1.5 Network construction and analysis

Drug-compound-target (D-C-T) network of CH was created by establishing connections between active compounds and their potential targets using Cytoscape v3.9.1 software (20), a robust tool frequently employed in bioinformatics research for data integration and visualization. Nodes in graphical networks depict compounds and targets, while edges represent depict interactions between the compound and target. Moreover, ‘degree’, a crucial topological parameter in network pharmacology, was examined using the CytoNCA plugin of Cytoscape. The degree of a node indicates the total number of other nodes that interact with it.

Construction of a network for the interaction between proteins (PPI) was carried out to identify potential therapeutic targets of the active components in CH. The STRING database (21) was utilized for this purpose. The settings for network analysis consisted of choosing the mode of analysis as ‘Multiple proteins’, selecting ‘Homo sapiens’ as the species, and setting a confidence score of at least 0.95. Isolated proteins were excluded, and the results were saved as a TSV file. Afterwards, we employed the Cytoscape 3.9.1 application to exhibit the protein-protein interaction (PPI) network. To pinpoint the main targets, we carried out network topology analysis through the “Network Analyzer” module in Cytoscape. Different parameters, including Betweenness (BC), Closeness (CC), Degree (DC), Eigenvector (EC), local average connectivity-based methods (LAC), Network (NC), and Information (IC), were used to establish the main objectives. During this process, the core targets were identified by assessing the degree of each node in the network.

After conducting the KEGG pathway analysis on the potential targets identified in the DAVID database, the Compound-Target-Pathway (C-T-P) network was built in Cytoscape 3.9.1. This network connected the top 30 KEGG signaling pathways with their respective targets. Nodes in graphical networks represent compounds, targets, and signaling pathways, while edges represent C-T-P interactions. A node’s significance in the network is indicated by a higher degree value.

#### 2.1.6 Molecular docking verification of core components and targets

The network known as the ‘Compound-Target-Pathway (C-T-P)’ was employed to discover compounds and their possible targets. These targets were subsequently analyzed through molecular docking investigations with AutoDock Vina (22). Molecular docking involves several steps. Initially, the PDB database (23) was utilized to acquire the three-dimensional arrangement of the main protein of interest. Second, this structure was imported into PyMOL software to eliminate water molecules and inactive ligands. Following this, the structure was loaded into AutoDockTools software for hydrogenation and charging, and finally saved in PDB format. Third, the target proteins and the components of CH were transformed into the pdbqt file format, which is in line with AutoDock Vina’s compatibility. To facilitate the docking of the target protein’s active site, the size and coordinates of the Grid Box were adjusted. Subsequently, the active components of CH were docked onto the active site of the target protein. Finally, the components that had the best binding energy and strongest affinity towards the target proteins were selected. Using the PyMOL software, we visualized the docked complexes of the selected elements.

### 2.2 Experiment validation

#### 2.2.1 CH preparation

Each bag of CH contains 7 g. The recommended dosage for adults with AP is 42 g, three times per day. In the case of rats, the equivalent dosage was 6.3 times that in adults. Therefore, the dosage of CH in the rats was approximately 0.7 g/kg.BW × 6.3 ≈ 4.4 g/kg.BW = 0.44 g/100g.BW. CH was dissolved in saline at a 0.44 g/mL concentration. The rats were gavaged with 1 mL of the solution per 100 g of body weight.

#### 2.2.2 Reagents

Sodium taurocholate (Item No. The purity of 97-98% was acquired from Beijing Bioway Technology Co., Ltd. The CH ready-to-use granules were obtained from the Affiliated Traditional Chinese Medicine Hospital of Southwest Medical University. Ruixin Biotech was the source of the purchased ELISA kits (RX302856R, RX302869R, and RX302058R). An apoptosis detection kit (T-6013) was purchased from UElandy (China). Antibodies against p-PI3K (ab182651) were purchased from Abcam (Cambridge, USA). The β-actin antibody (AT0040) was purchased from Engibody (USA). Proteintech (USA) provided the antibodies for phosphoinositide 3-kinase (PI3K) (60225-1-Ig), Bcl-2 (68103-1-Ig), P-P65 (82335-1-RR), and P65 (10745-1-AP). Cell Signaling Technology (Danvers, MA, USA) was the source of the purchased antibodies targeting Bax (14796S), AKT (8200S), and p-Akt (8200S). All additional substances utilized in this research were of analytical quality and obtained from nearby vendors.

#### 2.2.3 Animal experiment

##### 2.2.3.1 Animals

A total of sixty male Sprague-Dawley rats, averaging 200 ± 20 g in weight, were acquired from the Animal Ethics Committee at Southwest Medical University. The animals were housed in a pathogen-free facility, where the humidity levels were kept between 40% and 70%, the temperature was sustained at 22 ± 2°C, and a 12-hour light-dark cycle was adhered to. The rats were given standard rodent feed and had unrestricted access to water during the experiment. The Animal Ethics Committee of the Southwest Medical University (NO.20221222-002) granted approval for all animal experiments. All rats were subjected to a 12h fasting period in both the pre-modeling and post-modeling phases.

##### 2.2.3.2 Experimental design and induction of AP

The animals were allocated at random to two primary groups: a 12h group (n=30) and a 24h group (n=30). Each primary group was subsequently divided into three subgroups: sham (n=10), AP (n=10), and CH (n=10). AP was induced in the AP model group through the injection of 3.5% sodium taurocholate into the pancreaticobiliary duct in a retrograde manner (24). The control group received an identical surgical intervention, with the exception that they were given 0.9% saline instead. In the CH group, CH (1 mL/100 g.BW) was administered by gavage 6h after modeling. Samples of blood and pancreatic tissues were obtained at 12 and 24 hours following administration via gavage.

### 2.3 Experiment assay

#### 2.3.1 Pancreatic histopathology and scoring

The pancreas tissue was preserved in a 4% solution of paraformaldehyde, underwent dehydration using a gradient method, was embedded in paraffin wax. Hematoxylin and eosin (HE) staining was performed on thick sections sliced to a 5 μm thickness. Pancreatic abnormalities following HE staining were evaluated by two pathologists using the Schmidt pathology scoring criteria (25) under a light microscope, employing a double-blind approach. Five randomly selected fields were assessed to determine the scores for edema, inflammation, and necrosis. Ultimately, the mean score was used to quantify the extent of damage.

#### 2.3.2 TdT-mediated dUTP Nick-end labeling assay

The paraffin sections were deparaffinized in water. Proteinase K was used to permeabilize the tissues. The TUNEL reaction solution was applied to tissues and incubated in the dark. Finally, the sections were sealed after DAPI counterstaining. Fluorescence microscope was utilized to capture images and ImageJ software was employed for quantification.

#### 2.3.3 Western blot

Protein specimens were obtained by utilizing RIPA lysis solution comprising of protease and phosphatase inhibitors, and protein concentration was ascertained utilizing a BCA assay kit. Following the electrophoretic separation of proteins, PVDF membranes were utilized to facilitate their transfer. Next, the antibodies were encapsulated with 5% BSA for a duration of 30 minutes. Subsequently, the membrane was incubated with the specified antibodies at 4°C overnight. After undergoing three 10-minute washes with TBST, the membrane underwent incubation with a secondary antibody conjugated with horseradish peroxidase for a duration of 2 hours. Subsequently, the membrane was washed again and subjected to incubation with an ECL chemiluminescent substrate to facilitate imaging. The obtained images were analyzed using the ImageJ software.

#### 2.3.4 The enzyme-linked immunosorbent assay and blood biochemistry

ELISA was used to detect the serum levels of interleukin (IL)-6, IL-1β, and tumor necrosis factor-alpha (TNF-α) in accordance with the manufacturer’s guidelines. Additionally, an automated biochemical analyzer was utilized to measure the levels of serum amylase and lipase.

### 2.4 Statistics

We calculated the mean ± standard deviation using the statistical tool GraphPad Prism 9.5. The variance was calculated to satisfy the normal distribution of the data, and one-way ANOVA (One-Way ANOVA) was chosen when the variance was uniform; the Kruskal-Wallis H test was used to do a nonparametric test for multiple independent samples when none of the conditions were met. The statistical significance level was established to be below 0.05 and identified as *P* < 0.05.

## 3 Results

### 3.1 Network pharmacology prediction analysis

#### 3.1.1 Active compounds and targets in CH

By screening the TCMSP database, a total of 168 active components and 276 targets were identified. The results indicate that most of the compounds interact with multiple targets (**Supplementary Table S1**). The active components of Baishao, Chaihu, Chishao, Dahuang, Danshen, Gancao, Houpu, Huangqin, Huangqi, Taoren, Yanhusuo, Zhishi, and Zhizi were 3, 7, 8, 5, 28, 40, 2, 13, 7, 16, 42, 4, and 7, respectively. The 10 molecules with the highest OB scores are listed in **Table 1**.

**Table 1:** Representative molecules from CH and their corresponding OB, DL, and structures.

Drug	MolId	MolName	MW	OB (%)	DL	structure
Danshen	MOL007064	przewalskin b	330.46	110.32	0.44	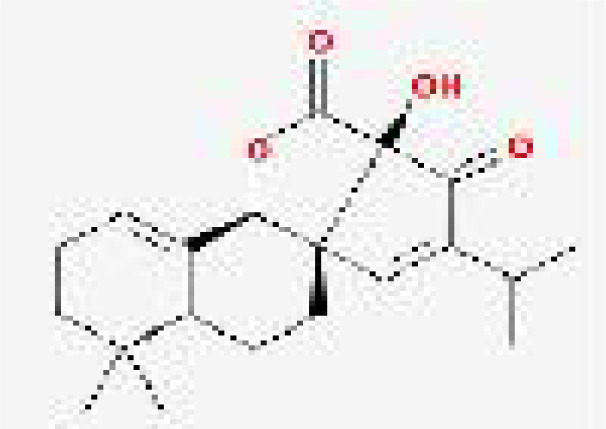
Huangqin	MOL002934	NEOBAICALEIN	374.37	104.34	0.44	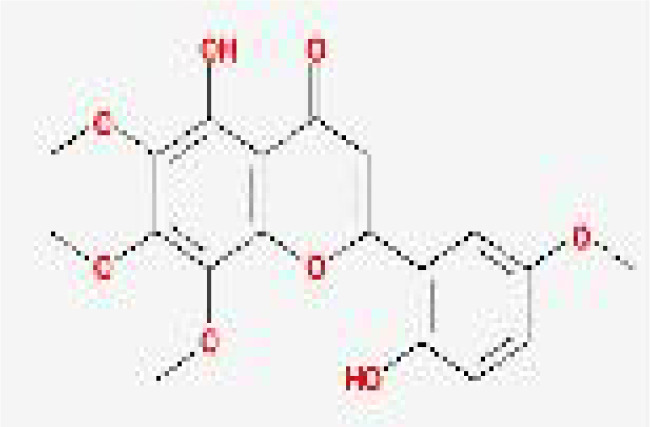
Taoren	MOL001351	Gibberellin A44	346.46	101.61	0.54	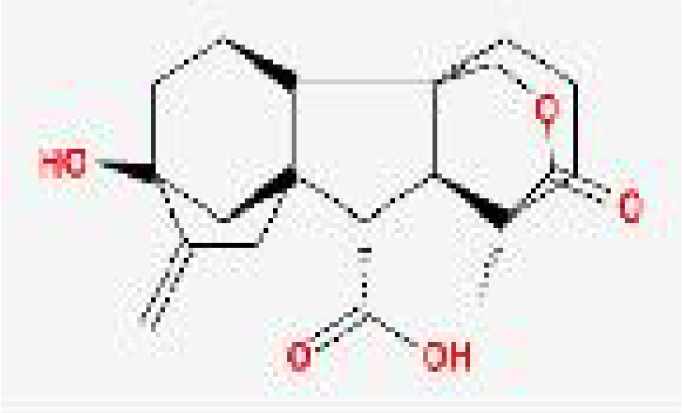
Taoren	MOL001353	GA60	348.43	93.17	0.53	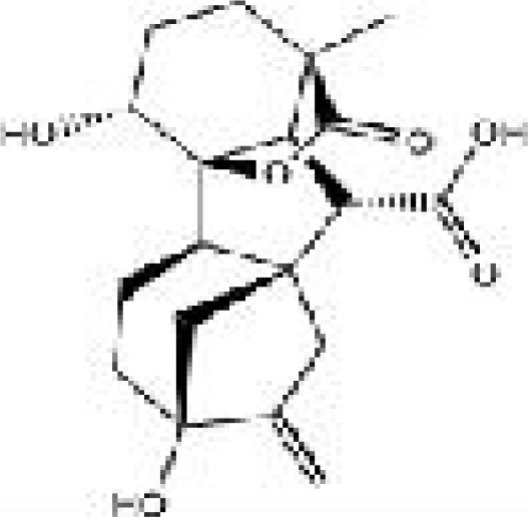
Gancao	MOL002311	Glycyrol	366.39	90.78	0.67	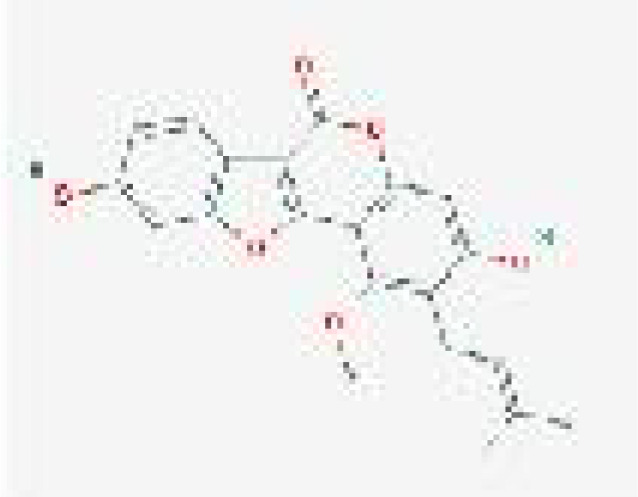
Taoren	MOL001349	4a–formyl–7alpha–hydroxy–1–methyl–8–methylidene–4aalpha,4bbeta–gibbane–1alpha,10beta–dicarboxylic acid	362.46	88.6	0.46	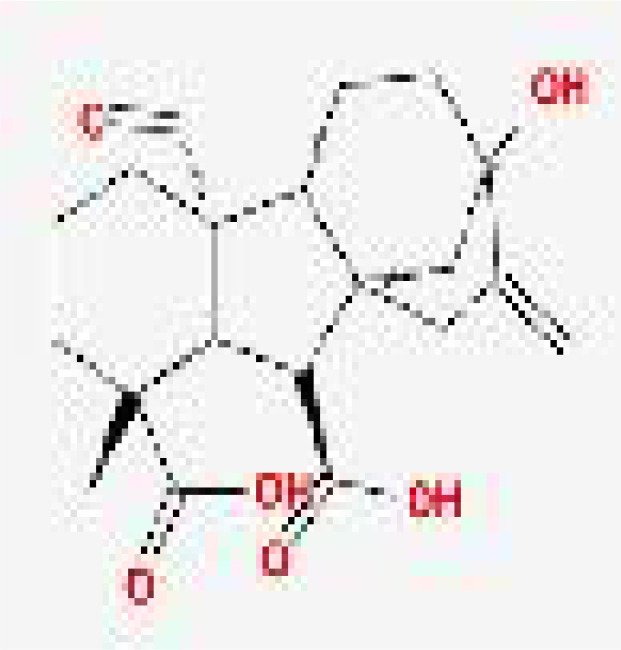
Taoren	MOL001344	GA122–isolactone	330.41	88.11	0.54	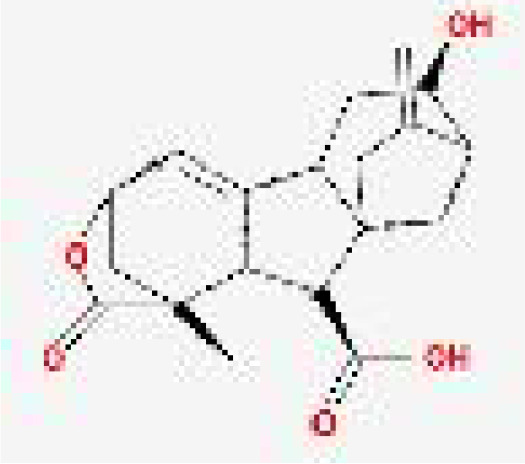
Taoren	MOL001329	2,3–didehydro GA77	346.41	88.08	0.53	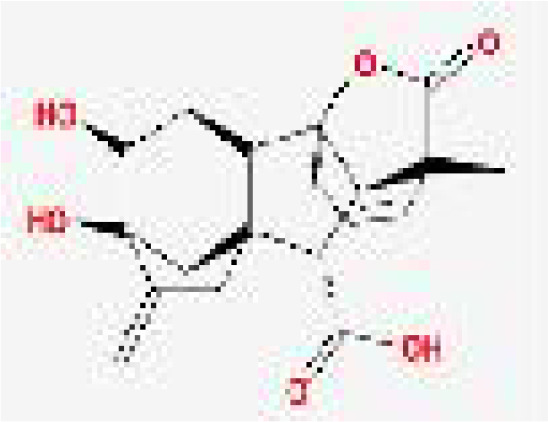
Taoren	MOL001360	GA77	348.43	87.89	0.53	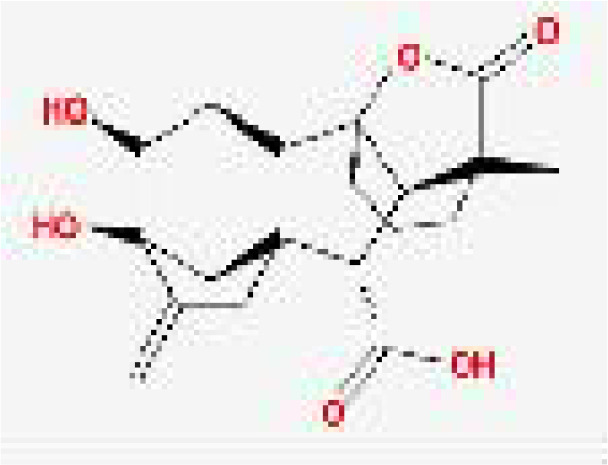
Yanhusuo	MOL004193	Clarkeanidine	327.41	86.65	0.54	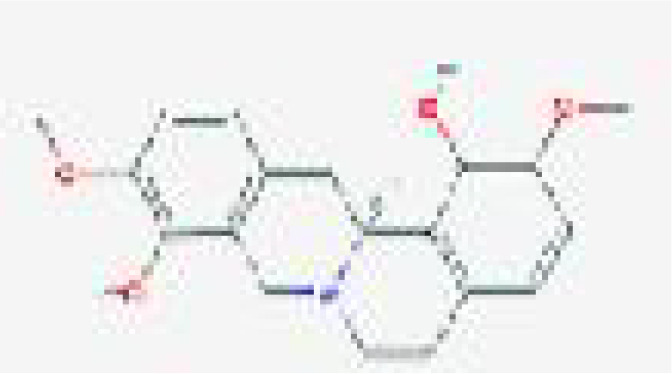

#### 3.1.2 Disease target selection and Venn diagram

**Figure 2:**
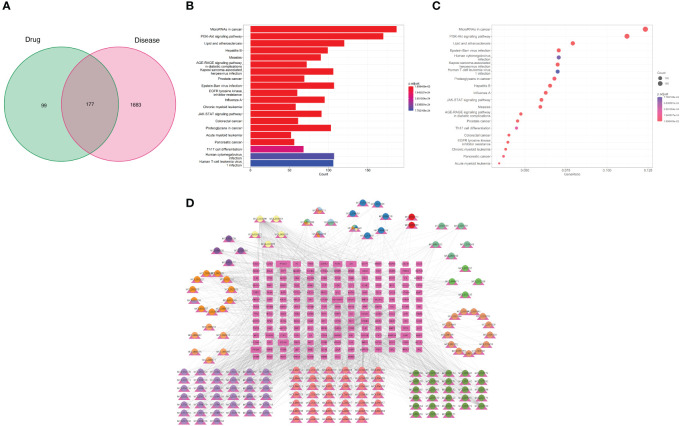
**(A)** Venn diagram of active compounds and AP–related targets. The green areas represent the number of active compound targets, the pink areas represent the number of AP–related targets, and the overlapping part is the number of their common target genes. **(B)** GO functional enrichment analysis to show the top 10 enriched BP, CC, and MF GO terms. **(C)** Generate a bubble chart illustrating the 20 most enriched KEGG pathways. **(D)** The “Drug–Ingredient–Target” network diagram predicts the active compounds of drugs and potential targets of CH effective in treating AP.

By eliminating duplicates, we acquired a grand total of 1986 disease targets from the GeneCards database using a relevance score of ≥10. Out of these, 2060 targets were obtained, including 143 targets from the OMIM database, 112 targets from the PharmGkb database, 9 targets from the TTD database, and 6 targets from the DrugBank database. We identified overlapping targets to select 177 potential therapeutic targets for treating AP between the compound and AP-related targets, as shown in **Supplementary Table S2**. The corresponding Venn diagram is shown in **Figure 2A**.

#### 3.1.3 GO and KEGG pathway enrichment analysis

DAVID’s GO analysis yielded three distinct categories: biological processes (BP), cellular constituents (CC), and molecular functions (MF), revealing a total of 4544, 269, and 439 identified terms in each category, respectively. The top 10 GO terms significantly enriched in each stratum can be found in **Figure 2B**. The results showed that BP was most related to epithelial cellular proliferation, CC was most related to the vesicle lumen, and MF was most related to mRNA blinding involved in post-transcriptional gene splicing.

In order to enhance our comprehension of the pharmacological mechanism of CH for AP treatment at the signaling pathway level, we conducted KEGG analysis. This analysis unveiled that the chosen 177 potential targets were significantly present in 186 pathways, encompassing the PI3K/AKT signaling pathway (hsa04151), AGE/AGE signaling pathway (hsa04933), JAK/TAT signaling pathway (hsa04630), and TNF signaling pathway (hsa04668). The top 30 most significantly enriched pathways are shown (P<0.05, ordered by gene count) in **Figure 2C**. A comprehensive overview of the PPI data is available in **Supplementary Table S3**.

#### 3.1.4 Construction and analysis of the D-C-T network

Cytoscape 3.9.1 was used to construct and visualize the D-C-T network of the active CH compounds and potential targets, which includes common targets between compound targets and AP-related targets. In this section, the 181 compounds linked to potential targets are presented in the network diagram. **Figure 2D** illustrates a network consisting of 358 nodes, including 181 compounds and 177 potential targets, connected by 1832 edges. The key compounds of CH can be considered as the ones that showed strong interactions in the C-T network. These compounds are from Baishao, Chaihu, Chishao, Dahuang, Danshen, Gancao, Houpu, Huangqin, Huangqi, Taoren, Yanhusuo, Zhishi, and Zhizi, with the numbers 3, 7, 8, 5, 28, 40, 2, 13, 7, 16, 42, 4, and 7, respectively. 148 different herb ingredients targeted prostaglandin G/H synthase 2 (PTGS2), while heat shock protein (HSP) 90-alpha (HSP90AA1), sodium channel protein type 5 subunit alpha (SCN5A), prostaglandin G/H synthase 1 (PTGS1), and nuclear receptor coactivator 2 (NCOA2) were targeted by 89, 83, 82, and 74 herb ingredients, respectively. This implies that the synergistic therapeutic effect on AP is achieved by multiple CH compounds.

**Figure 3:**
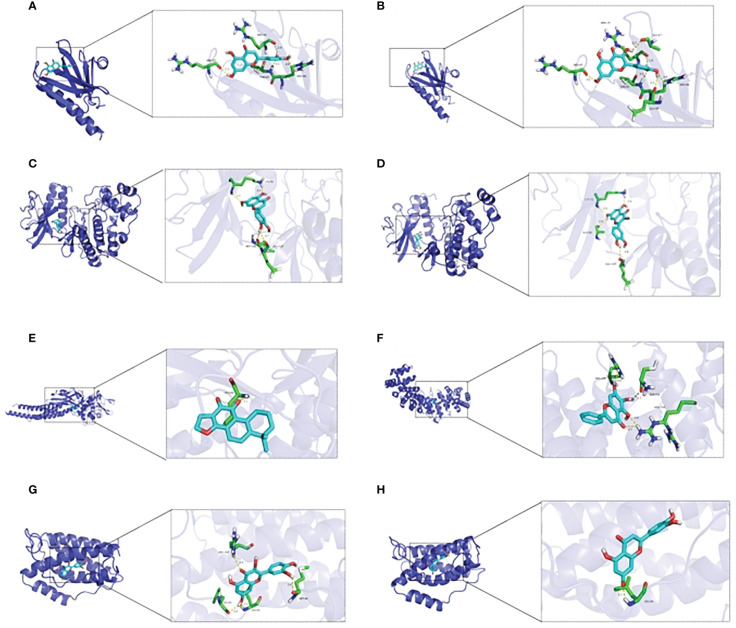
Molecular docking between essential elements of CH and crucial targets illustrated through schematic diagrams. The molecular docking diagrams of luteolin and AKT1, quercetin and AKT1, luteolin and MAPK1, quercetin and MAPK1, cryptotanshinone and STAT3, baicalein and HIF–1, quercetin and IL6, and luteolin and IL6 are represented by Note **(A–H)** respectively.

The ingredients of CH are represented by the outer nodes in **Figure 3**, while the common targets between the drug and the disease are represented by the middle nodes. The CH formula uses various colors to represent different herbal medicines: navy blue for Chaihu (Bupleuri Radix), crimson for Houpo (Magnoliae Officinalis Cortex), pale green for Chishao (Paeoniae Radix Rubra), vibrant green for Dahuang (Rhei Radix et Rhizoma), ochre yellow on the right for Taoren (Persicae Semen), dark green for Danshen (Salviae Miltiorrhizae Radix et Rhizoma), rose for Gancao (Glycyrrhizae Radix et Rhizoma), lavender for Yanhusuo (Corydalis Rhizoma), yellow on the left for Huangqi (Astragali Radix), golden yellow for Huangqin (Scutellariae Radix), deep violet for Zhishi (Aurantii Fructus Immaturus), pale yellow for Zhizi (Gardeniae Fructus), and sky blue for Baishao (Paeoniae Radix Alba).

#### 3.1.5 Construction and analysis of the PPI network

**Figure 4:**
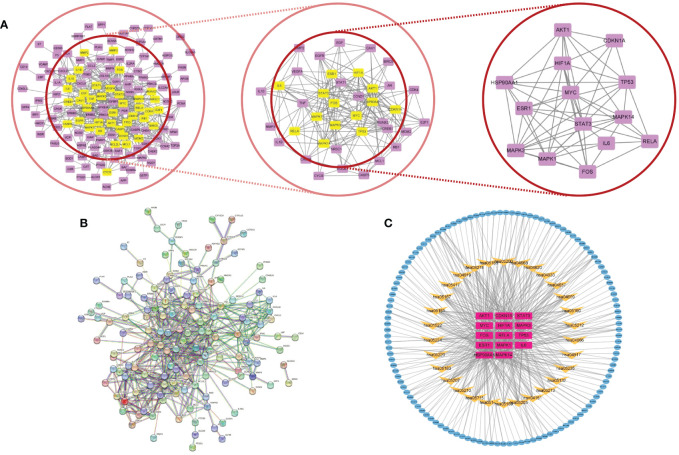
**(A)** Core subnetwork diagram of the target of CH for the treatment of AP. **(B)** PPI network of the target of CH for the treatment of AP. **(C)** The network called C–T–P is made up of 165 nodes and 485 edges. Blue nodes denote the active compounds. Yellow nodes denote the pathways, while purple nodes indicate core targets.

Utilization of the STRING database, a PPI network of the chosen 177 potential targets was established based on the aforementioned methods according to the minimum required interaction score ≥ 0.95 with isolated nodes hidden in the network in **Figure 4B**. In CytoNCA, different measures such as betweenness (BC), closeness (CC), degree (DC), eigenvector (EC), local average-connectivity-based methods (LAC), networks (NC), and information (IC) were utilized for the scoring and filtering procedures. Each gene was required to surpass its median value for retention. This scoring and filtering process was performed thrice in **Figure 4A**. Ultimately, 14 core target genes were identified (STAT3, IL6, MYC, CDKN1A, AKT1, MAPK1, MAPK3, MAPK14, HSP90AA1, HIF1A, ESR1, TP53, FOS, and RELA) comprising 14 nodes and 60 edges. Detailed information on PPI data is presented in **Supplementary Table S4**.

#### 3.1.6 Construction and analysis of the C-T-P network

The 14 core targets identified in the PPI network were linked to their corresponding compounds in the DCT network through the top 30 KEGG pathways, as depicted in **Figure 4C**. The C-T-P network that was obtained consisted of 165 nodes, comprising 121 compounds, 14 targets with potential, and 30 pathways for signaling. Additionally, there were 485 edges in the network. Additional examination uncovered that the chosen possible objectives had a strong connection with the PI3K/AKT (hsa04151, degree = 15), TNF (hsa04668, degree = 12), IL-17 (hsa04657, degree = 9), and HIF-1 (hsa04066, degree = 9) signaling pathways. The involvement of these pathways is crucial in the advancement of AP and various other inflammatory conditions.

#### 3.1.7 Molecular docking validation

From the C-T-P network, we identified the top three selected compounds and their corresponding target proteins, and conducted a molecular docking validation. Specifically, the target proteins chosen for docking overlapped with key target proteins in the PPI network. **Table 2** displays the outcomes of molecular docking. A lower docking score indicates a stronger ligand-receptor binding affinity, signifying a higher likelihood of interaction. All the binding energy scores between the active compounds and the key target proteins were less than -5.0 kJ/mol. The 3D binding models of each target protein and compound are shown in **Figure 3**. Additionally, the docking diagrams of the compound-target proteins suggested possible intermolecular interactions between the core compounds and protein targets.

**Table 2:** The affinity of compounds and targets.

Ligand	luteolin	quercetin	luteolin	quercetin	cryptotanshinone	baicalein	quercetin	luteolin
Protein	AKT1	AKT1	MAPK1	MAPK1	STAT3	HIF–1α	IL6	IL6
Affinity (kcal/mol)	–6.7	–7	–8	–7.9	–8.1	–7.2	–7.4	–7.4

### 3.2 The experimental verification of the network pharmacology results

#### 3.2.1 Effects of CH on pancreatic pathological changes

**Figure 5:**
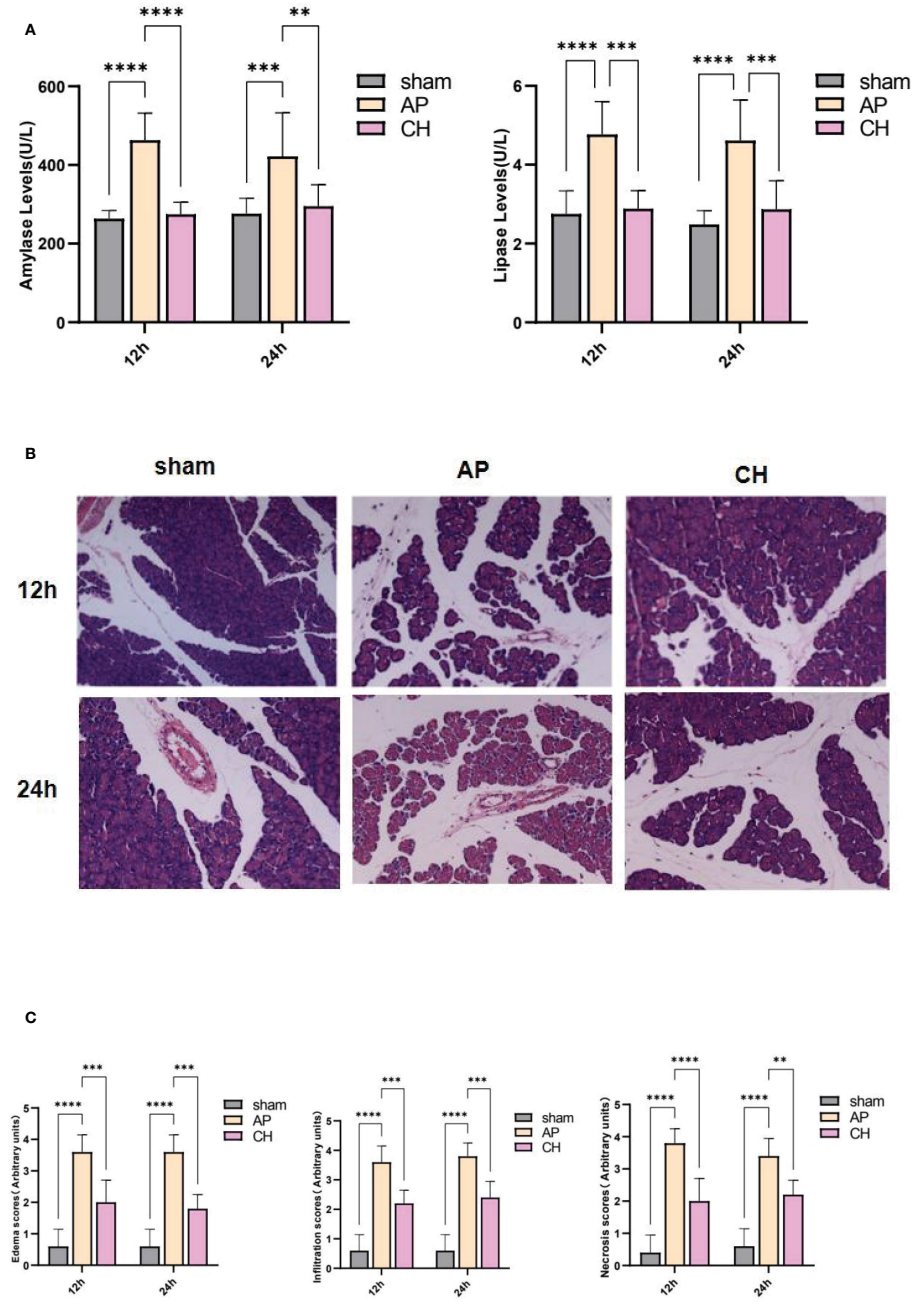
CH treatment attenuated AP. **(A)** Serum levels of amylase and lipase in different groups. The data presented above represent the average ± SD (*n* = 6) for each group, with **P < 0.01, ***P < 0.001, and ****P < 0.0001 compared to the AP group. **(B)** HE staining (200×) was used to examine the pancreatic pathology in rats from various groups. (*n* = 5). **(C)** Corresponding pathological score. The data presented above represent the average ± SD (*n* = 5) for each group, with **P < 0.01, ***P < 0.001, and ****P < 0.0001 compared to the AP group.

According to the findings shown in **Figure 5A**, the group with AP demonstrated significantly elevated serum amylase and lipase levels compared to the sham group. However, the administration of CH resulted in decreased levels. Moreover, the AP model group exhibited notably elevated levels of edema and inflammation compared to the group that underwent a sham operation. However, the administration of CH resulted in a decreased level of pancreatic pathological harm, which involved decreased swelling, inflammation, and tissue death, along with a decrease in pathological scores (*P* < 0.05) (**Figures 5B, C**). Collectively, these results demonstrate that CH alleviates pancreatic tissue inflammation and mitigates pathological damage in AP rats.

#### 3.2.2 Effect of CH on the expression of TNF-α, IL-6, and IL-1β

**Figure 6:**
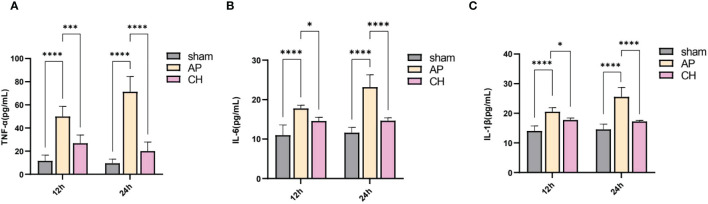
Effect of CH treatment on inflammation in AP rat model. **(A)** Serum levels of TNF–α among various groups. The data presented above are represented as the average ± SD (*n* = 5). ***P < 0.001 and ****P < 0.0001 compared to the AP group. **(B)** Serum levels of IL–6 among various groups. The data presented above are represented as the average ± SD (*n* = 5). *P < 0.05 and ****P < 0.0001 compared to the AP group. **(C)** Serum concentrations of IL–1β among various groups. The data presented above are represented as the average ± SD (*n* = 5). *P < 0.05 and ****P < 0.0001 compared to the AP group.

According to **Figure 6**, the AP group showed significantly raised levels of serum TNF-α, IL-6, and IL-1β in comparison with the sham group. However, the administration of CH resulted in a decrease of these levels. Over time, the levels of TNF-α, IL-6, and IL-1β in the AP group exhibited a substantial growth (*P* < 0.05).

#### 3.2.3 Effect of CH on the PI3K/AKT signaling pathway

**Figure 7:**
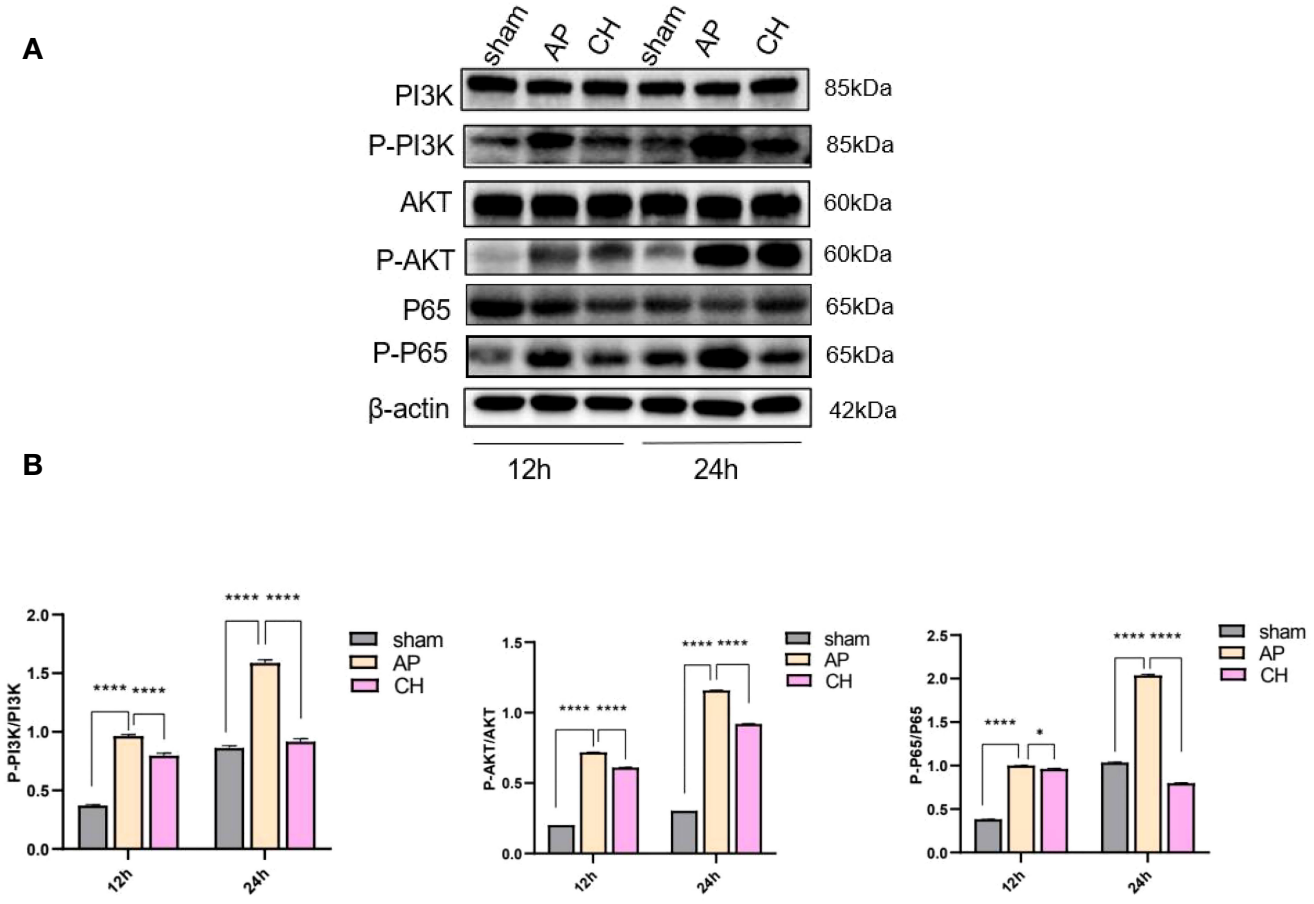
The impact of CH on the PI3K/AKT signaling pathway in the AP model. **(A)** Expression of PI3K, p–PI3K, AKT, p–AKT, P65, p–P65, and β–actin in different groups. (*n* = 3). **(B)** Ratios of p–PI3K/PI3K, p–AKT/AKT, and p–P65/P65 that correspond. The data presented above are represented as the average ± SD (*n* = 3). *P < 0.05 and ****P < 0.0001 compared to the AP group.

The levels of PI3K, AKT, and NF-ϰB proteins did not show any notable differences among the sham, AP, and CH groups (**Figure 7**). However, the AP model group exhibited considerably elevated levels of protein phosphorylation compared to the sham group. Conversely, the intervention group CH demonstrated notably reduced levels of phosphorylation among the proteins PI3K, AKT, and NF-ϰB.

#### 3.2.4 Effect of CH on cells apoptosis

**Figure 8:**
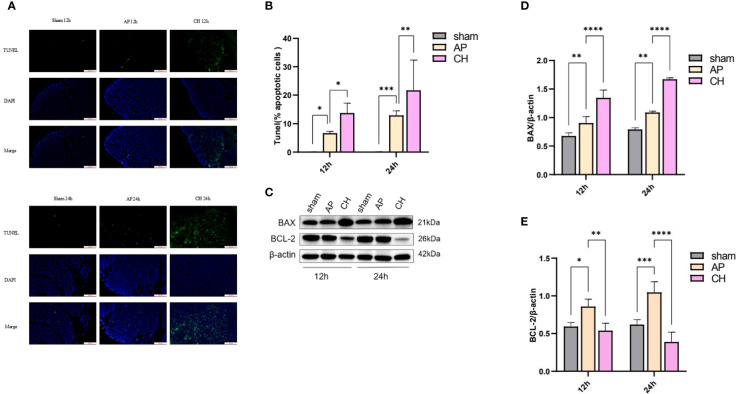
Administration of CH increases the apoptosis of pancreatic acinar cell in rats with AP. **(A)** Images from the TUNEL assay of pancreatic tissue, 100 μm scale bar. (*n* = 6). **(B)** Statistical results on the proportion of pancreatic acinar cells undergoing apoptosis in each group. Mean ± SD (*n* = 6) data were reported for each group, and statistical significance was observed. *P < 0.05, **P < 0.01, and ***P < 0.001 in comparison to the AP group. **(C)** Expression levels of BAX, BCL–2, and β–actin in various animal model groups.(*n* = 3). **(D)** Corresponding ratios of BAX/β–actin. Mean ± SD data were reported for each group, and statistical significance was observed (*n* = 3). **P < 0.01 and ****P < 0.0001 in comparison to the AP group. **(E)** Corresponding ratios of BCL–2/β–actin. Mean ± SD data were reported for each group, and statistical significance was observed (*n* = 3). *P < 0.05, **P < 0.01, ***P < 0.001, and ****P < 0.0001 in comparison to the AP group.

TUNEL staining was used to analyze apoptosis of acinar cells. Pancreatic acinar cells underwent apoptosis upon the induction of sodium taurocholate, as depicted in **Figure 8A**. After CH treatment, the incidence of TUNEL-positive cells has significantly increased, unlike the AP group (**Figure 8B**). Furthermore, the western blot analysis demonstrated that the CH group exhibited markedly elevated levels of the pro-apoptotic protein Bax, while displaying notably reduced levels of the anti-apoptotic protein BCL-2 in comparison to both the sham and AP groups. This indicates that CH significantly enhanced apoptosis in acute pancreatitis. (**Figures 8C–E**)

## 4 Discussion

AP is a common disorder marked by the activation of trypsinogen and abrupt inflammation of the pancreas (26). In clinical practice, treating complex diseases using a single targeted drug is challenging (27). Currently, drugs specific to AP are lacking, and most available treatments focus on symptom relief and supportive care (26). TCM is a highly effective method for treating AP due to its various benefits, including its ability to target multiple areas and its minimal adverse reactions (28, 29). Previous animal and clinical experiments have confirmed the therapeutic impact of CH on AP, yet the precise molecular mechanisms behind it remain unclear. To investigate the therapeutic effects of CH on AP, we employed a blend of network pharmacology and experimental validation techniques to examine the active constituents, targets, networks, and pathways involved. This approach aided our understanding of the material basis and molecular mechanisms underlying its benefits. We then conducted a series of experiments to confirm our findings using network pharmacology analysis. Lately, there has been a global focus on utilizing network pharmacology for the treatment of illnesses. Network pharmacology is an efficient method for identifying ingredients and forecasting drug targets using advanced computer simulations. This study explored the potential use of network pharmacology to understand how CH can effectively and affordably treat AP. Using network pharmacology, we investigated the basic elements and mechanisms underlying the therapeutic effects of CH on AP.

TCMSP retrieval yielded 181 active compounds. The analysis of the D–C–T network demonstrated that there were 177 interconnected potential targets, indicating that CH might affect AP through mechanisms involving multiple compounds and targets. Additionally, PPI network analysis identified 14 core genes among the potential targets, including STAT3, IL6, MYC, CDKN1A, AKT1, MAPK1, MAPK14, HSP90AA1, HIF1, ESR1, TP53, FOS, and RELA. AKT1, MAPK1, MAPK3, MAPK14, IL6, STAT1, and HIF1 are closely related to AP based on wide–scale literature mining.

The precise pathophysiological mechanisms responsible for AP are still not fully understood. For this study, we conducted GO and KEGG investigations to uncover the potential molecular pathways involved in CH. Our findings validate the involvement of diverse biological processes and signaling pathways in the commencement and advancement of AP. The PI3K/AKT signaling pathway, which is one of the pathways enriched and connected to the network, is recognized as a significant contributor to inflammation associated with AP (27, 30, 31). Researchers (32–34) have also considered several other pathways as possible targets for AP treatment. These pathways include the AGE/RAGE signaling pathway (hsa04933), JAK/STAT signaling pathway (hsa04630), and TNF signaling pathway (hsa04668). The active compounds show favorable binding activity with the essential proteins of CH, as indicated by the molecular docking results.

Based on the above analysis, we performed further experimental verification and confirmed the protective effect of CH against AP. Elevated amylase and lipase are the hallmarks of AP. Combined with the pathological staining results, we also confirmed that CH reduced pancreatic injury in AP. Additionally, using western blot and Tunel staining, we further verified CH’s ability to induce apoptosis in acinar cells through the PI3K/AKT pathway, thereby alleviating severe pancreatitis.

During the initial stages of AP, acinar cell death via necrosis and apoptosis significantly affects the progression of the condition. Recent research (35) has shown that apoptosis may serve as a protective response in AP. Furthermore, studies (26, 36) have shown that triggering apoptosis in acinar cells can decrease the activation and secretion of trypsin, consequently lowering the incidence of systemic inflammatory response syndrome (SIRS). Several signaling pathways are involved in apoptosis. The PI3K/AKT pathway is essential in averting cell death, and blocking it results in higher levels of programmed cell death in pancreatic cells. This, in turn, reduces pancreatic harm and enhances the chances of survival in AP rats (37). Previous studies (38) have extensively confirmed the role of the PI3K/AKT signaling pathway in the process of inflammation, which is strongly linked to NF–ϰB and IL–6. PI3Ks play vital roles in mediating inflammatory responses. Lupia et al. (39) revealed that by deleting the PI3K gene in rats, there was a significant reduction in the damage and mortality of pancreatic acinar cells compared to rats with the wild–type gene. Earlier research (40–43) has suggested that PI3K, by activating AKT, has the ability to boost the movement of NF–ϰB into the nucleus and its activity in transcription, which is crucial in the progression of Severe Acute Pancreatitis. Furthermore, activation of the PI3K/AKT pathway was observed in both *in vivo* experiments with AP–induced inflammation and *in vitro* studies with cytokine administration. Further investigations (44) revealed that inhibiting PI3K/AKT or NF–κB could enhance the survival rates of rats with AP. These results indicate that blocking PI3K may offer the potential for preventing and treating AP. Consistent with these research, our study has shown that CH intervention effectively suppressed the activation of the PI3K/AKT pathway, resulting in the promotion of apoptosis in pancreatic acinar cells.

This investigation has certain limitations that merit further investigation. The sample size of AP–related targets in the GEO database (45) should be increased. Additionally, the potentially active compounds identified in this study require independent validation of their therapeutic efficacy against AP.

## 5 Conclusions

Despite the unclear comprehension of the complex pharmacological mechanisms implicated in the effectiveness of CH for treating AP, this study sought to forecast the responsible mechanisms for the defensive benefits of CH against AP through a network pharmacology–driven approach. The findings from our study suggest that CH may achieve its healing benefits by utilizing a variety of elements, objectives, and routes. The network pharmacology method holds the potential to uncover the molecular mechanisms of Traditional Chinese Medicine (TCM), including CH, by presenting valuable insights for clinical application of this formulation.

## Data availability statement

The original contributions presented in the study are included in the article/**Supplementary Materials**, further inquiries can be directed to the corresponding author/s.

## Ethics statement

The animal study was approved by The Animal Ethics Committee of the Southwest Medical University. Affiliations: Southwest Medical University. The study was conducted in accordance with the local legislation and institutional requirements. No potentially identifiable images or data are presented in this study.

## Author contributions

JY: Conceptualization, Data curation, Formal analysis, Investigation, Methodology, Writing – original draft, Writing – review & editing. Y–HJ: Conceptualization, Data curation, Visualization, Writing – original draft. XZ: Funding acquisition, Validation, Writing – review & editing. J–QY: Validation, Writing – review & editing. Y–YW: Investigation, Writing – original draft. J–QL: Methodology, Writing – review & editing. P–CZ: Writing – review & editing. W–FT: Writing – review & editing. ZL: Writing – review & editing.

## References

[1] Boxhoorn L Voermans RP Bouwense SA Bruno MJ Verdonk RC Boermeester MA. Acute pancreatitis. Lancet. (2020) 396:726–34. doi: 10.1016/S0140-6736(20)31310-6 32891214

[2] Boumitri C Brown E Kahaleh M. Necrotizing pancreatitis: current management and therapies. Clin Endosc. (2017) 50:357–65. doi: 10.5946/ce.2016.152 PMC5565044 28516758

[3] Chan YC Leung PS. Acute pancreatitis: animal models and recent advances in basic research. Pancreas. (2007) 34:1–14. doi: 10.1097/01.mpa.0000246658.38375.04 17198179

[4] Ma Y Huang ZW Xia Q Xue P Guo J Wei HQ. [Influence of integrated traditional Chinese and Western medicine therapy on serum resistin levels in patients with severe acute pancreatitis: a randomized controlled trial]. Zhong Xi Yi Jie He Xue Bao. (2009) 7:1134–8. doi: 10.3736/jcim 20015433

[5] Wang L Li Y Ma Q Liu Y Rui YY Xue P. Chaiqin Chengqi Decoction decreases IL–6 levels in patients with acute pancreatitis. J Zhejiang Univ Sci B. (2011) 12:1034–40. doi: 10.1631/jzus.B1000406 PMC3232437 22135153

[6] Shi R Xiao GH Feng W Yu Y Wang TG. The clinical efficacy and related cytokines of Chaihuang Qingyi Huoxue Granule in patients with severe acute pancreatitis. World Latest Med Inf. (2019) 19:28–30. doi: 10.19613/j.cnki.1671-3141.2019.61.013

[7] Li XY Lu J Fu J Zhou X Tang ZX Li L. Mechanism of chaihuang qingyi huoxue granules against pancreatic microcirculatory disturbance in severe acutePancreatitis rats through regulating KEAP1/NRF2 signal pathway. Pharmacol Clinics Chin Materia Med. (2023) 39:26–31. doi: 10.13412/j.cnki.zyyl.20230313.001

[8] Tang ZX Zuo XH Fu J Lu J Zhao L Li L. Effect of chaihuang qingyi huoxue granules on JAK2/STAT3 signal pathway in rats with severe acute pancreatitis. Pharmacol Clinics Chin Materia Med. (2022) 33:1055–62. 10.19378/j.issn.1003-9783.2022.08.008

[9] Ru J Li P Wang J Zhou W Li B Huang C. TCMSP: a database of systems pharmacology for drug discovery from herbal medicines. J Cheminform. (2014) 6:13. doi: 10.1186/1758-2946-6-13 24735618 PMC4001360

[10] Butkiewicz M Wang Y Bryant SH Lowe EW Jr Weaver DC Meiler J. High–throughput screening assay datasets from the pubChem database. Chem Inform. (2017) 3:1. doi: 10.21767/2470-6973 29795804 PMC5962024

[11] Xu X Zhang W Huang C Li Y Yu H Wang Y. A novel chemometric method for the prediction of human oral bioavailability. Int J Mol Sci. (2012) 13:6964–82. doi: 10.3390/ijms13066964 PMC3397506 22837674

[12] Wishart DS Feunang YD Guo AC Lo EJ Marcu A Grant JR. DrugBank 5.0: a major update to the DrugBank database for 2018. Nucleic Acids Res. (2018) 46:D1074–82. doi: 10.1093/nar/gkx1037 PMC5753335 29126136

[13] Zaru R Orchard S UniProt Consortium. UniProt tools: BLAST, align, peptide search, and ID mapping. Curr Protoc. (2023) 3:e697. doi: 10.1002/cpz1.697 36943033 PMC10034637

[14] Barshir R Fishilevich S Iny–Stein T Zelig O Mazor Y Guan–Golan Y. GeneCaRNA: A comprehensive gene–centric database of human non–coding RNAs in the geneCards suite. J Mol Biol. (2021) 433:166913. doi: 10.1016/j.jmb.2021.166913 33676929

[15] Hamosh A Amberger JS Bocchini C Scott AF Rasmussen SA. Online Mendelian Inheritance in Man (OMIM^®^): Victor McKusick’s magnum opus. Am J Med Genet A. (2021) 185:3259–65. doi: 10.1002/ajmg.a.62407 PMC8596664 34169650

[16] Gong L Whirl–Carrillo M Klein TE. PharmGKB, an integrated resource of pharmacogenomic knowledge. Curr Protoc. (2021) 1:e226. doi: 10.1002/cpz1.226 34387941 PMC8650697

[17] Zhou Y Zhang Y Zhao D Yu X Shen X Zhou Y. TTD: Therapeutic Target Database describing target druggability information. Nucleic Acids Res. (2023) 52(D1):gkad751. doi: 10.1093/nar/gkad751 PMC10767903 37713619

[18] Zhu F Han B Kumar P Liu X Ma X wei X. Update of TTD: therapeutic target database. Nucleic Acids Res. (2010) 38:D787–91. doi: 10.1093/nar/gkp1014 PMC2808971 19933260

[19] Huang DW Sherman BT Tan Q Kir J Liu D Bryant D. DAVID Bioinformatics Resources: expanded annotation database and novel algorithms to better extract biology from large gene lists. Nucleic Acids Res. (2007) 35:W169–75. doi: 10.1093/nar/gkm415 PMC1933169 17576678

[20] Otasek D Morris JH Bouças J Pico AR Demchak B. Cytoscape Automation: empowering workflow–based network analysis. Genome Biol. (2019) 20:185. doi: 10.1186/s13059-019-1758-4 31477170 PMC6717989

[21] Szklarczyk D Kirsch R Koutrouli M Nastou K Mehryary F Hachilif R. The STRING database in 2023: protein–protein association networks and functional enrichment analyses for any sequenced genome of interest. Nucleic Acids Res. (2023) 51:D638–46. doi: 10.1093/nar/gkac1000 PMC9825434 36370105

[22] Eberhardt J Santos–Martins D Tillack AF Forli S. AutoDock vina 1.2.0: new docking methods, expanded force field, and python bindings. J Chem Inf Model. (2021) 61:3891–8. doi: 10.1021/acs.jcim.1c00203 PMC10683950 34278794

[23] Desaphy J Bret G Rognan D Kellenberger E. sc–PDB: a 3D–database of ligandable binding sites–10 years on. Nucleic Acids Res. (2015) 43:D399–404. doi: 10.1093/nar/gku928 PMC4384012 25300483

[24] Zhang YM Ren HY Zhao XL Li J Li JY Wu FS. Pharmacokinetics and pharmacodynamics of Da–Cheng–Qi decoction in the liver of rats with severe acute pancreatitis. World J Gastroenterol. (2017) 23:1367–74. doi: 10.3748/wjg.v23.i8.1367 PMC5330821 28293083

[25] Schmidt J Rattner DW Lewandrowski K Compton CC Mandavilli U Knoefel WT. A better model of acute pancreatitis for evaluating therapy. Ann Surg. (1992) 215:44–56. doi: 10.1097/00000658-199201000-00007 1731649 PMC1242369

[26] Mederos MA Reber HA Girgis MD. Acute pancreatitis: A review. JAMA. (2021) 325:382–90. doi: 10.1001/jama.2020.20317 33496779

[27] Yuan J Tan T Geng M Tan G Chheda C Pandol SJ. Novel small molecule inhibitors of protein kinase D suppress NF–kappaB activation and attenuate the severity of rat cerulein pancreatitis. Front Physiol. (2017) 8:1014. doi: 10.3389/fphys.2017.01014 29270134 PMC5725929

[28] Yang X Liu Y Zhong C Hu J Xu S Zhang P. Total flavonoids of Chrysanthemum indicum L inhibit acute pancreatitis through suppressing apoptosis and inflammation. BMC Complement Med Ther. (2023) 23:23. doi: 10.1186/s12906-023-03851-x 36709296 PMC9883918

[29] Sun W Chen Y Li H Liu H Li J Chen J. Material basis and molecular mechanisms of Dachengqi decoction in the treatment of acute pancreatitis based on network pharmacology. BioMed Pharmacother. (2020) 121:109656. doi: 10.1016/j.biopha.2019.109656 31810129

[30] Jin Y Liu L Chen B Bai Y Zhang F Li Q. Involvement of the PI3K/akt/NF–κB signaling pathway in the attenuation of severe acute pancreatitis–associated acute lung injury by sedum sarmentosum bunge extract. BioMed Res Int. (2017) 2017:9698410. doi: 10.1155/2017/9698410 29359164 PMC5735615

[31] Rao CY Fu LY Hu CL Chen DX Gan T Wang YC. H2S mitigates severe acute pancreatitis through the PI3K/AKT–NF–κB pathway *in vivo* . World J Gastroenterol. (2015) 21:4555–63. doi: 10.3748/wjg.v21.i15.4555 PMC4402302 25914464

[32] Lesina M Wörmann SM Neuhöfer P Song L Algül H. Interleukin–6 in inflammatory and Malignant diseases of the pancreas. Semin Immunol. (2014) 26:80–7. doi: 10.1016/j.smim.2014.01.002 24572992

[33] Sharma A Kaur S Sarkar M Sarin BC Changotra H. The AGE–RAGE axis and RAGE genetics in chronic obstructive pulmonary disease. Clin Rev Allergy Immunol. (2021) 60:244–58. doi: 10.1007/s12016-020-08815-4 33170477

[34] Li ST Dai Q Zhang SX Liu YJ Yu QQ Tan F. Ulinastatin attenuates LPS–induced inflammation in mouse macrophage RAW264.7 cells by inhibiting the JNK/NF–κB signaling pathway and activating the PI3K/Akt/Nrf2 pathway. Acta Pharmacol Sin. (2018) 39:1294–304. doi: 10.1038/aps.2017.143 PMC6289329 29323338

[35] Sendler M Mayerle J Lerch MM. Necrosis, apoptosis, necroptosis, pyroptosis: it matters how acinar cells die during pancreatitis. Cell Mol Gastroenterol Hepatol. (2016) 2:407–8. doi: 10.1016/j.jcmgh.2016.05.007 PMC5042603 28174728

[36] Mareninova OA Sung KF Hong P Lugea A Pandol SJ Gukovsky I. Cell death in pancreatitis: caspases protect from necrotizing pancreatitis. J Biol Chem. (2006) 281:3370–81. doi: 10.1074/jbc.M511276200 16339139

[37] Sarker RS Steiger K. A critical role for Akt1 signaling in acute pancreatitis progression†. J Pathol. (2020) 251:1–3. doi: 10.1002/path.5391 32003469

[38] Sailai Y Yu X Baiheti P Tang H Li Y Xu M. Influence of nuclear factor kappaB activation on inflammatory mediators of alveolar macrophages in rats with acute necrotizing pancreatitis. J Investig Med. (2010) 58:38–42. doi: 10.2310/JIM.0b013e3181b91bd6 19730128

[39] Lupia E Goffi A De Giuli P Azzolino O Bosco O Patrucco E. Ablation of phosphoinositide 3–kinase–gamma reduces the severity of acute pancreatitis. Am J Pathol. (2004) 165:2003–11. doi: 10.1016/S0002-9440(10)63251-8 PMC1618701 15579443

[40] Yum HK Arcaroli J Kupfner J Shenkar R Penninger JM Sasaki T. Involvement of phosphoinositide 3–kinases in neutrophil activation and the development of acute lung injury. J Immunol. (2001) 167:6601–8. doi: 10.4049/jimmunol.167.11.6601 11714830

[41] Kim KH Yang CS Shin AR Jeon SR Park JK Kim HJ. Mycobacterial heparin–binding hemagglutinin antigen activates inflammatory responses through PI3–K/akt, NF–κB, and MAPK pathways. Immune Netw. (2011) 11:123–33. doi: 10.4110/in.2011.11.2.123 PMC3100523 21637390

[42] Reddy SA Huang JH Liao WS. Phosphatidylinositol 3–kinase as a mediator of TNF–induced NF–kappa B activation. J Immunol. (2000) 164:1355–63. doi: 10.4049/jimmunol.164.3.1355 10640750

[43] Chen J Chen J Wang X Wang C Cao W Zhao Y. Ligustrazine alleviates acute pancreatitis by accelerating acinar cell apoptosis at early phase via the suppression of p38 and Erk MAPK pathways. BioMed Pharmacother. (2016) 82:1–7. doi: 10.1016/j.biopha.2016.04.048 27470331

[44] Liao D Qian B Zhang Y Wu K Xu M. Inhibition of 5–lipoxygenase represses neutrophils activation and activates apoptosis in pancreatic tissues during acute necrotizing pancreatitis. Biochem Biophys Res Commun. (2018) 498:79–85. doi: 10.1016/j.bbrc.2018.02.026 29421656

[45] Toro–Domínguez D Martorell–Marugán J López–Domínguez R García–Moreno A González–Rumayor V Alarcón–Riquelme ME. ImaGEO: integrative gene expression meta–analysis from GEO database. Bioinformatics. (2019) 35:880–2. doi: 10.1093/bioinformatics/bty721 30137226

