# Corrigendum: Material basis and molecular mechanisms of Chaihuang Qingyi Huoxue Granule in the treatment of acute pancreatitis based on network pharmacology and molecular docking-based strategy

**DOI:** 10.3389/fimmu.2024.1429862

**Published:** 2024-06-24

**Authors:** Jia Yang, Yu-Hong Jiang, Xin Zhou, Jia-Qi Yao, Yang-Yang Wang, Jian-Qin Liu, Peng-Cheng Zhang, Wen-Fu Tang, Zhi Li

**Affiliations:** ^1^ School of Integrated Traditional Chinese and Western Medicine, Southwest Medical University, Luzhou, Sichuan, China; ^2^ Department of Integrated Traditional Chinese and Western Medicine, National Clinical Research Center for Geriatrics, West China Hospital, Sichuan University, Chengdu, China; ^3^ Department of Spleen and Stomach Diseases, Chinese Medicine Hospital Affiliated to Southwest Medical University, Luzhou, Sichuan, China; ^4^ The Key Laboratory of Integrated Traditional Chinese and Western Medicine for Prevention and Treatment of Digestive System Diseases of Luzhou city, Affiliated Traditional Medicine Hospital of Southwest Medical University, Luzhou, China

**Keywords:** acute pancreatitis, Chaihuang Qingyi Huoxue Granule, network pharmacology, molecular docking, pancreatic acinar cells, Traditional Chinese

**Error in Figure/Table Legend**

In the published article, there was an error in the legend for **Figure 8E** as published. The correct graph should display BCL-2/b-actin data, but due to an oversight, it currently shows the same data as **Figure 8D** (BAX/b-actin). We apologize for any confusion this may have caused and are committed to rectifying this issue promptly. The corrected figure appears below.

**Figure 8:**
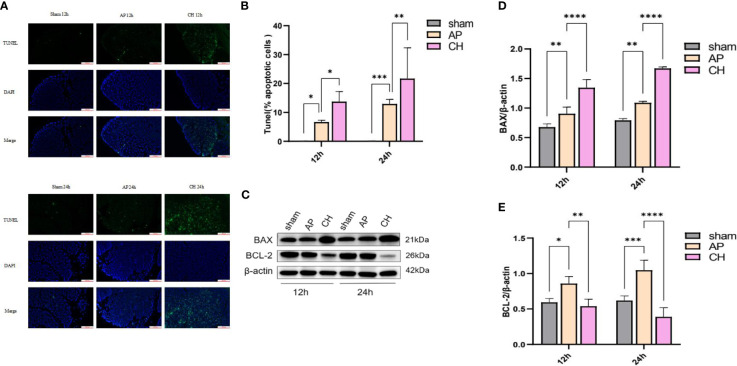
Administration of CH increases the apoptosis of pancreatic acinar cell in rats with AP. **(A)** Images from the TUNEL assay of pancreatic tissue, 100 μm scale bar. (*n* = 6). **(B)** Statistical results on the proportion of pancreatic acinar cells undergoing apoptosis in each group. Mean ± SD (n = 6) data were reported for each group, and statistical significance was observed. *P < 0.05, **P < 0.01, and ***P < 0.001 in comparison to the AP group. **(C)** Expression levels of BAX, BCL–2, and β–actin in various animal model groups. (*n* = 3). **(D)** Corresponding ratios of BAX/β–actin. Mean ± SD data were reported for each group, and statistical significance was observed (*n* = 3). **P < 0.01 and ****P < 0.0001 in comparison to the AP group. **(E)** Corresponding ratios of BCL–2/β–actin. Mean ± SD data were reported for each group, and statistical significance was observed (*n* = 3). *P < 0.05, **P < 0.01, ***P < 0.001, and ****P < 0.0001 in comparison to the AP group.

The authors apologize for this error and state that this does not change the scientific conclusions of the article in any way. The original article has been updated.

