# Supplementary material for: Material basis and molecular mechanisms of Chaihuang Qingyi Huoxue Granule in the treatment of acute pancreatitis based on network pharmacology and molecular docking-based strategy
Source: Front Immunol. 2024 May 3;15:1353695. doi: 10.3389/fimmu.2024.1353695 (PMC11099290; doi:10.3389/fimmu.2024.1353695)
Supplement: Supplementary file 2 [file DataSheet_3.zip › Figures+Tables/Table/Table 2.docx]

**Table 2. The affinity of compounds and targets**

| ligand | luteolin | quercetin | luteolin | quercetin | cryptotanshinone | baicalein | quercetin | luteolin |
| --- | --- | --- | --- | --- | --- | --- | --- | --- |
| protein | AKT1 | AKT1 | MAPK1 | MAPK1 | STAT3 | HIF-1α | IL6 | IL6 |
| Affinity (kcal/mol) | -6.7 | -7 | -8 | -7.9 | -8.1 | -7.2 | -7.4 | -7.4 |
