# Supplementary figures and images for: Material basis and molecular mechanisms of Chaihuang Qingyi Huoxue Granule in the treatment of acute pancreatitis based on network pharmacology and molecular docking-based strategy
Source: Front Immunol. 2024 May 3;15:1353695. doi: 10.3389/fimmu.2024.1353695 (PMC11099290; doi:10.3389/fimmu.2024.1353695)

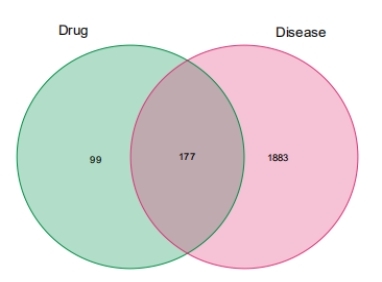

Supplement: Supplementary file 2 [file DataSheet_3.zip › Figures+Tables/Fig. 2/Fig. 2.jpg]

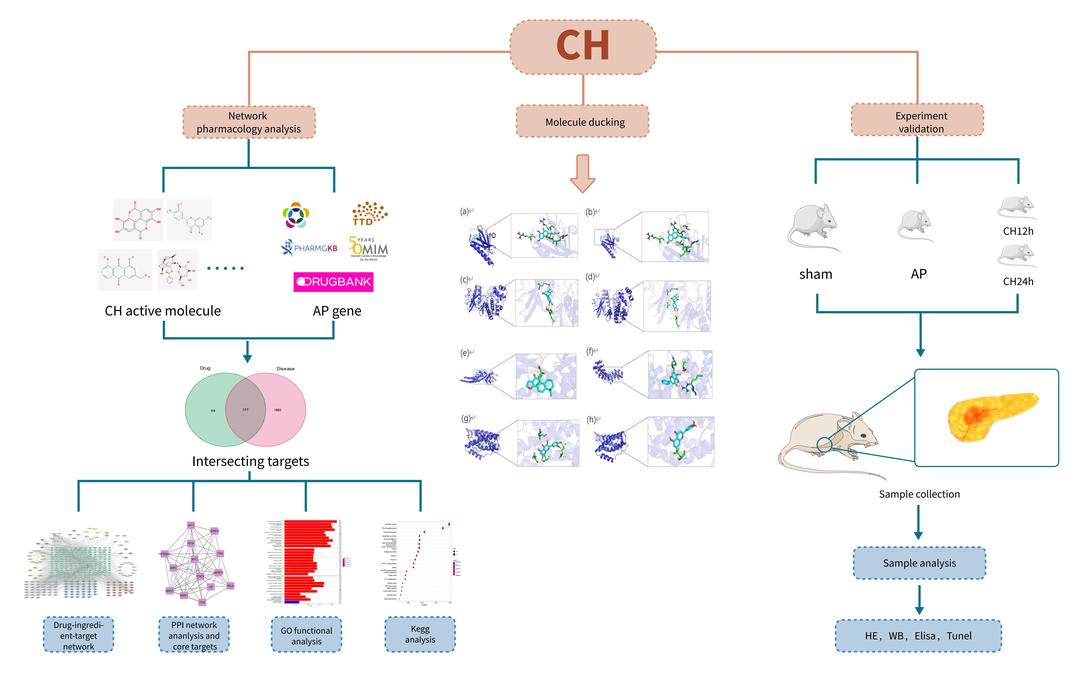

Supplement: Supplementary file 2 [file DataSheet_3.zip › Figures+Tables/Fig.1.jpg]

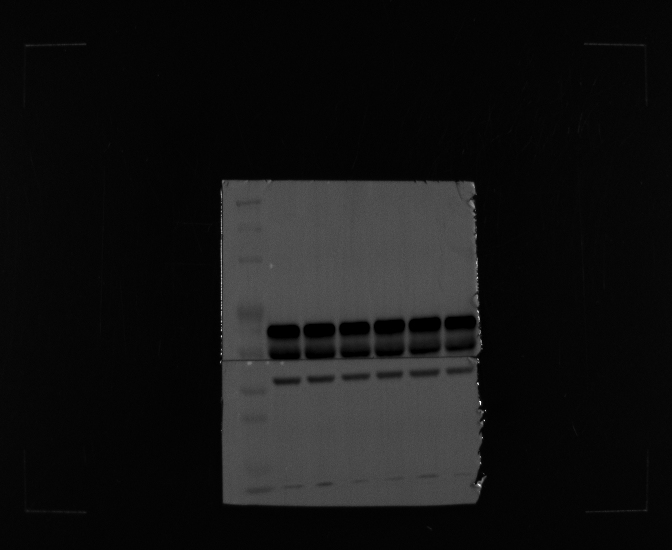

Supplement: Supplementary file 2 [file DataSheet_3.zip › Figures+Tables/Fig.10/Fig.10.(A)/AKT 60kDa/AKT 60kDa+b-actin 42kDa 2.1.jpg]

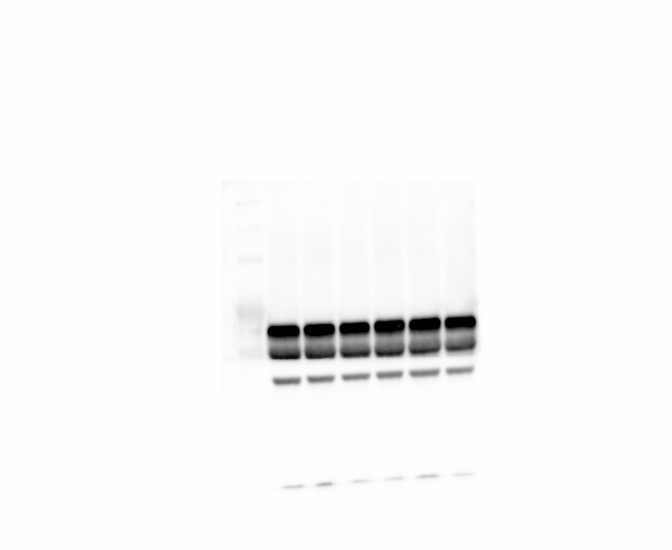

Supplement: Supplementary file 2 [file DataSheet_3.zip › Figures+Tables/Fig.10/Fig.10.(A)/AKT 60kDa/AKT 60kDa+b-actin 42kDa 2.jpg]

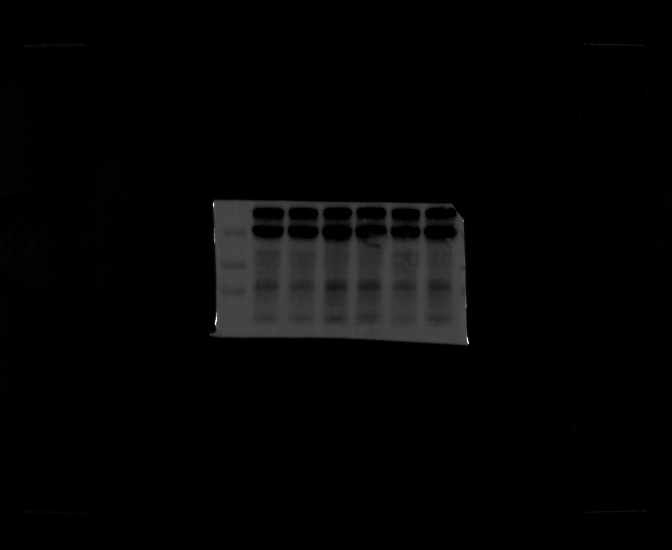

Supplement: Supplementary file 2 [file DataSheet_3.zip › Figures+Tables/Fig.10/Fig.10.(A)/AKT 60kDa/akt 60kDa 1.1.jpg]

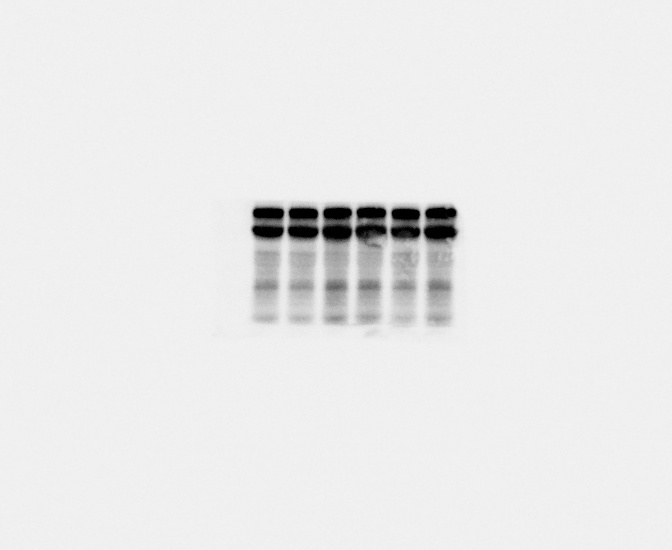

Supplement: Supplementary file 2 [file DataSheet_3.zip › Figures+Tables/Fig.10/Fig.10.(A)/AKT 60kDa/akt 60kDa 1.jpg]

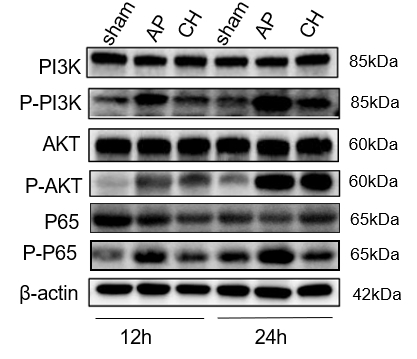

Supplement: Supplementary file 2 [file DataSheet_3.zip › Figures+Tables/Fig.10/Fig.10.(A)/Fig.10.(A).jpg]

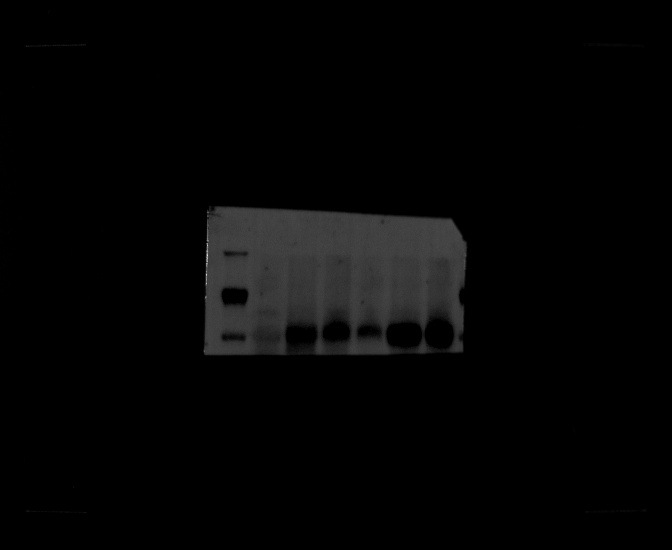

Supplement: Supplementary file 2 [file DataSheet_3.zip › Figures+Tables/Fig.10/Fig.10.(A)/P-AKT 60kDa/P-akt 60kDa 1.1.jpg]

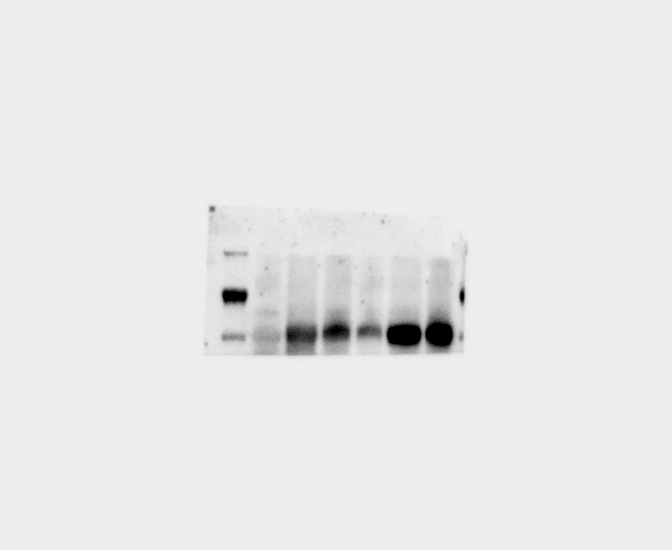

Supplement: Supplementary file 2 [file DataSheet_3.zip › Figures+Tables/Fig.10/Fig.10.(A)/P-AKT 60kDa/P-akt 60kDa 1.jpg]

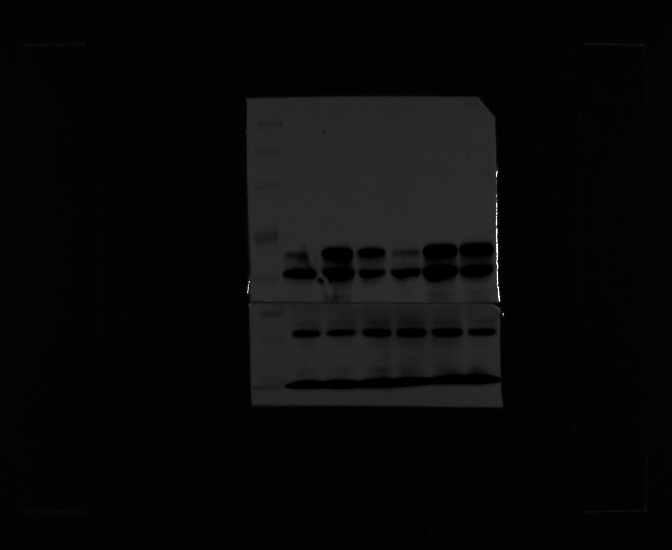

Supplement: Supplementary file 2 [file DataSheet_3.zip › Figures+Tables/Fig.10/Fig.10.(A)/P-AKT 60kDa/p-akt 60kDa+GAPDH 35kDa 2.1.jpg]

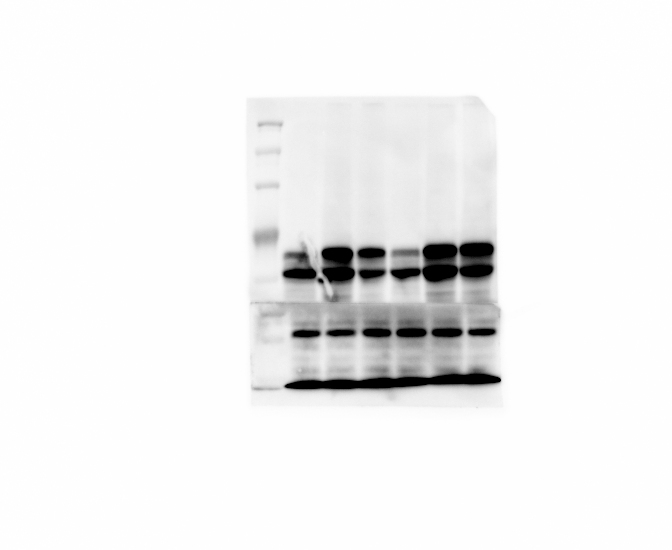

Supplement: Supplementary file 2 [file DataSheet_3.zip › Figures+Tables/Fig.10/Fig.10.(A)/P-AKT 60kDa/p-akt 60kDa+GAPDH 35kDa 2.jpg]

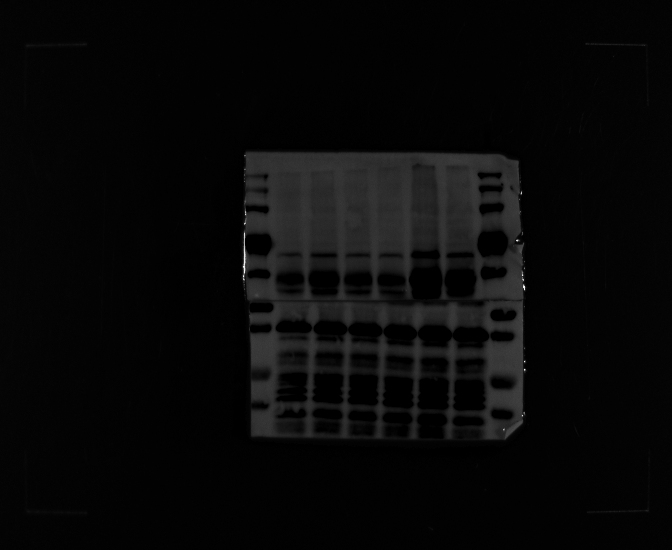

Supplement: Supplementary file 2 [file DataSheet_3.zip › Figures+Tables/Fig.10/Fig.10.(A)/P-P65 65kDa/p-p65 65kDa-GAPDH 35kDa 2.1.jpg]

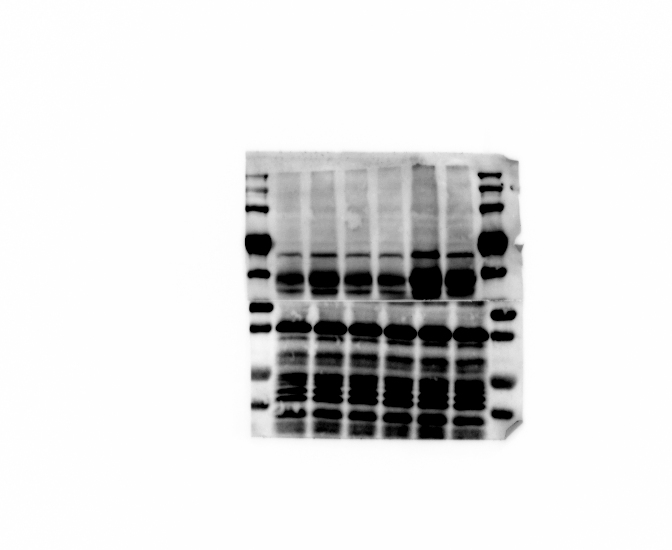

Supplement: Supplementary file 2 [file DataSheet_3.zip › Figures+Tables/Fig.10/Fig.10.(A)/P-P65 65kDa/p-p65 65kDa-GAPDH 35kDa 2.jpg]

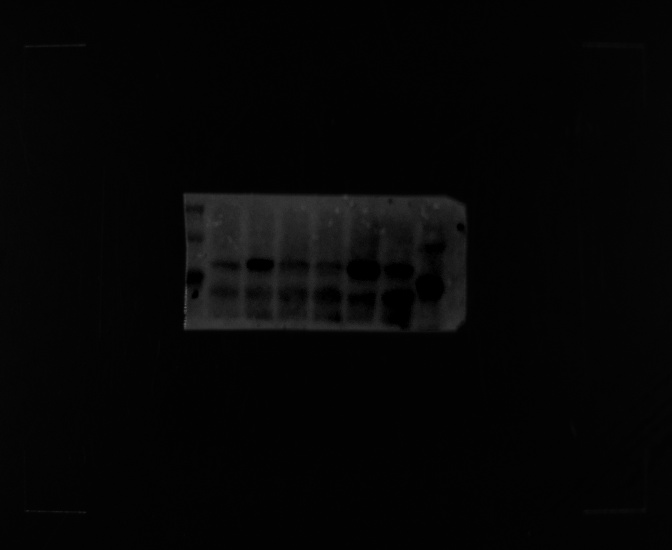

Supplement: Supplementary file 2 [file DataSheet_3.zip › Figures+Tables/Fig.10/Fig.10.(A)/P-PI3K 85kDa/p-pi3k 85kDa 1.1.jpg]

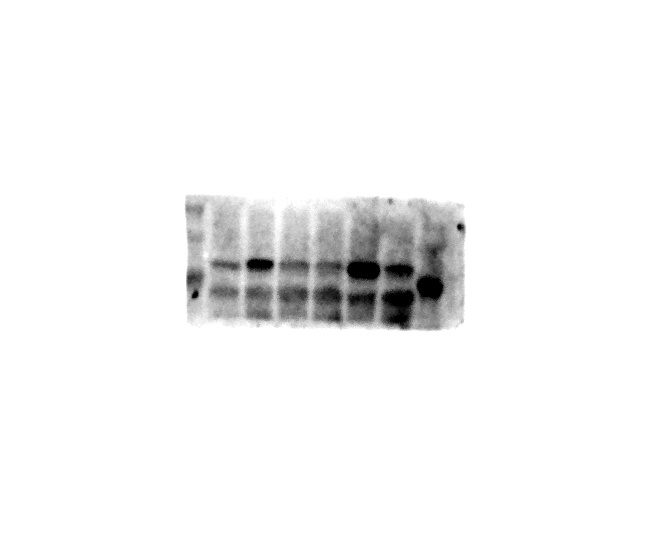

Supplement: Supplementary file 2 [file DataSheet_3.zip › Figures+Tables/Fig.10/Fig.10.(A)/P-PI3K 85kDa/p-pi3k 85kDa 1.jpg]

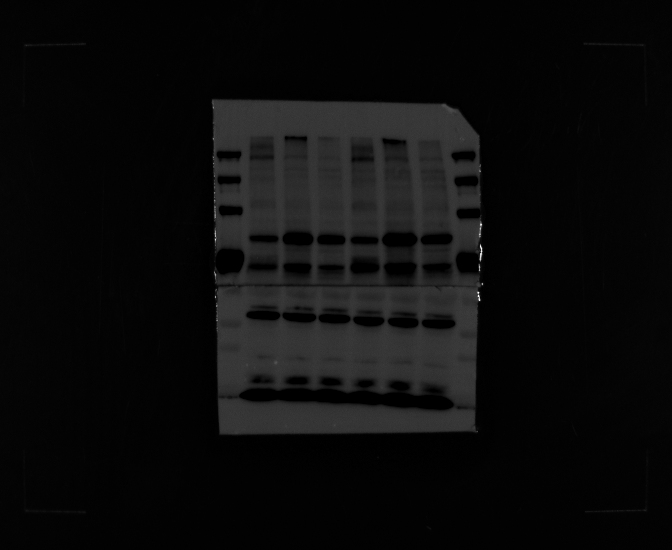

Supplement: Supplementary file 2 [file DataSheet_3.zip › Figures+Tables/Fig.10/Fig.10.(A)/P-PI3K 85kDa/p-pi3k 85kDa+b-actin 42kDa 2.1.jpg]

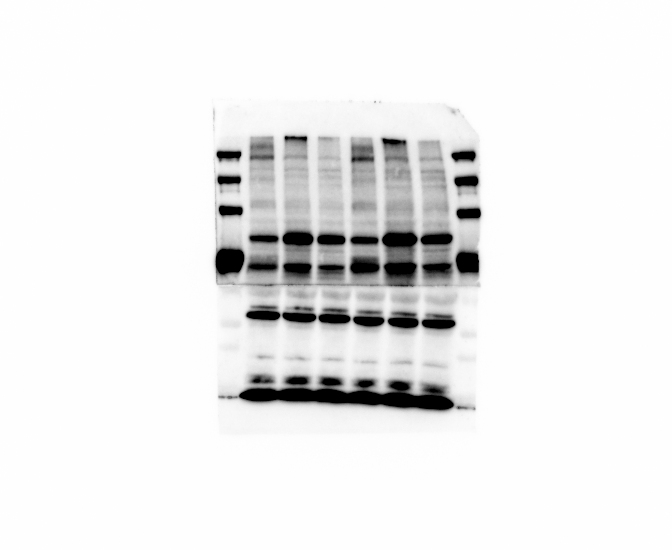

Supplement: Supplementary file 2 [file DataSheet_3.zip › Figures+Tables/Fig.10/Fig.10.(A)/P-PI3K 85kDa/p-pi3k 85kDa+b-actin 42kDa 2.jpg]

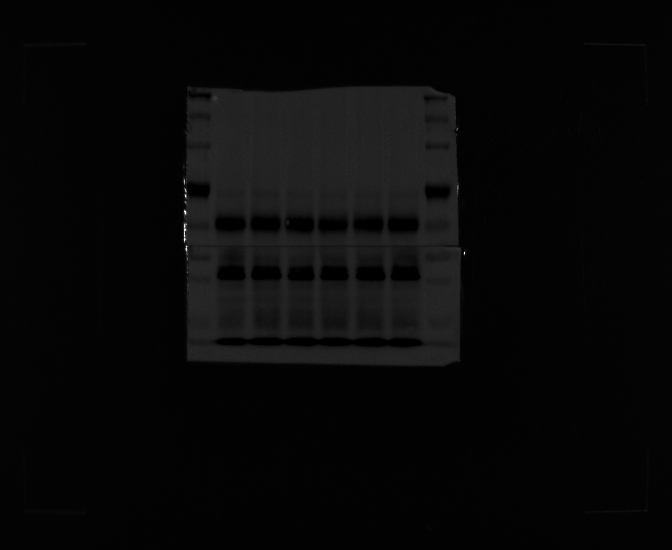

Supplement: Supplementary file 2 [file DataSheet_3.zip › Figures+Tables/Fig.10/Fig.10.(A)/P65 65kDa/P65 65kDa+GAPDH 35kDa 2.1.jpg]

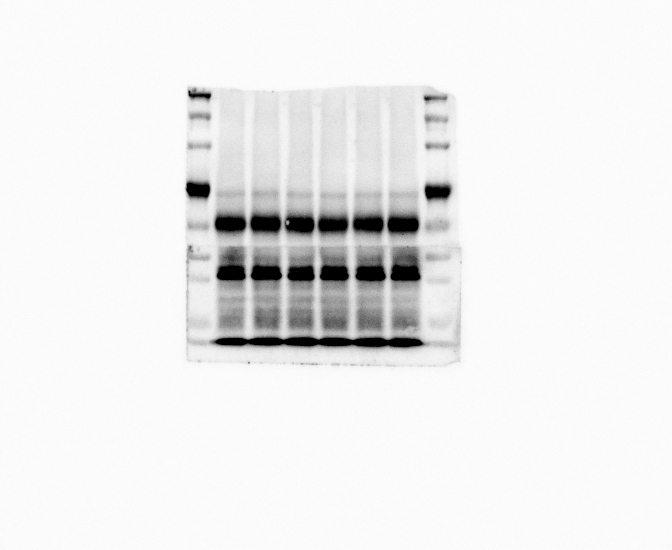

Supplement: Supplementary file 2 [file DataSheet_3.zip › Figures+Tables/Fig.10/Fig.10.(A)/P65 65kDa/P65 65kDa+GAPDH 35kDa 2.jpg]

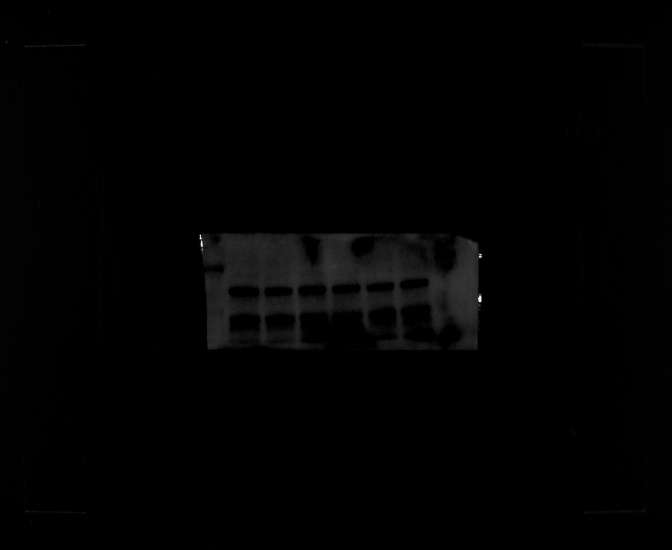

Supplement: Supplementary file 2 [file DataSheet_3.zip › Figures+Tables/Fig.10/Fig.10.(A)/PI3K 85kDa/pi3k 85kDa 1.1.jpg]

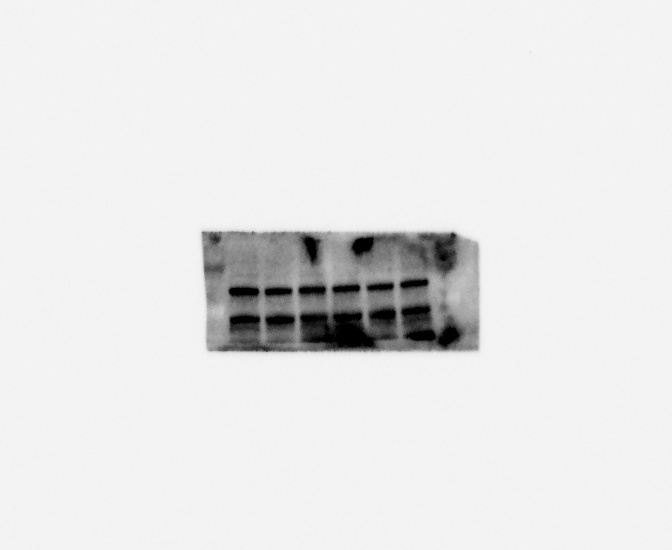

Supplement: Supplementary file 2 [file DataSheet_3.zip › Figures+Tables/Fig.10/Fig.10.(A)/PI3K 85kDa/pi3k 85kDa 1.jpg]

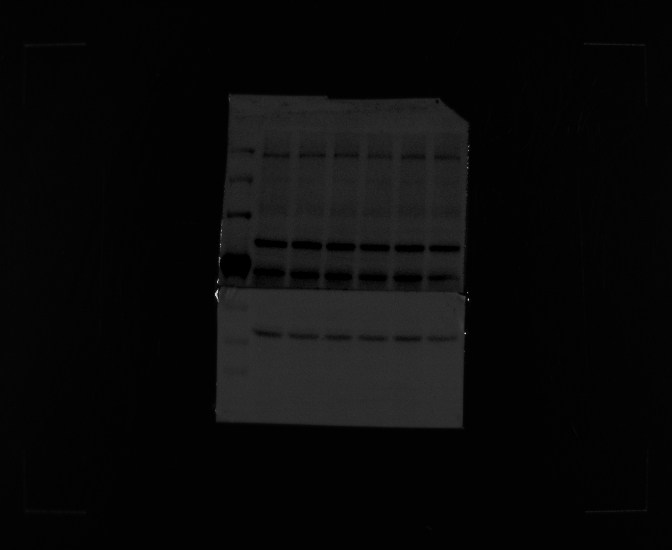

Supplement: Supplementary file 2 [file DataSheet_3.zip › Figures+Tables/Fig.10/Fig.10.(A)/PI3K 85kDa/pi3k 85kDa+b-actin 42kDa 2.1.jpg]

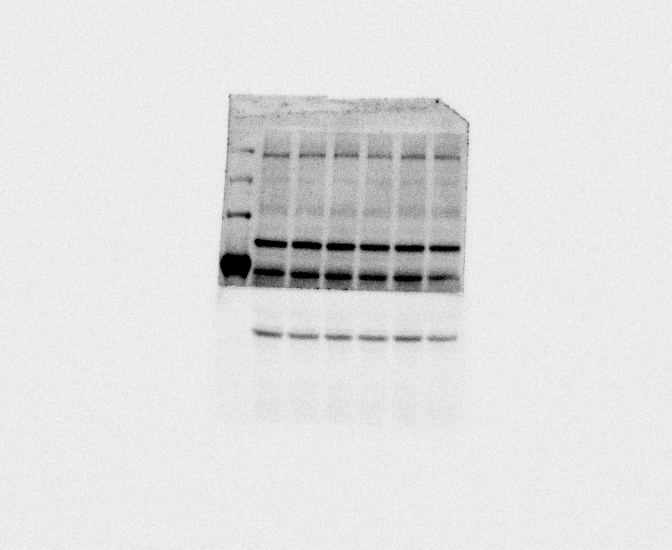

Supplement: Supplementary file 2 [file DataSheet_3.zip › Figures+Tables/Fig.10/Fig.10.(A)/PI3K 85kDa/pi3k 85kDa+b-actin 42kDa 2.jpg]

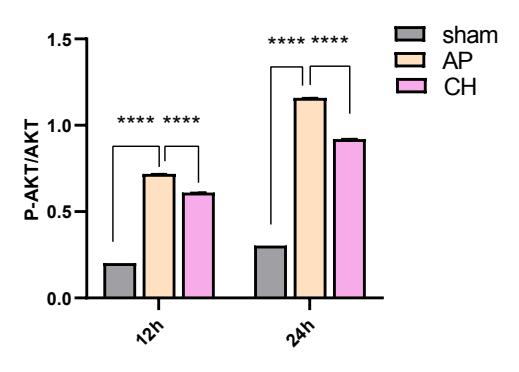

Supplement: Supplementary file 2 [file DataSheet_3.zip › Figures+Tables/Fig.10/Fig.10.(B)/Fig.10.(B)P-AKT_AKT.jpg]

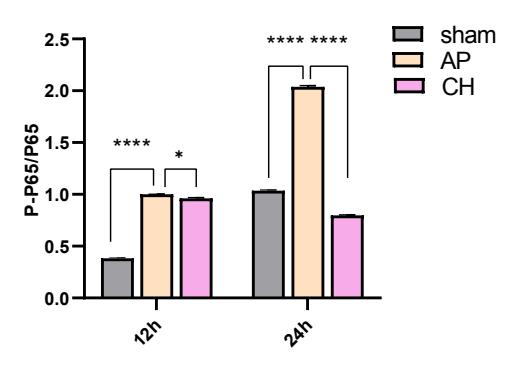

Supplement: Supplementary file 2 [file DataSheet_3.zip › Figures+Tables/Fig.10/Fig.10.(B)/Fig.10.(B)P-P65_P65.jpg]

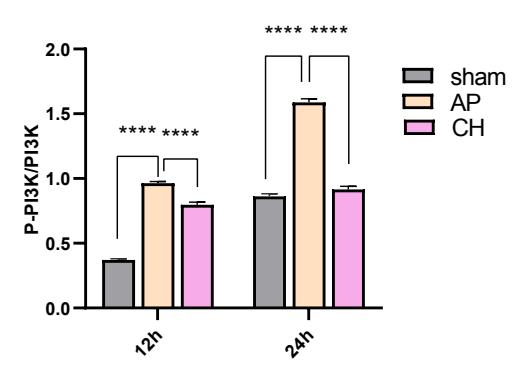

Supplement: Supplementary file 2 [file DataSheet_3.zip › Figures+Tables/Fig.10/Fig.10.(B)/Fig.10.(B)P-PI3K_PI3K.jpg]

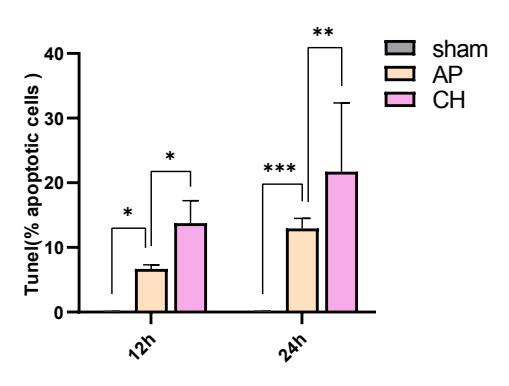

Supplement: Supplementary file 2 [file DataSheet_3.zip › Figures+Tables/Fig.11/Fig.11.(B)Tunel(% apoptotic cells ).jpg]

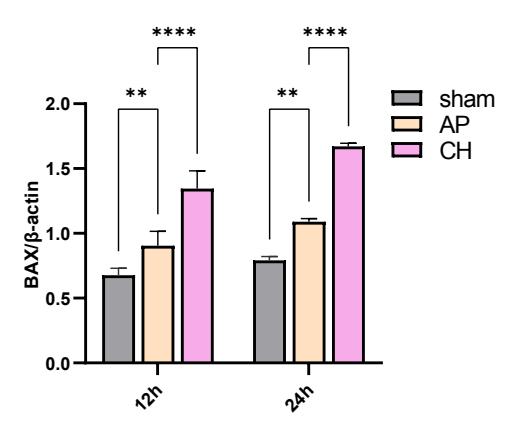

Supplement: Supplementary file 2 [file DataSheet_3.zip › Figures+Tables/Fig.11/Fig.11.(D)BAX_β-actin.jpg]

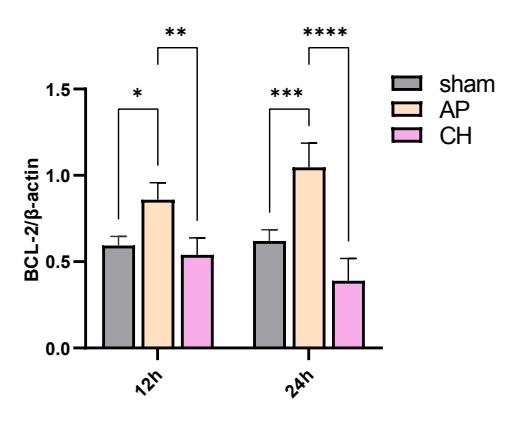

Supplement: Supplementary file 2 [file DataSheet_3.zip › Figures+Tables/Fig.11/Fig.11.(E)BCL-2_β-actin.jpg]

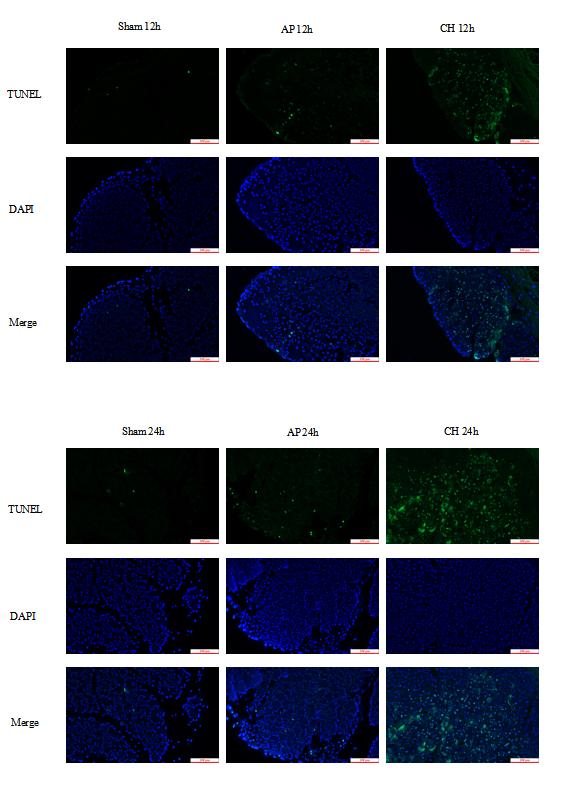

Supplement: Supplementary file 2 [file DataSheet_3.zip › Figures+Tables/Fig.11/Fig.11.(A)Tunel.jpg]

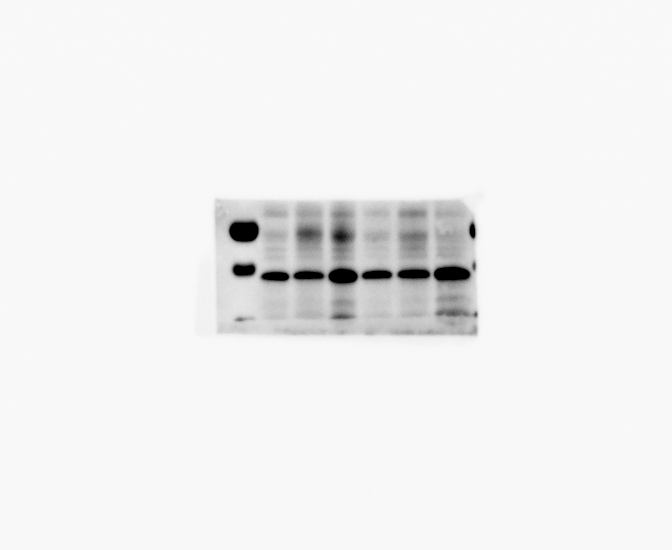

Supplement: Supplementary file 2 [file DataSheet_3.zip › Figures+Tables/Fig.11/Fig.11.(C)/Bax 20kDa/bax 20kDa 1.tif]

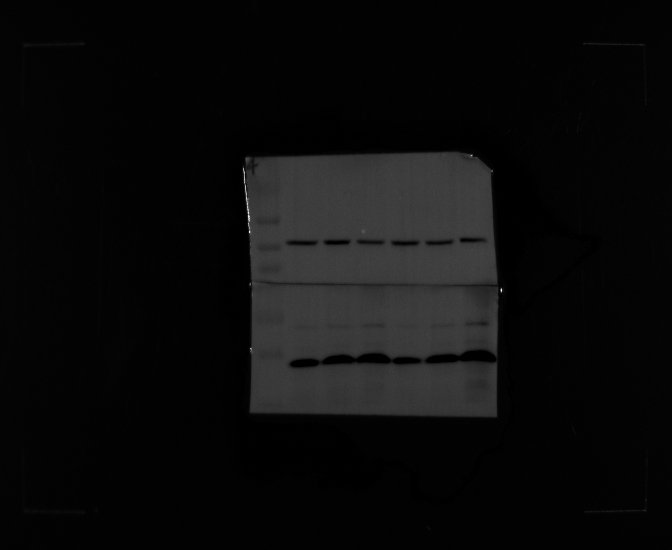

Supplement: Supplementary file 2 [file DataSheet_3.zip › Figures+Tables/Fig.11/Fig.11.(C)/Bax 20kDa/bax 20kDa+b-actin 42kDa 2.1.jpg]

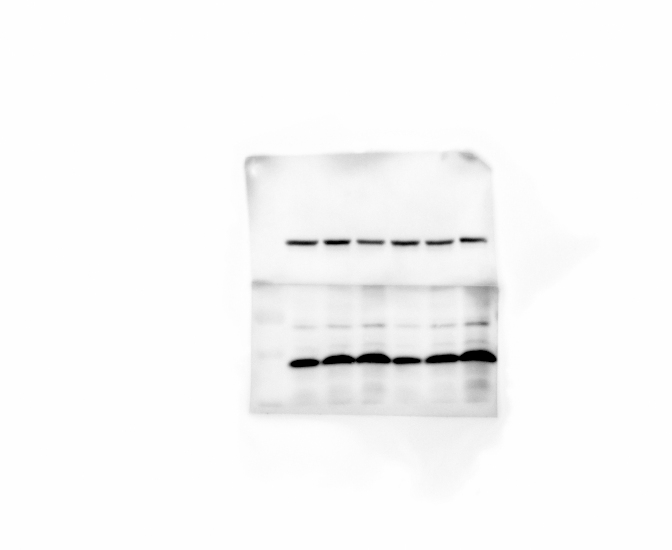

Supplement: Supplementary file 2 [file DataSheet_3.zip › Figures+Tables/Fig.11/Fig.11.(C)/Bax 20kDa/bax 20kDa+b-actin 42kDa 2.jpg]

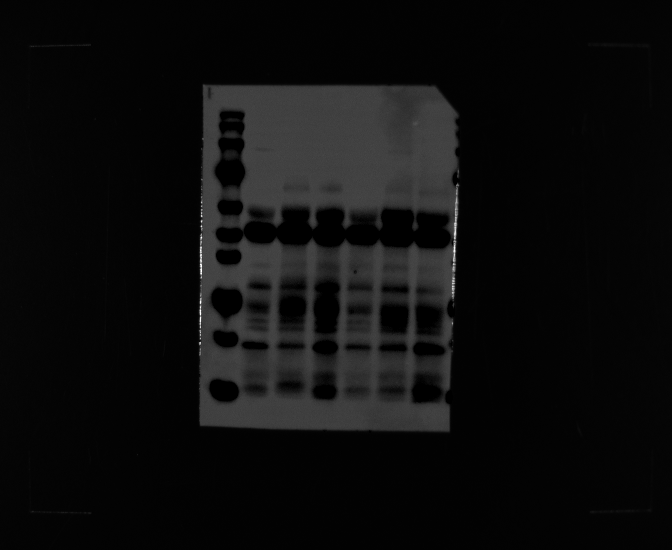

Supplement: Supplementary file 2 [file DataSheet_3.zip › Figures+Tables/Fig.11/Fig.11.(C)/Bax 20kDa/bax 20kDa+b-actin 42kDa 3.1.tif]

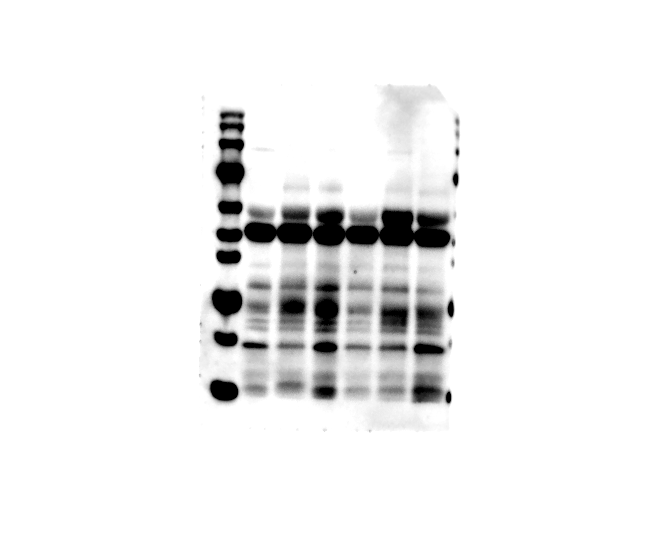

Supplement: Supplementary file 2 [file DataSheet_3.zip › Figures+Tables/Fig.11/Fig.11.(C)/Bax 20kDa/bax 20kDa+b-actin 42kDa 3.tif]

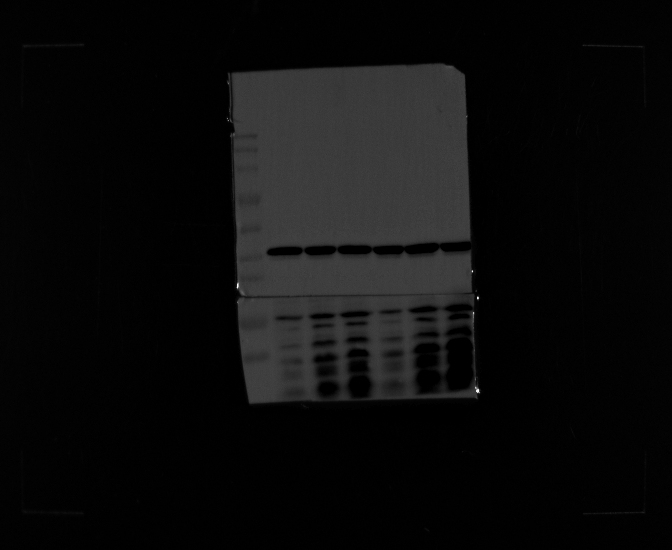

Supplement: Supplementary file 2 [file DataSheet_3.zip › Figures+Tables/Fig.11/Fig.11.(C)/Bax 20kDa/bax 20kDa+b-actin 42kDa 4.1.jpg]

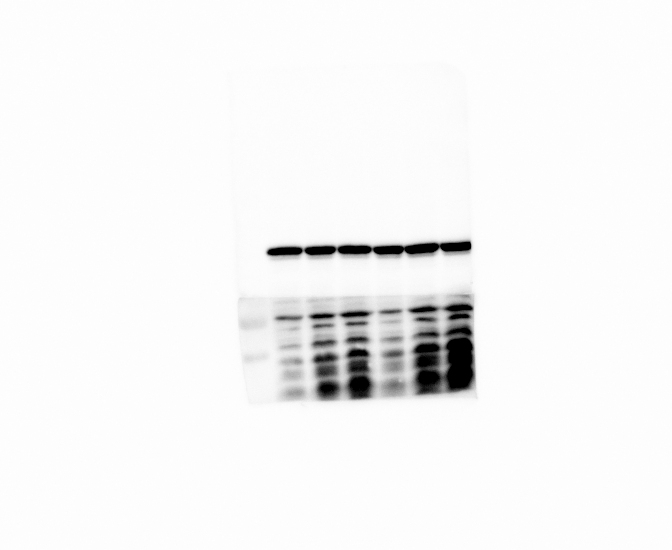

Supplement: Supplementary file 2 [file DataSheet_3.zip › Figures+Tables/Fig.11/Fig.11.(C)/Bax 20kDa/bax 20kDa+b-actin 42kDa 4.jpg]

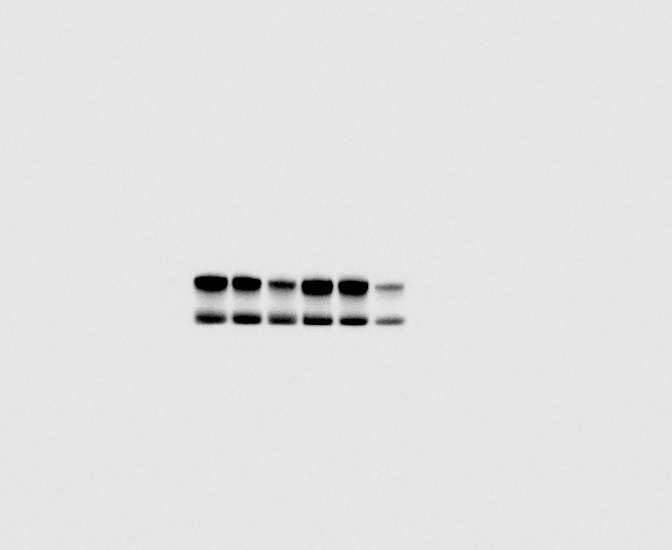

Supplement: Supplementary file 2 [file DataSheet_3.zip › Figures+Tables/Fig.11/Fig.11.(C)/Bcl-2 26kDa/bcl-2 26kDa 1.jpg]

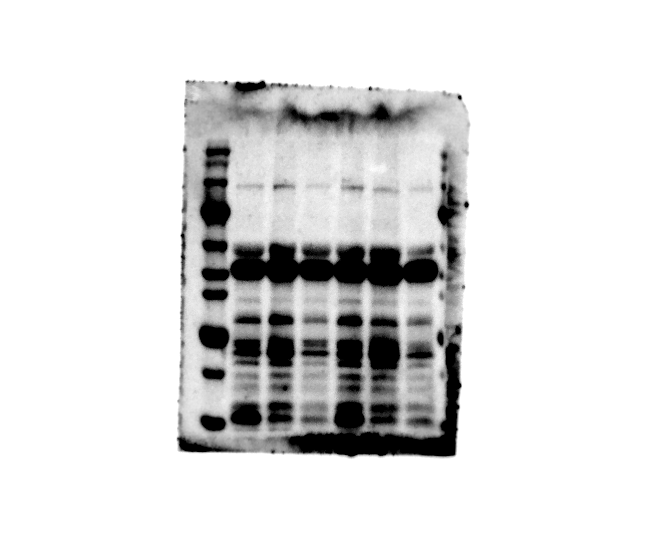

Supplement: Supplementary file 2 [file DataSheet_3.zip › Figures+Tables/Fig.11/Fig.11.(C)/Bcl-2 26kDa/bcl-2 26kDa+b-actin 42kDa 2.1.tif]

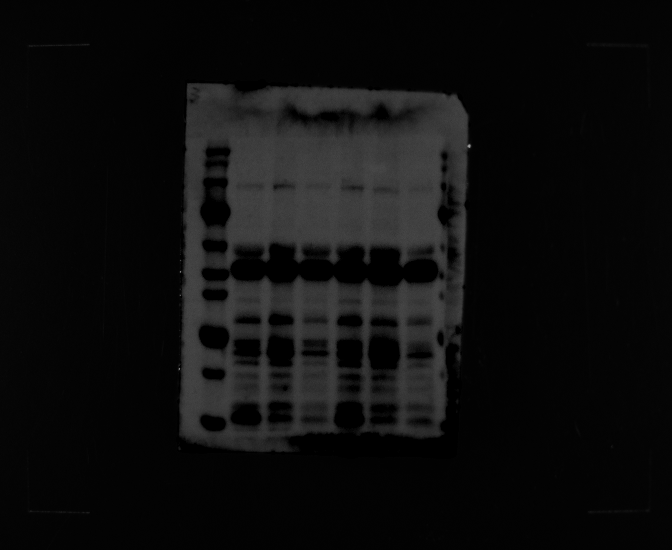

Supplement: Supplementary file 2 [file DataSheet_3.zip › Figures+Tables/Fig.11/Fig.11.(C)/Bcl-2 26kDa/bcl-2 26kDa+b-actin 42kDa 2.tif]

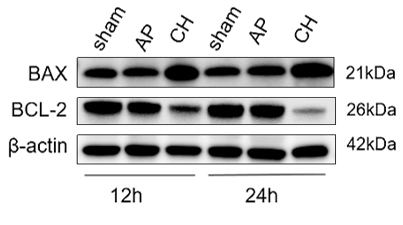

Supplement: Supplementary file 2 [file DataSheet_3.zip › Figures+Tables/Fig.11/Fig.11.(C)/Fig.11.(C).jpg]

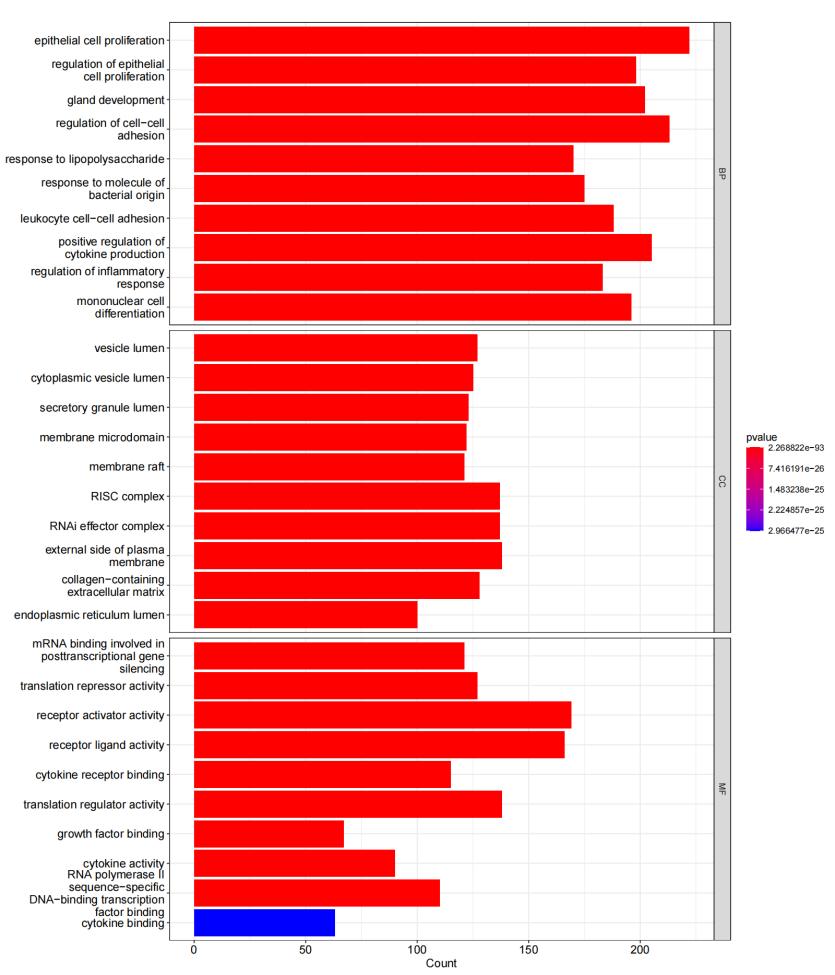

Supplement: Supplementary file 2 [file DataSheet_3.zip › Figures+Tables/Fig.3/Fig.3.(A)KEGG/Fig.3.(A)KEGG.jpg]

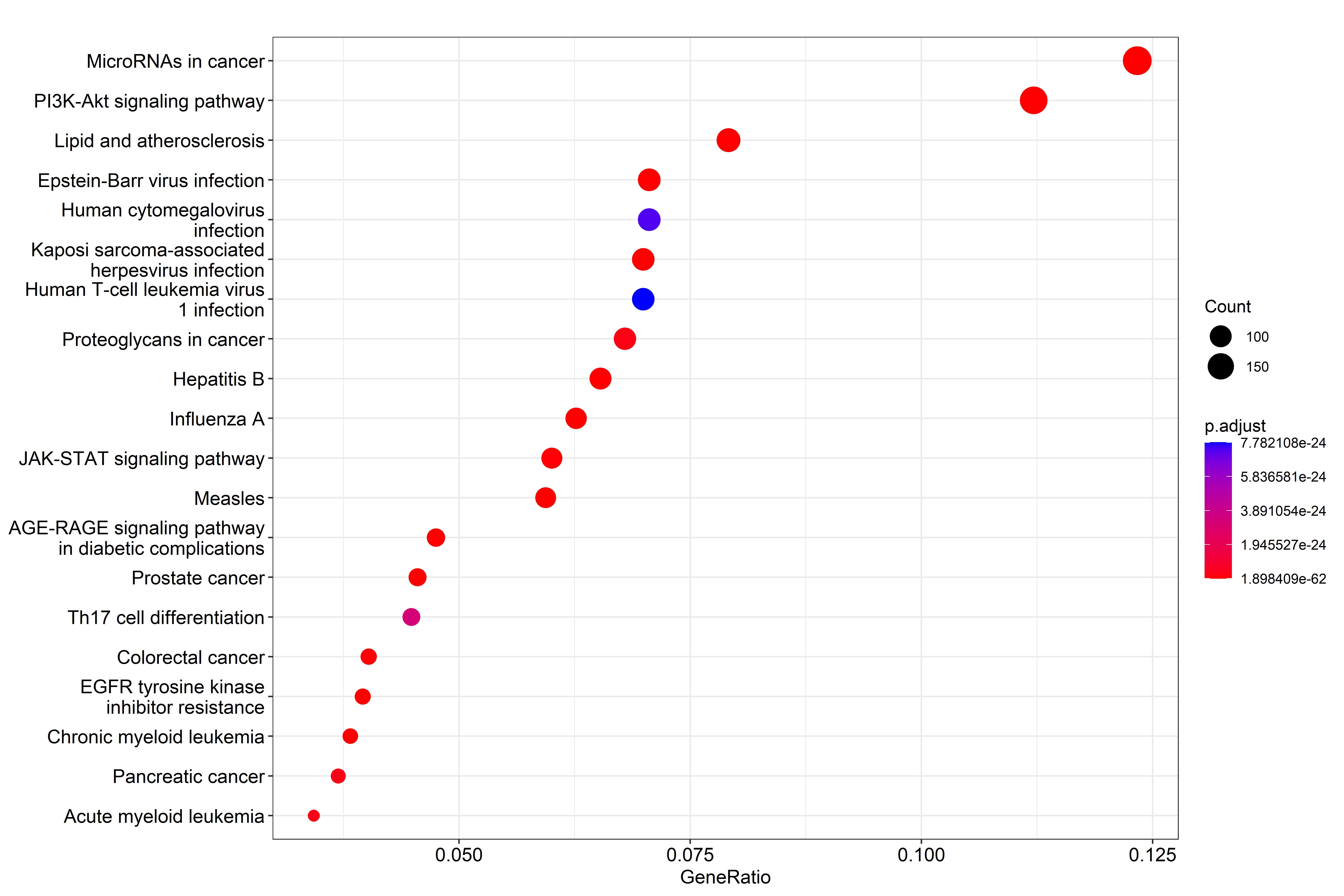

Supplement: Supplementary file 2 [file DataSheet_3.zip › Figures+Tables/Fig.3/Fig.3.(B)GO/Fig.3.(B)GO.jpg]

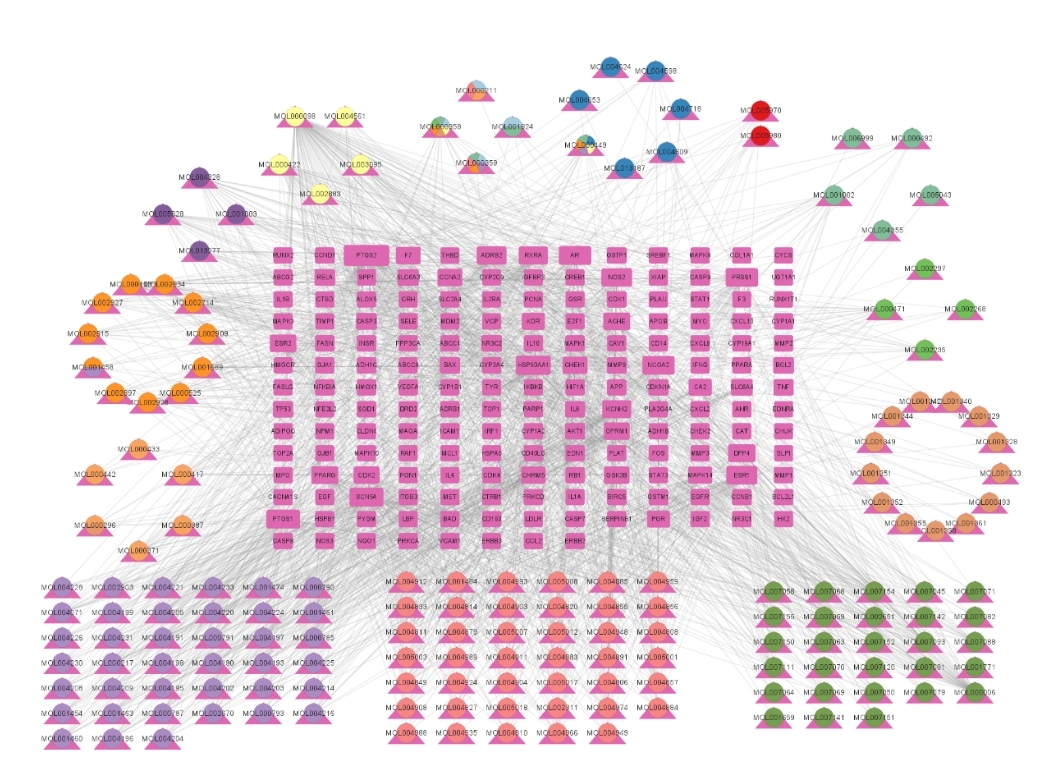

Supplement: Supplementary file 2 [file DataSheet_3.zip › Figures+Tables/Fig.4.jpg]

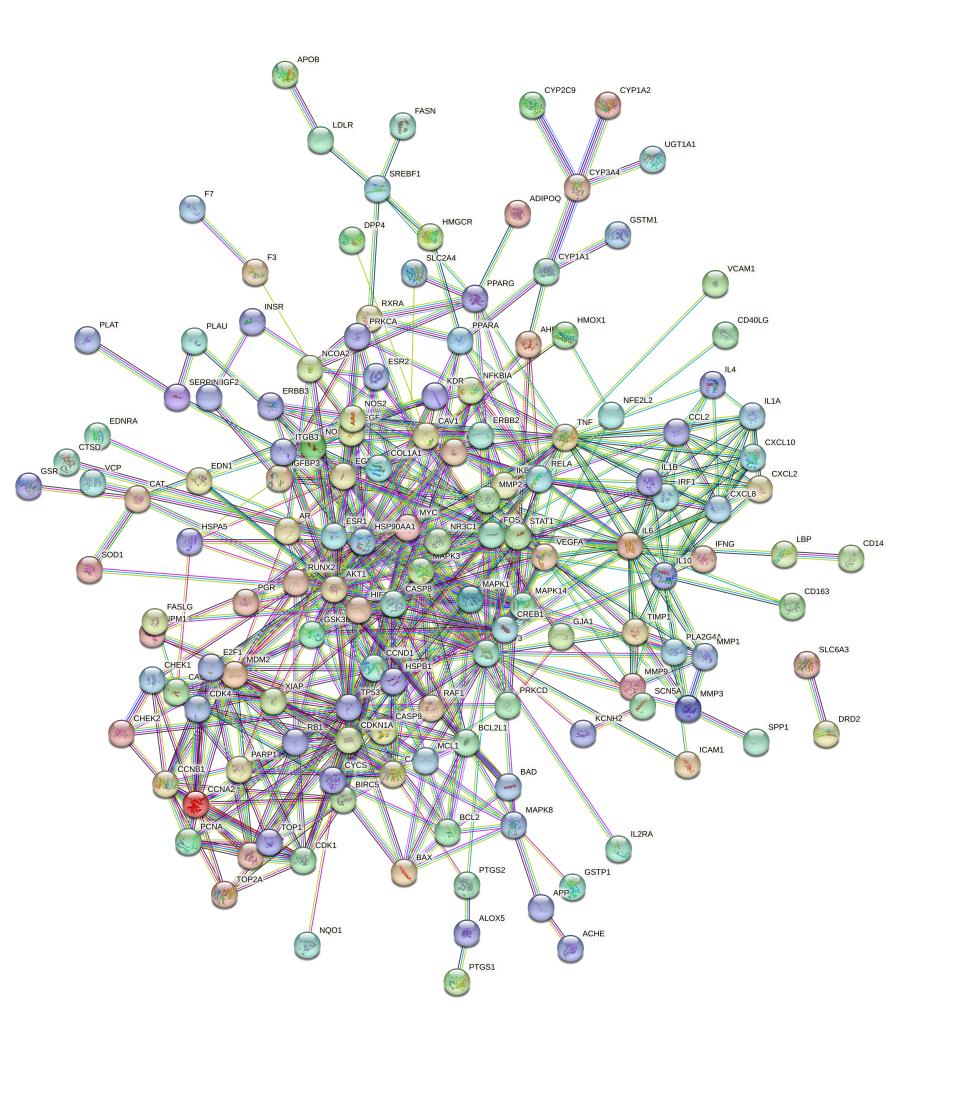

Supplement: Supplementary file 2 [file DataSheet_3.zip › Figures+Tables/Fig.5/Fig.5.(A).jpg]

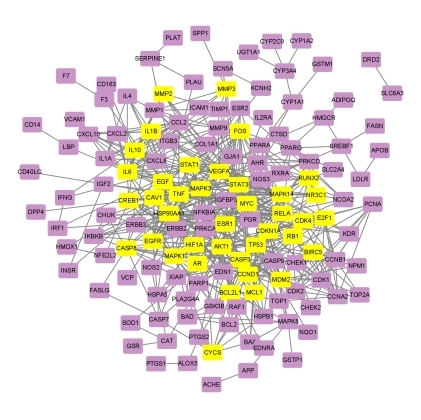

Supplement: Supplementary file 2 [file DataSheet_3.zip › Figures+Tables/Fig.5/Fig.5.(B1)/Fig.5.(B1).jpg]

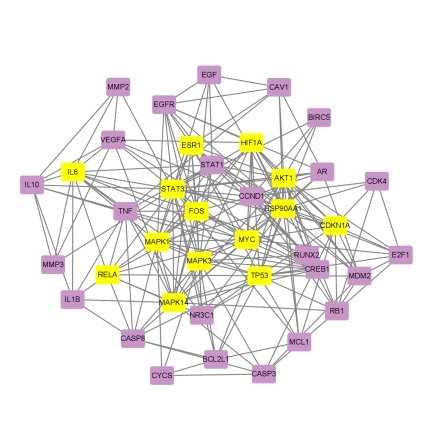

Supplement: Supplementary file 2 [file DataSheet_3.zip › Figures+Tables/Fig.5/Fig.5.(B2)/Fig.5.(B2).jpg]

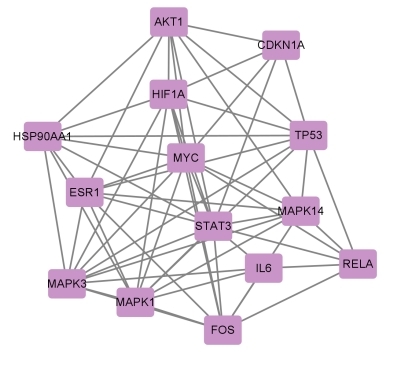

Supplement: Supplementary file 2 [file DataSheet_3.zip › Figures+Tables/Fig.5/Fig.5.(B3)/Fig.5.(B3).jpg]

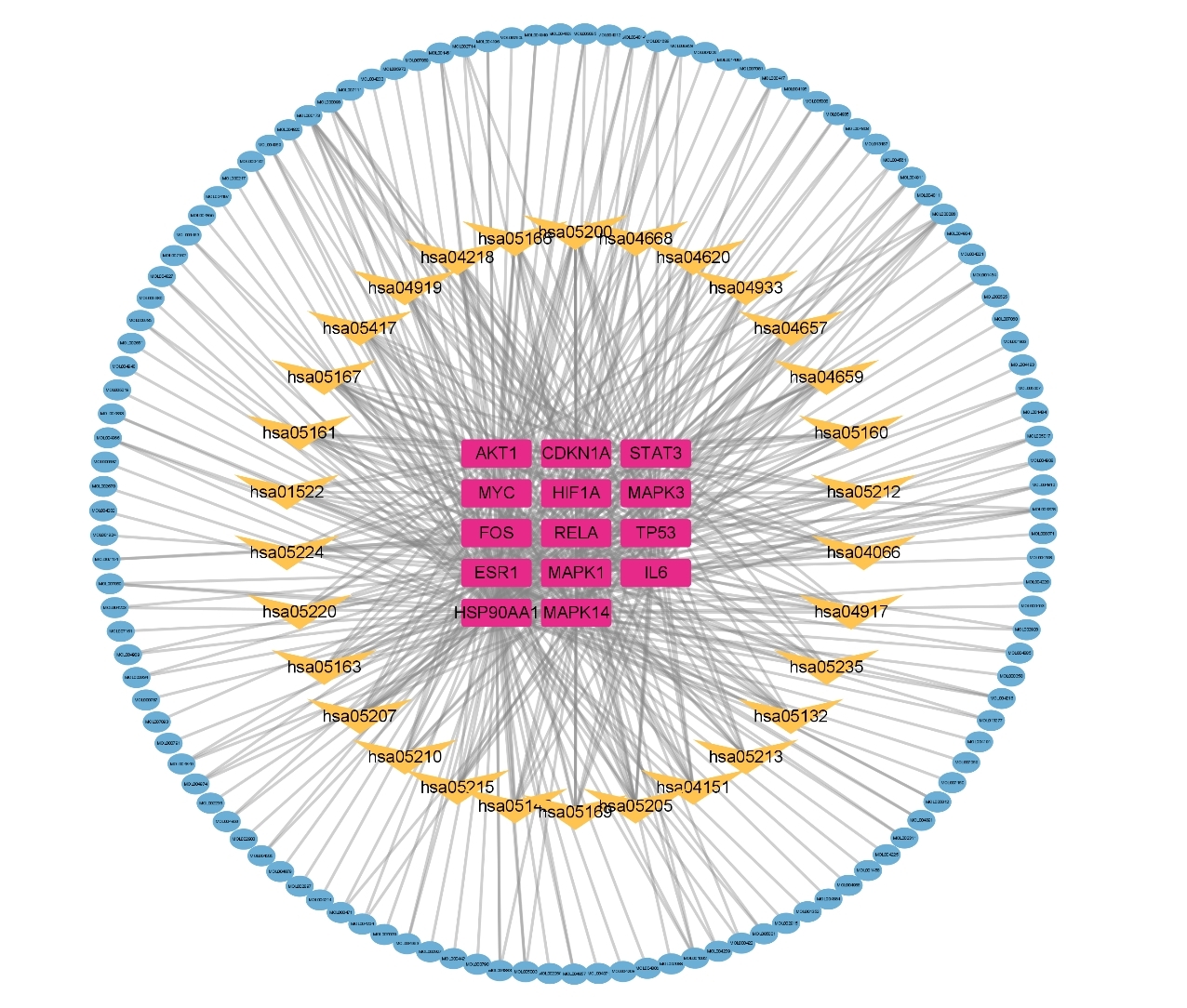

Supplement: Supplementary file 2 [file DataSheet_3.zip › Figures+Tables/Fig.6.jpg]

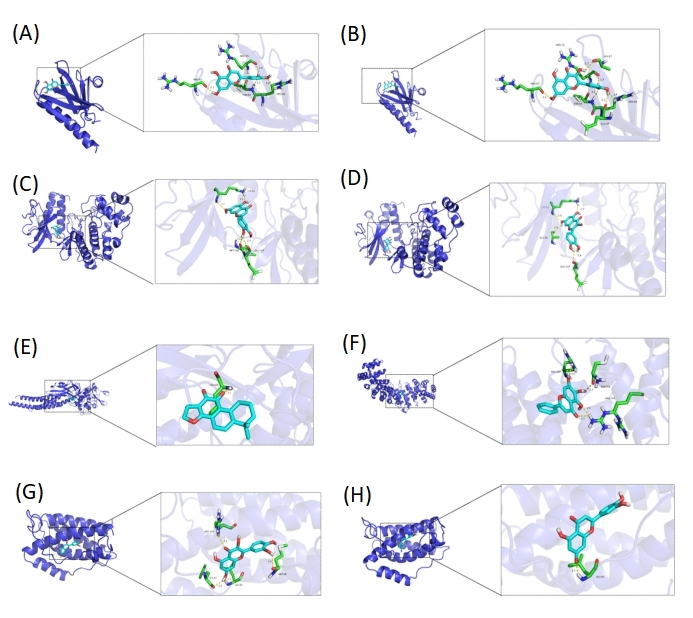

Supplement: Supplementary file 2 [file DataSheet_3.zip › Figures+Tables/Fig.7/Fig.7.jpg]

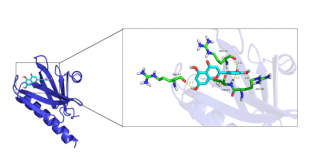

Supplement: Supplementary file 2 [file DataSheet_3.zip › Figures+Tables/Fig.7/(A)Luteolin-AKT1.png]

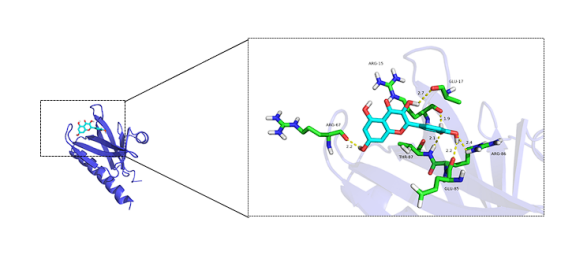

Supplement: Supplementary file 2 [file DataSheet_3.zip › Figures+Tables/Fig.7/(B)Quercetin-AKT1.png]

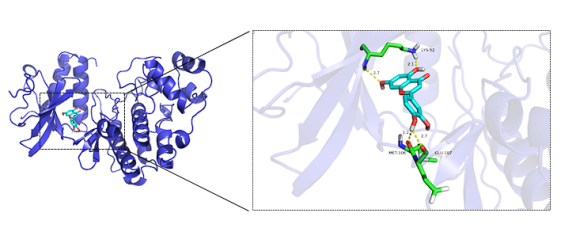

Supplement: Supplementary file 2 [file DataSheet_3.zip › Figures+Tables/Fig.7/(C)Luteolin-MAPK1.png]

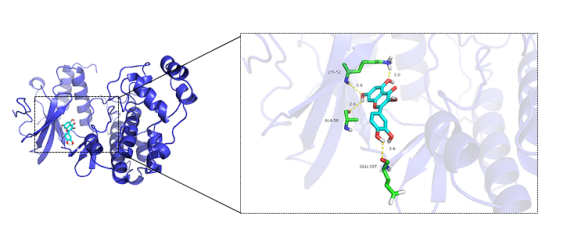

Supplement: Supplementary file 2 [file DataSheet_3.zip › Figures+Tables/Fig.7/(D)Quercetin-MAPK1.png]

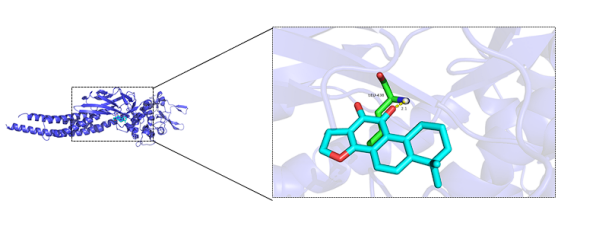

Supplement: Supplementary file 2 [file DataSheet_3.zip › Figures+Tables/Fig.7/(E)Cryptotanshinone-STAT3.png]

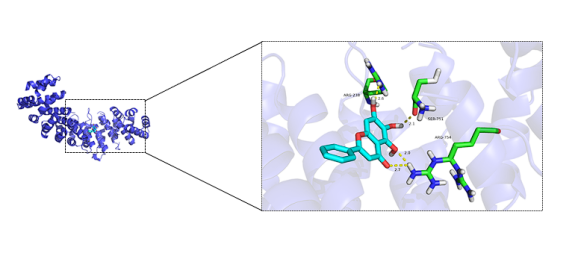

Supplement: Supplementary file 2 [file DataSheet_3.zip › Figures+Tables/Fig.7/(F)Baicalein-HIF-1α.png]

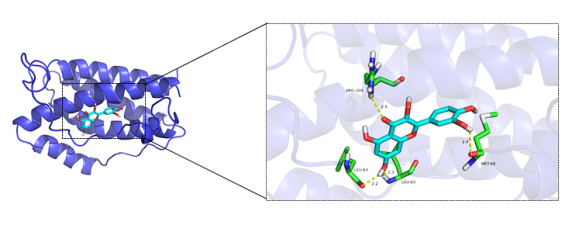

Supplement: Supplementary file 2 [file DataSheet_3.zip › Figures+Tables/Fig.7/(G)Quercetin-IL6.png]

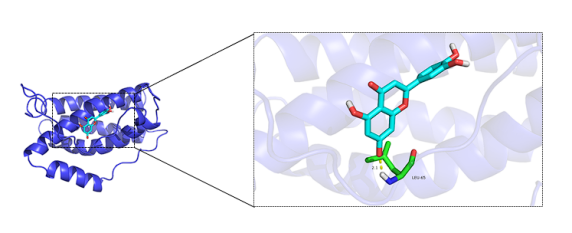

Supplement: Supplementary file 2 [file DataSheet_3.zip › Figures+Tables/Fig.7/(H)Luteolin-IL6.png]

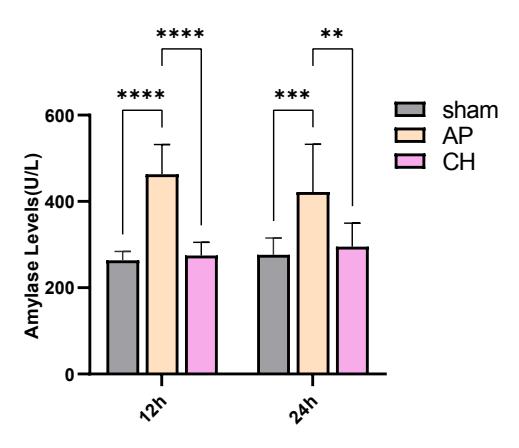

Supplement: Supplementary file 2 [file DataSheet_3.zip › Figures+Tables/Fig.8/Fig.8.(A)/Fig.8.(A)Amylase Levels(U_L).jpg]

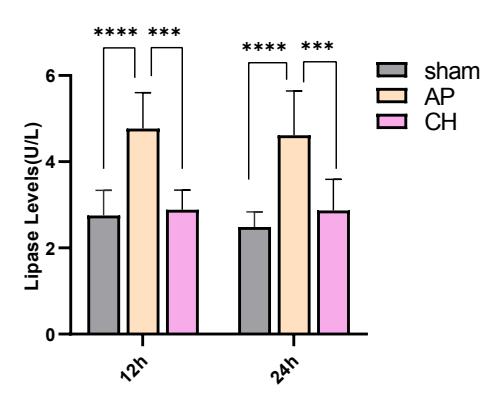

Supplement: Supplementary file 2 [file DataSheet_3.zip › Figures+Tables/Fig.8/Fig.8.(A)/Fig.8.(A)Lipase Levels(U_L).jpg]

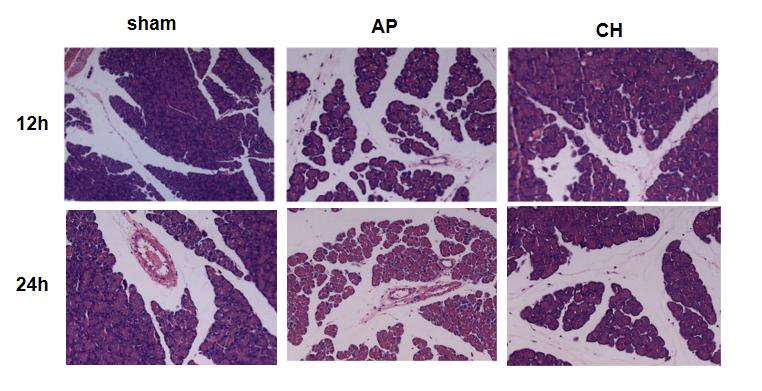

Supplement: Supplementary file 2 [file DataSheet_3.zip › Figures+Tables/Fig.8/Fig.8.(B).jpg]

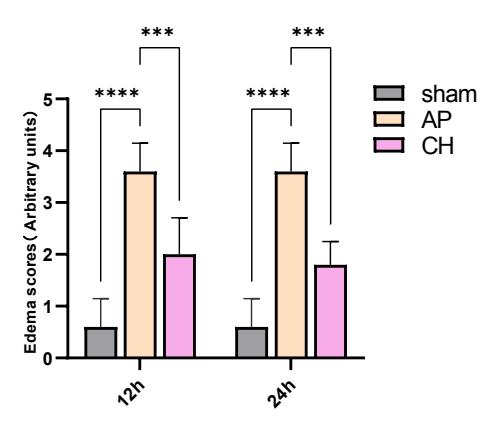

Supplement: Supplementary file 2 [file DataSheet_3.zip › Figures+Tables/Fig.8/Fig.8.(C)/Fig.8.(C)Edema scores(Arbitrary units).jpg]

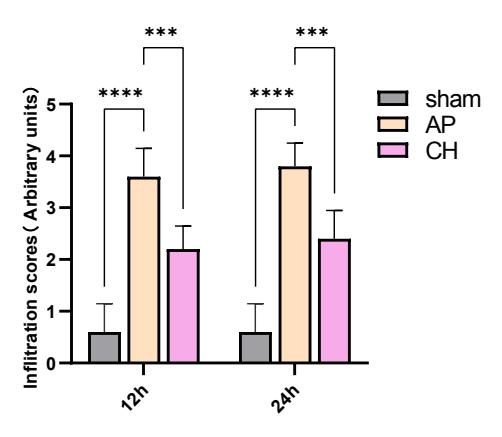

Supplement: Supplementary file 2 [file DataSheet_3.zip › Figures+Tables/Fig.8/Fig.8.(C)/Fig.8.(C)Inflitration scores(Arbitrary units).jpg]

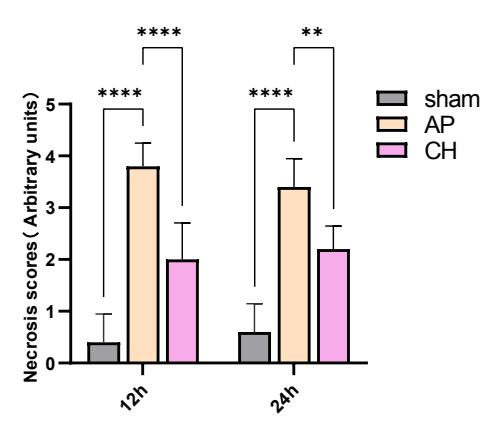

Supplement: Supplementary file 2 [file DataSheet_3.zip › Figures+Tables/Fig.8/Fig.8.(C)/Fig.8.(C)Necrosis scores(Arbitrary units).jpg]

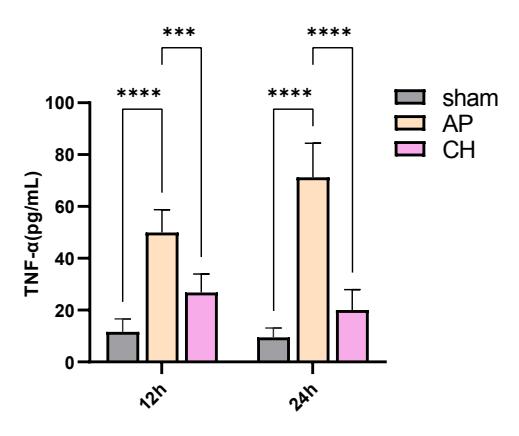

Supplement: Supplementary file 2 [file DataSheet_3.zip › Figures+Tables/Fig.9/Fig.9.(A)TNF-a/Fig.9.(A)TNF-α(pg_mL).jpg]

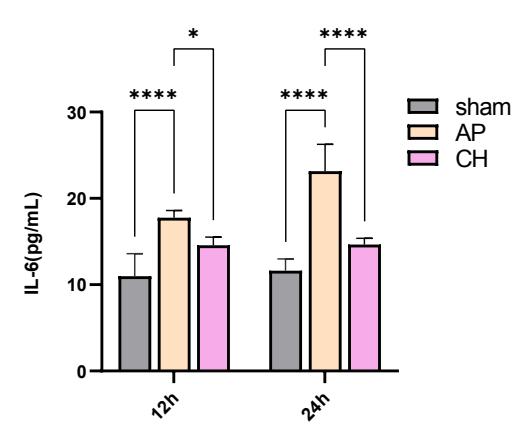

Supplement: Supplementary file 2 [file DataSheet_3.zip › Figures+Tables/Fig.9/Fig.9.(B)IL-6/Fig.9.(B)IL-6(pg_mL)(pg_mL).jpg]

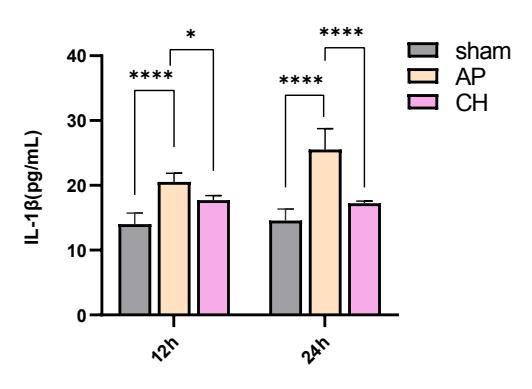

Supplement: Supplementary file 2 [file DataSheet_3.zip › Figures+Tables/Fig.9/Fig.9.(C)IL-1β/Fig.9.(C)IL-1β(pg_mL).jpg]

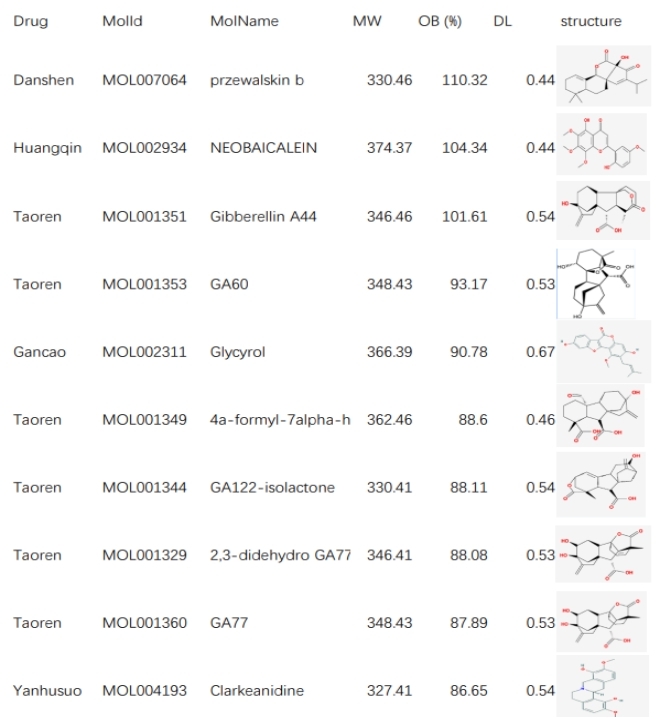

Supplement: Supplementary file 2 [file DataSheet_3.zip › Figures+Tables/Table/Table 1.jpg]

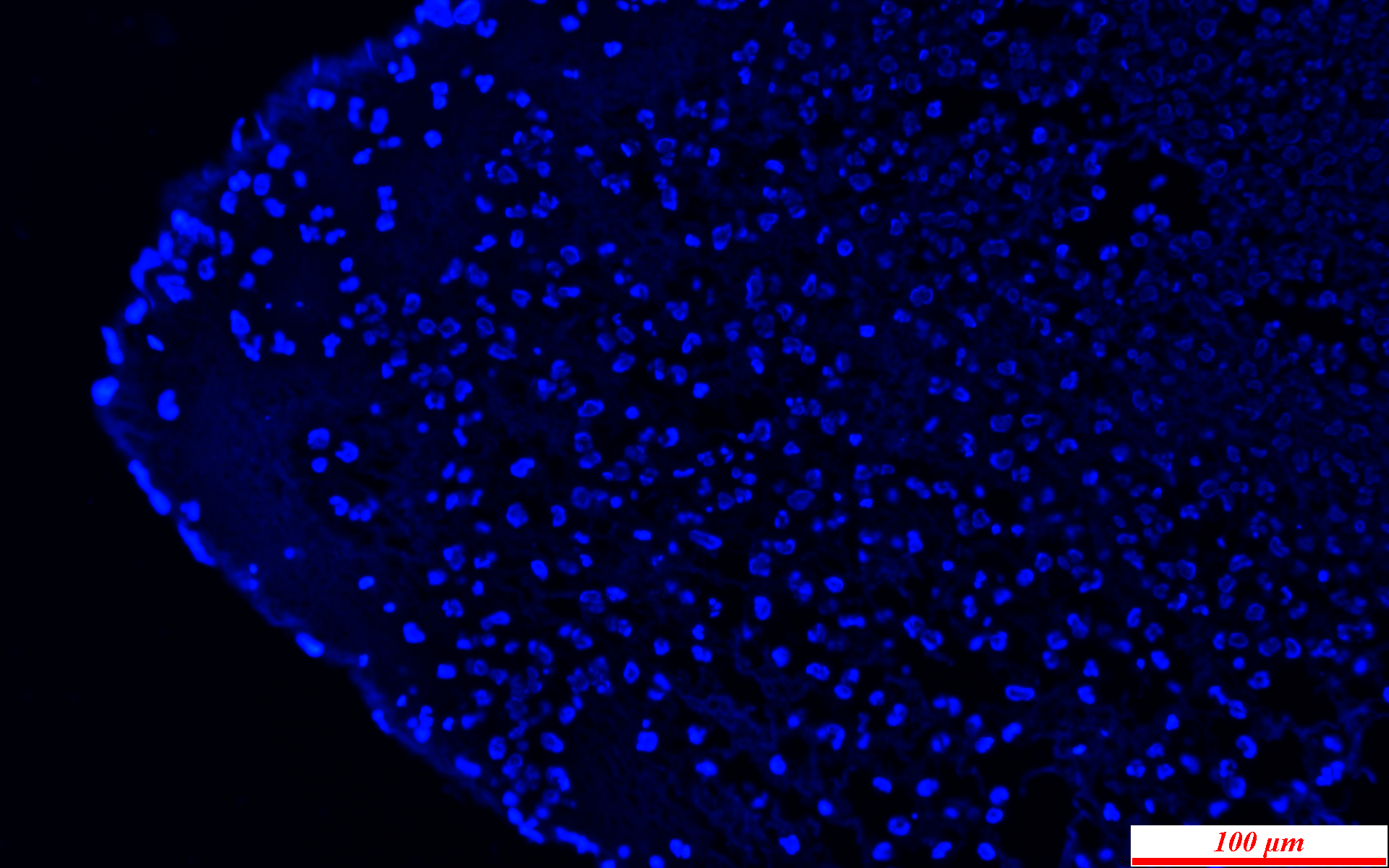

Supplement: Supplementary file 3 [file DataSheet_4.zip › Fig.11microscopy images of AP12h/AP12h DAPI.tif]

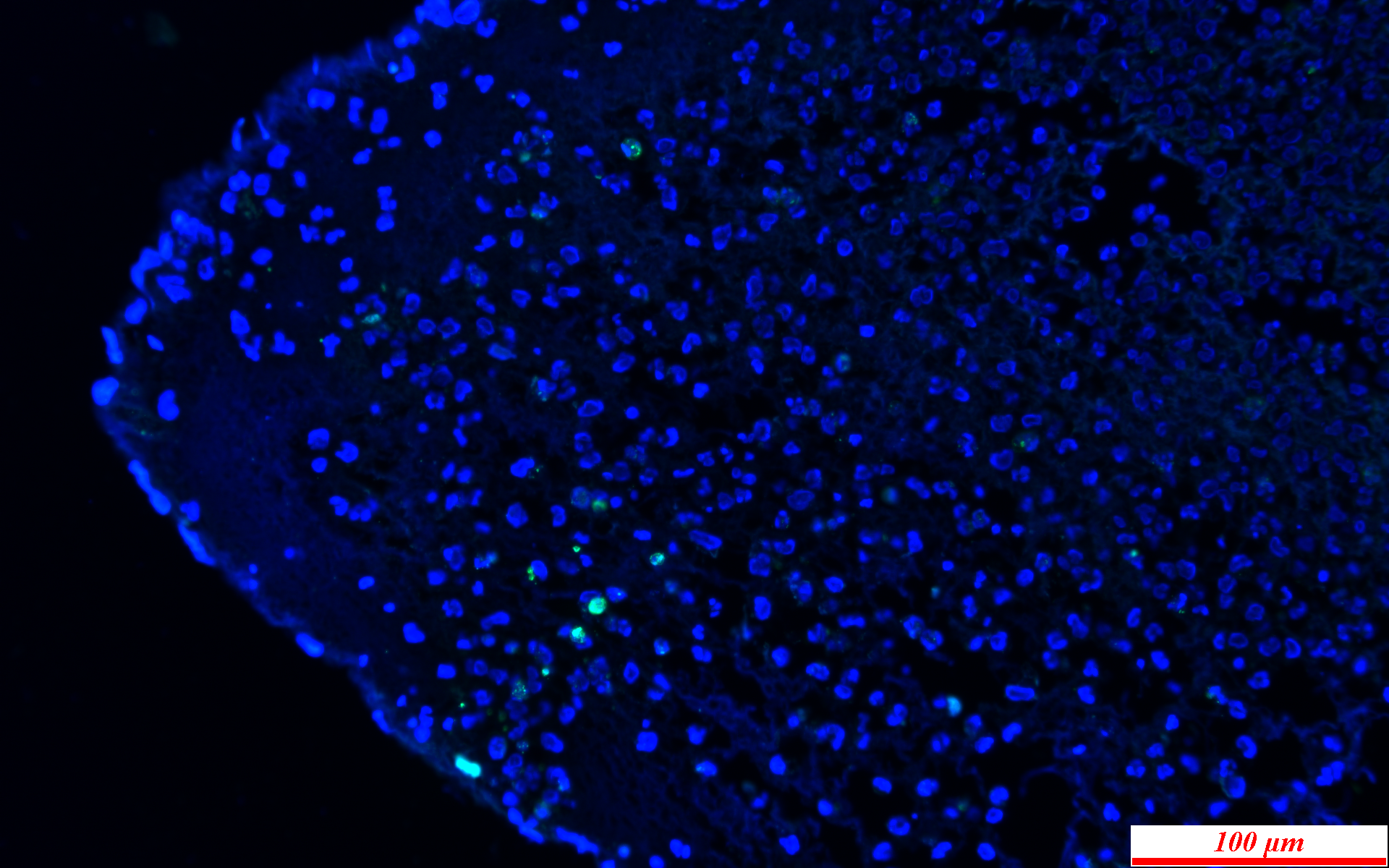

Supplement: Supplementary file 3 [file DataSheet_4.zip › Fig.11microscopy images of AP12h/AP12h Merge.tif]

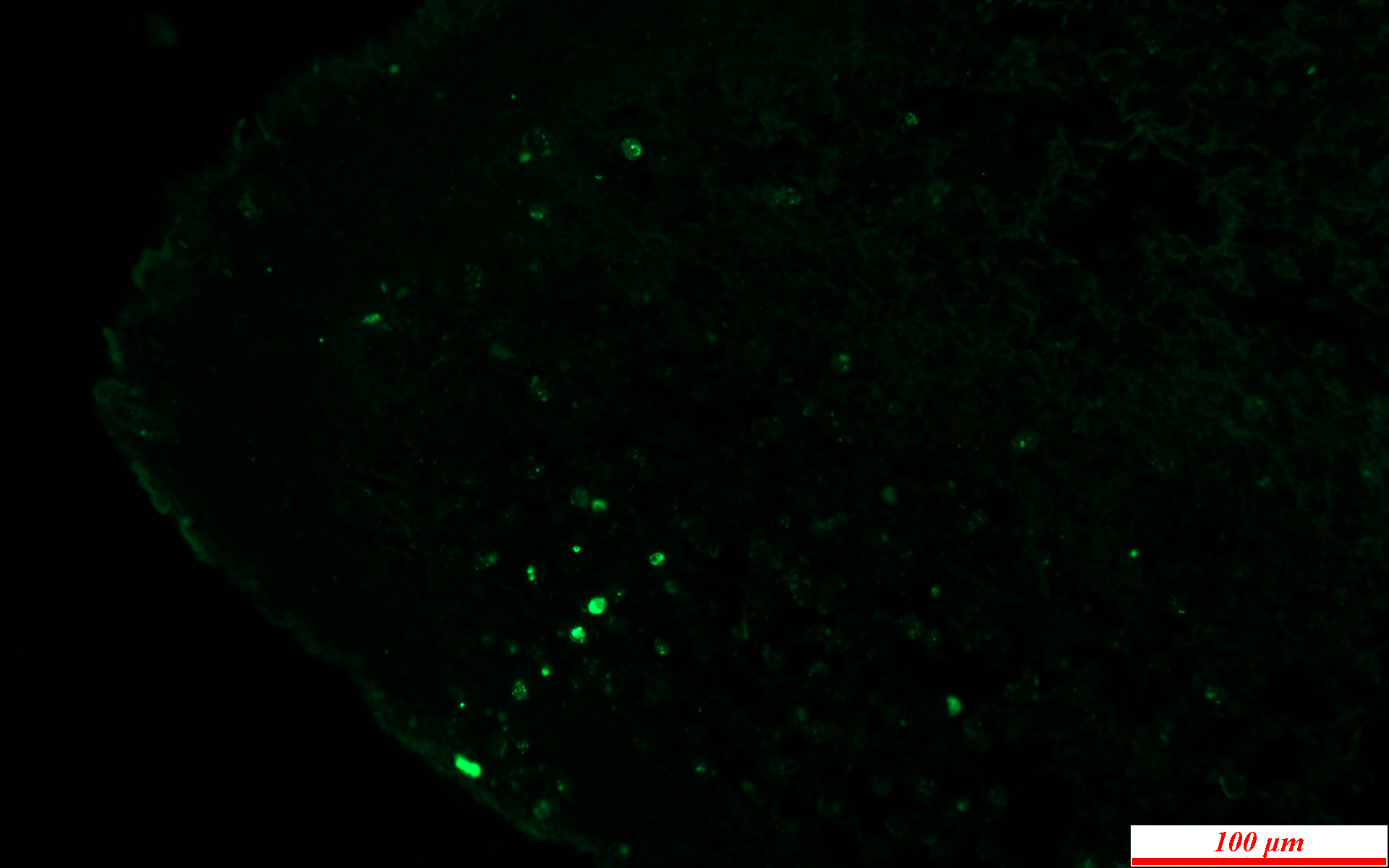

Supplement: Supplementary file 3 [file DataSheet_4.zip › Fig.11microscopy images of AP12h/AP12h Tunel.tif]

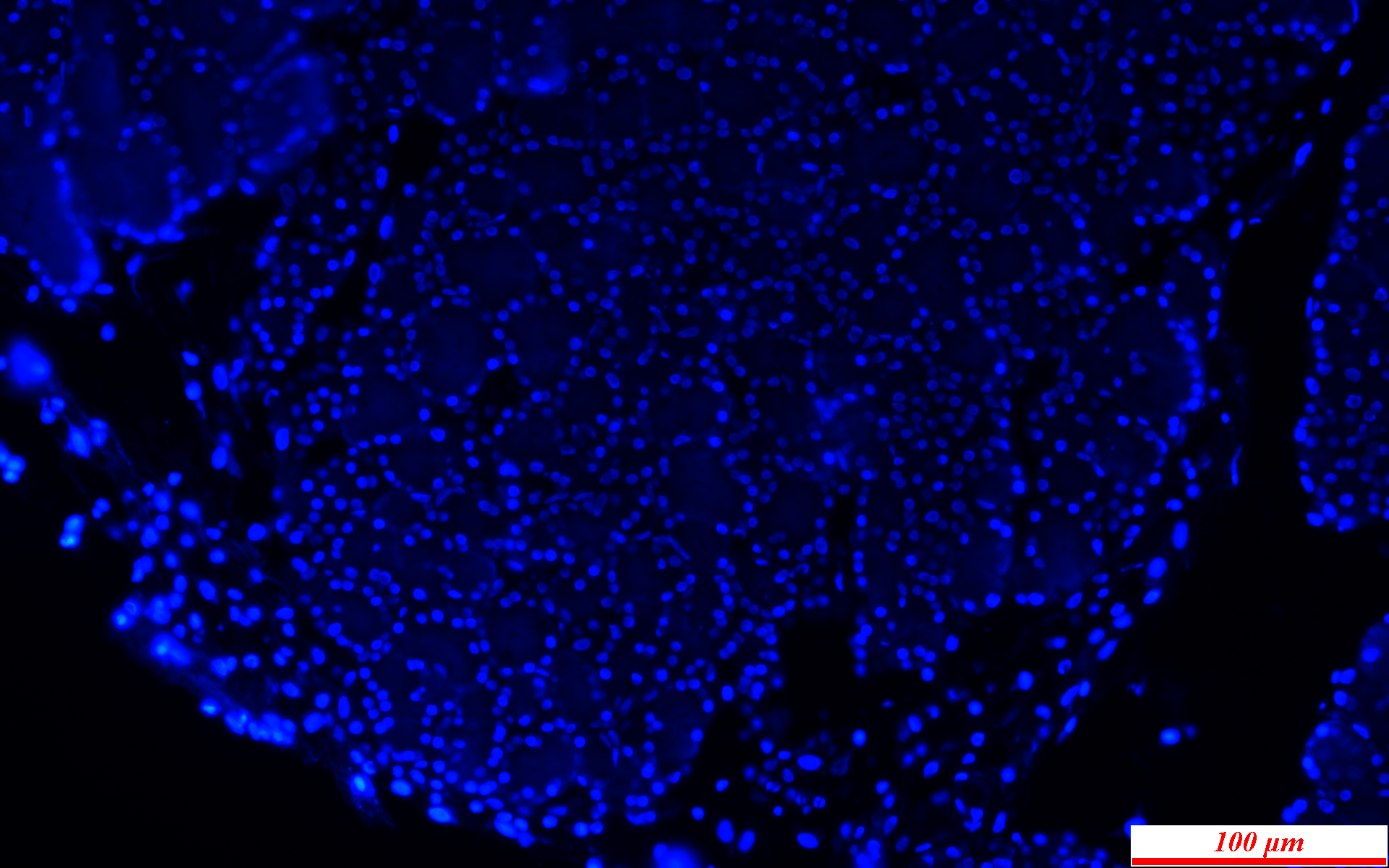

Supplement: Supplementary file 4 [file DataSheet_5.zip › Fig.11microscopy images of AP24h/AP24h DAPI.tif]

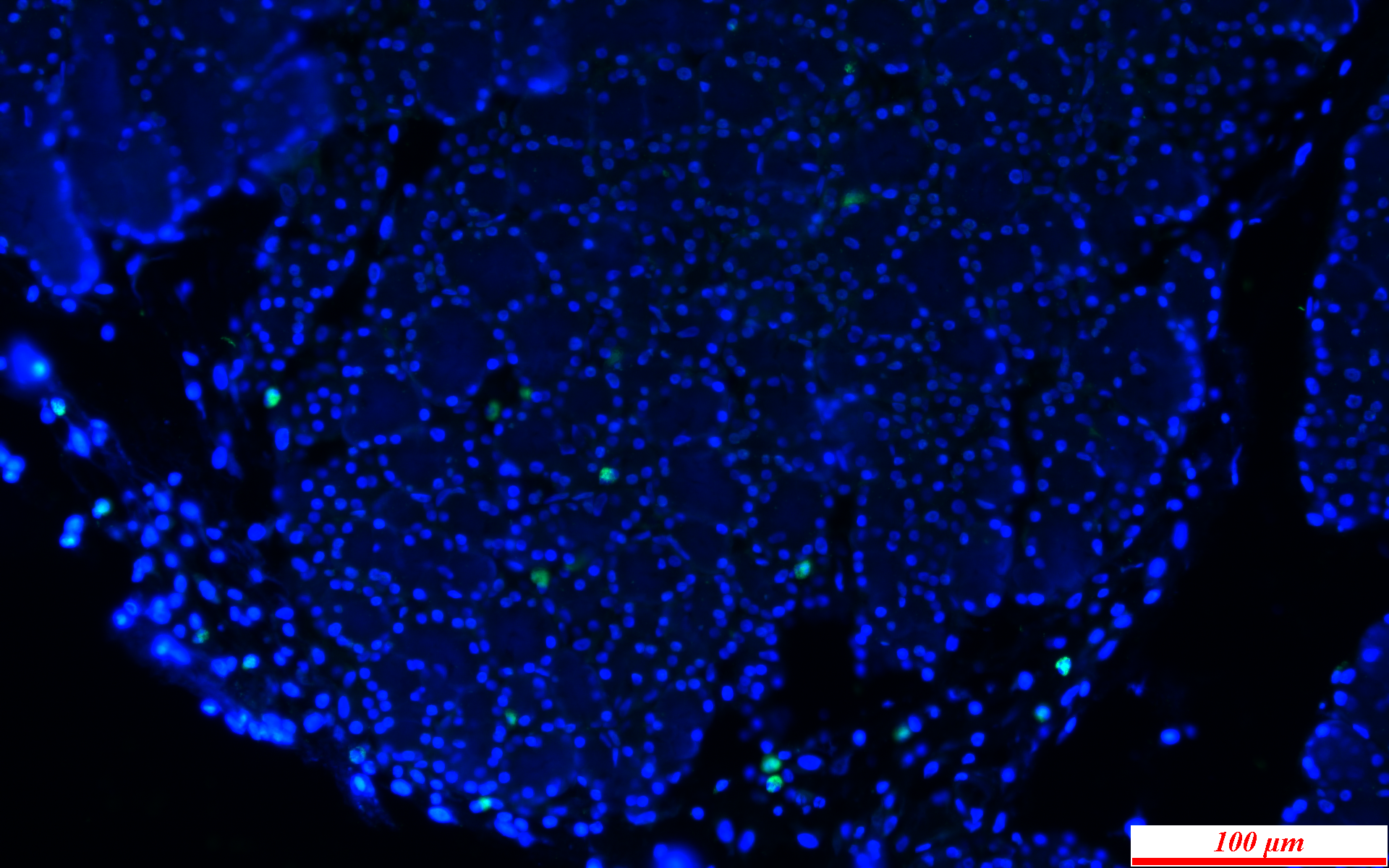

Supplement: Supplementary file 4 [file DataSheet_5.zip › Fig.11microscopy images of AP24h/AP24h Merge.tif]

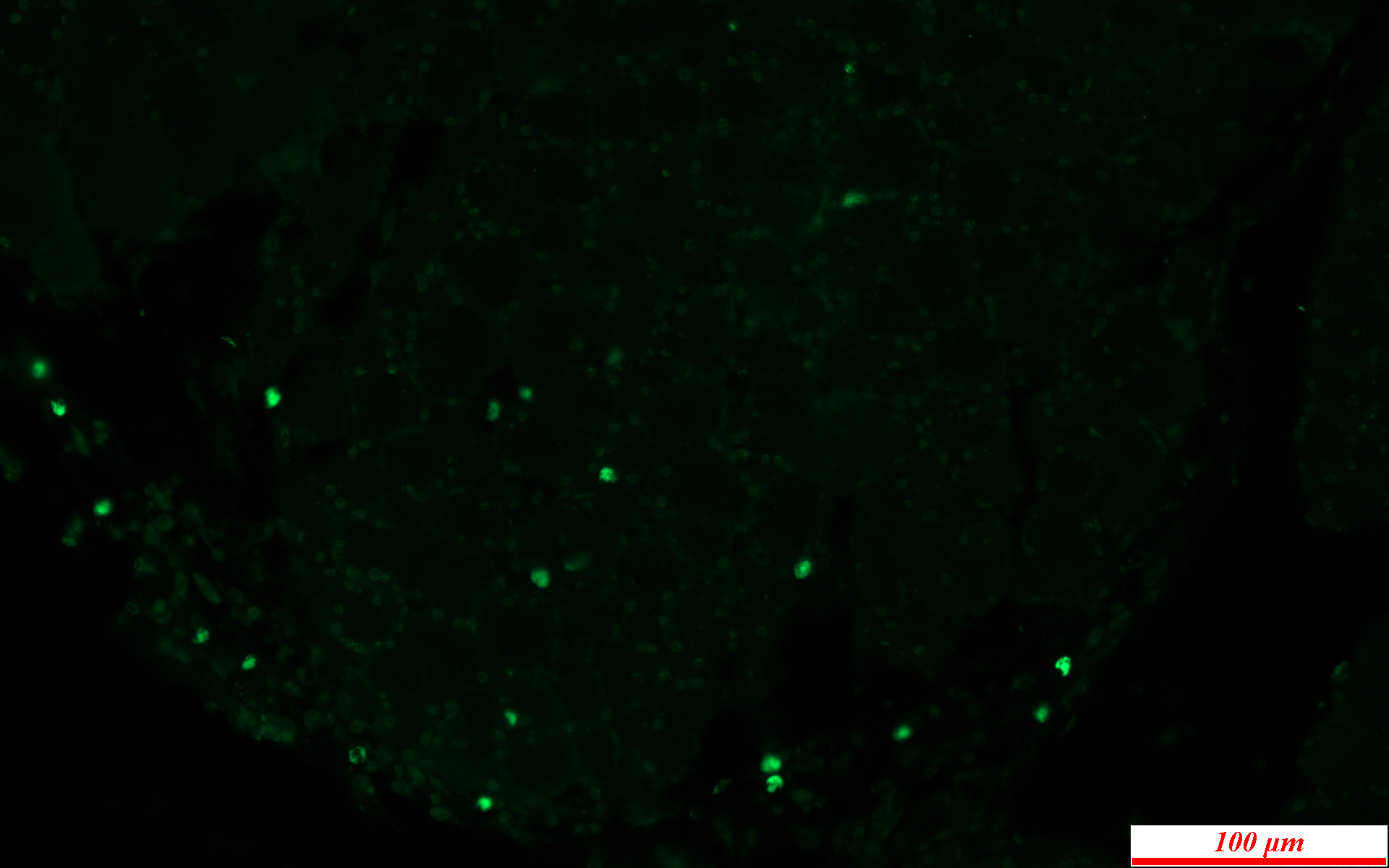

Supplement: Supplementary file 4 [file DataSheet_5.zip › Fig.11microscopy images of AP24h/AP24h Tunel.tif]

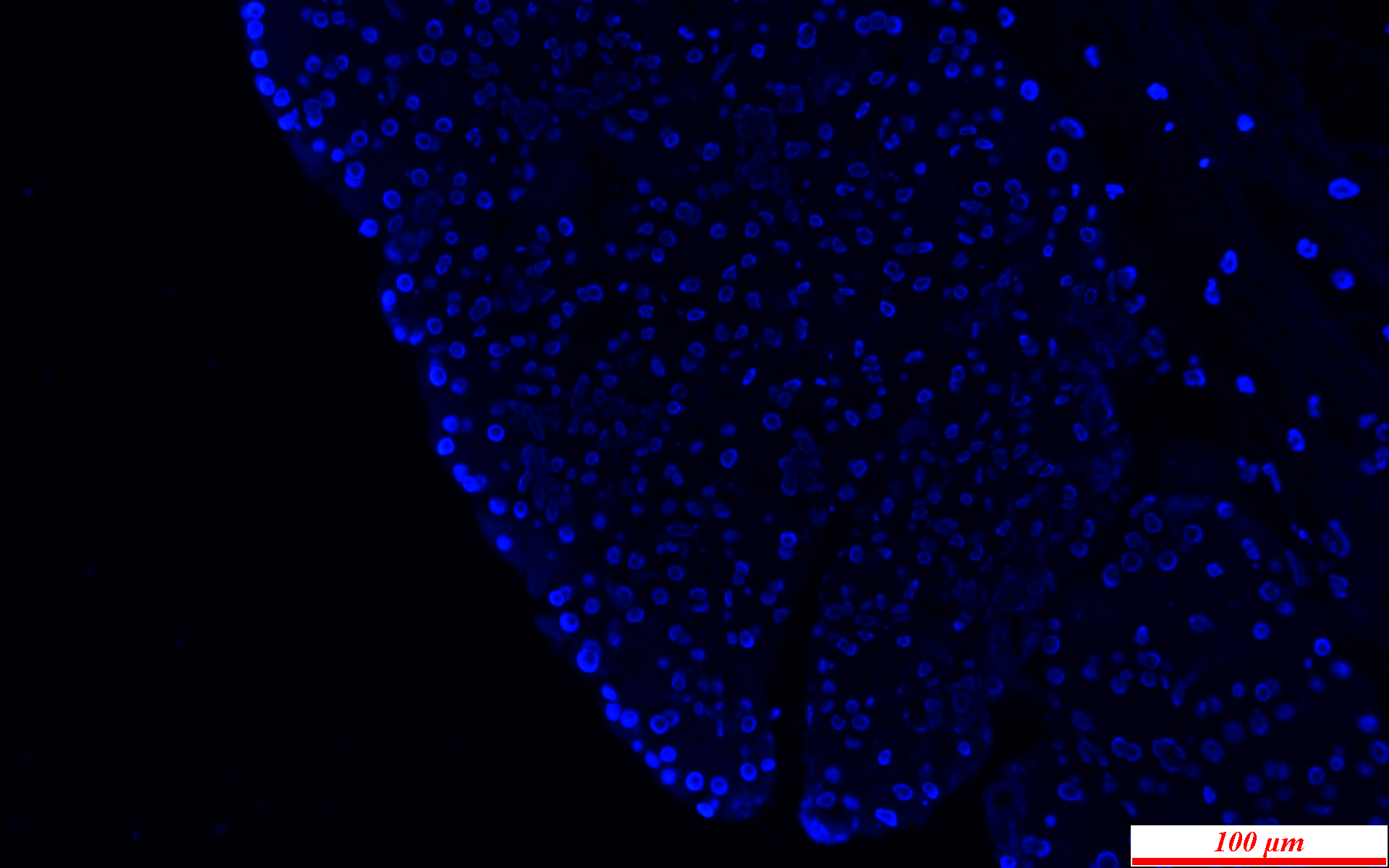

Supplement: Supplementary file 5 [file DataSheet_6.zip › Fig.11microscopy images of CH12h/CH12h DAPI.tif]

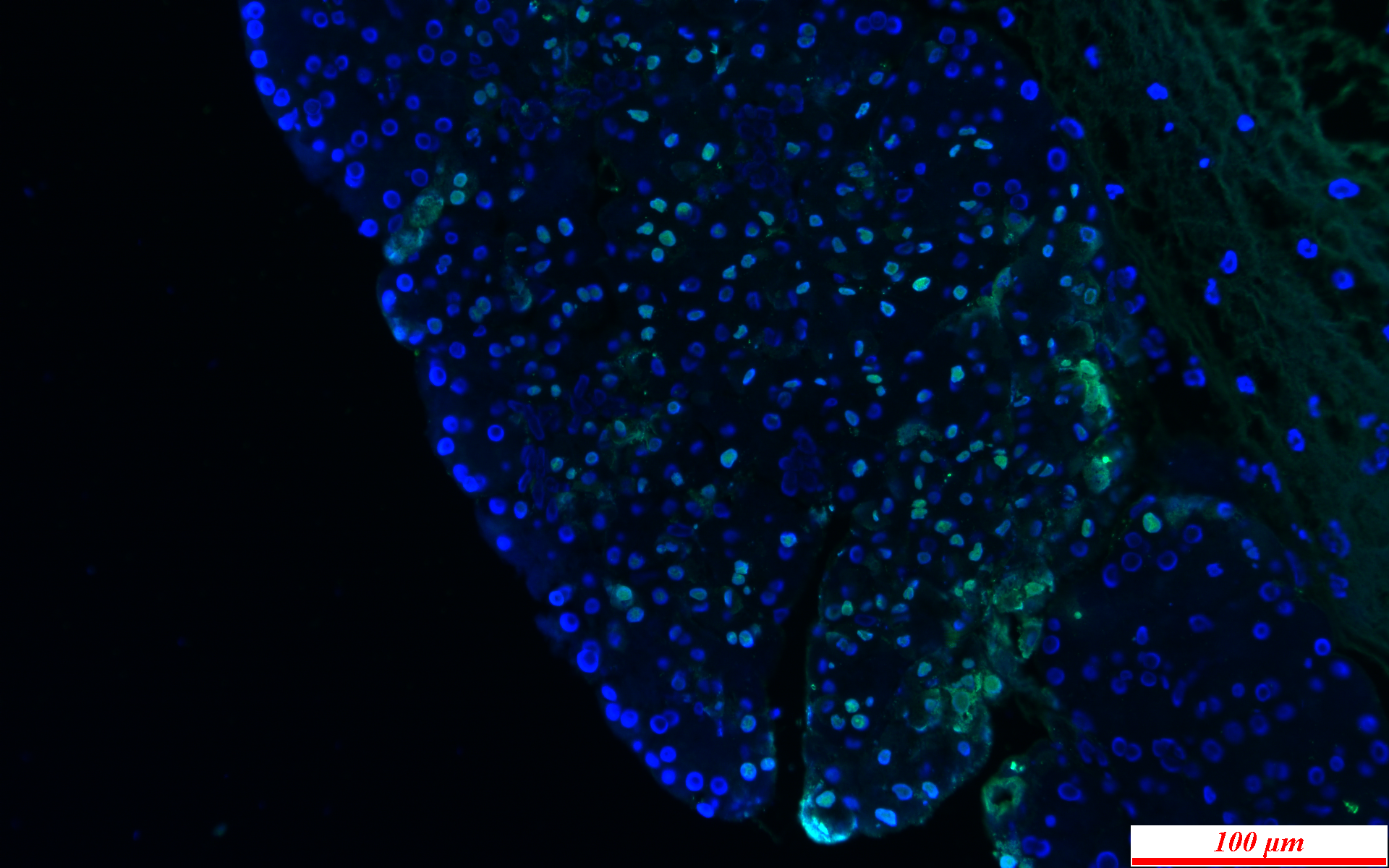

Supplement: Supplementary file 5 [file DataSheet_6.zip › Fig.11microscopy images of CH12h/CH12h Merge.tif]

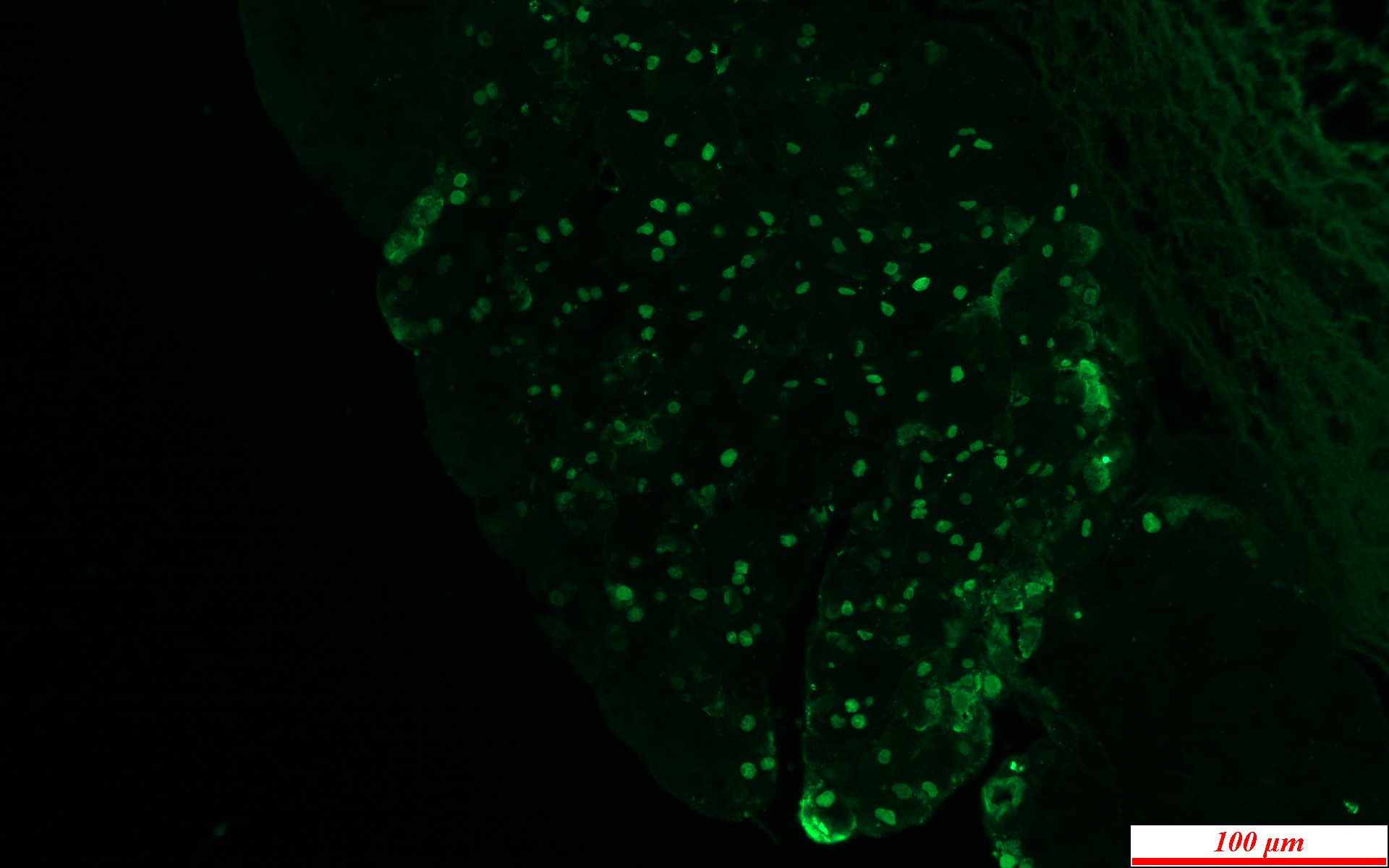

Supplement: Supplementary file 5 [file DataSheet_6.zip › Fig.11microscopy images of CH12h/CH12h Tunel.tif]

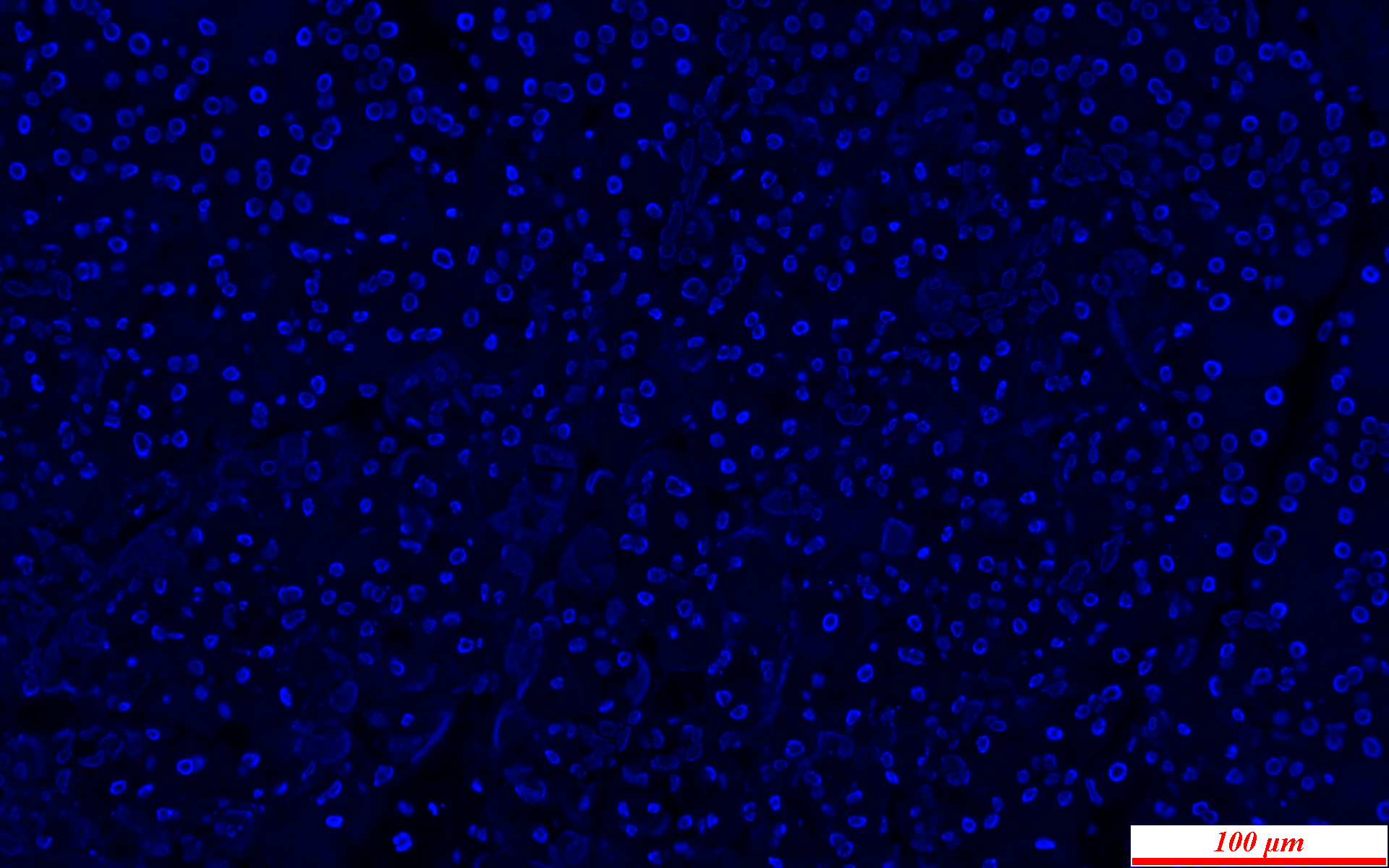

Supplement: Supplementary file 6 [file DataSheet_7.zip › Fig.11microscopy images of CH24h/ch24h DAPI.tif]

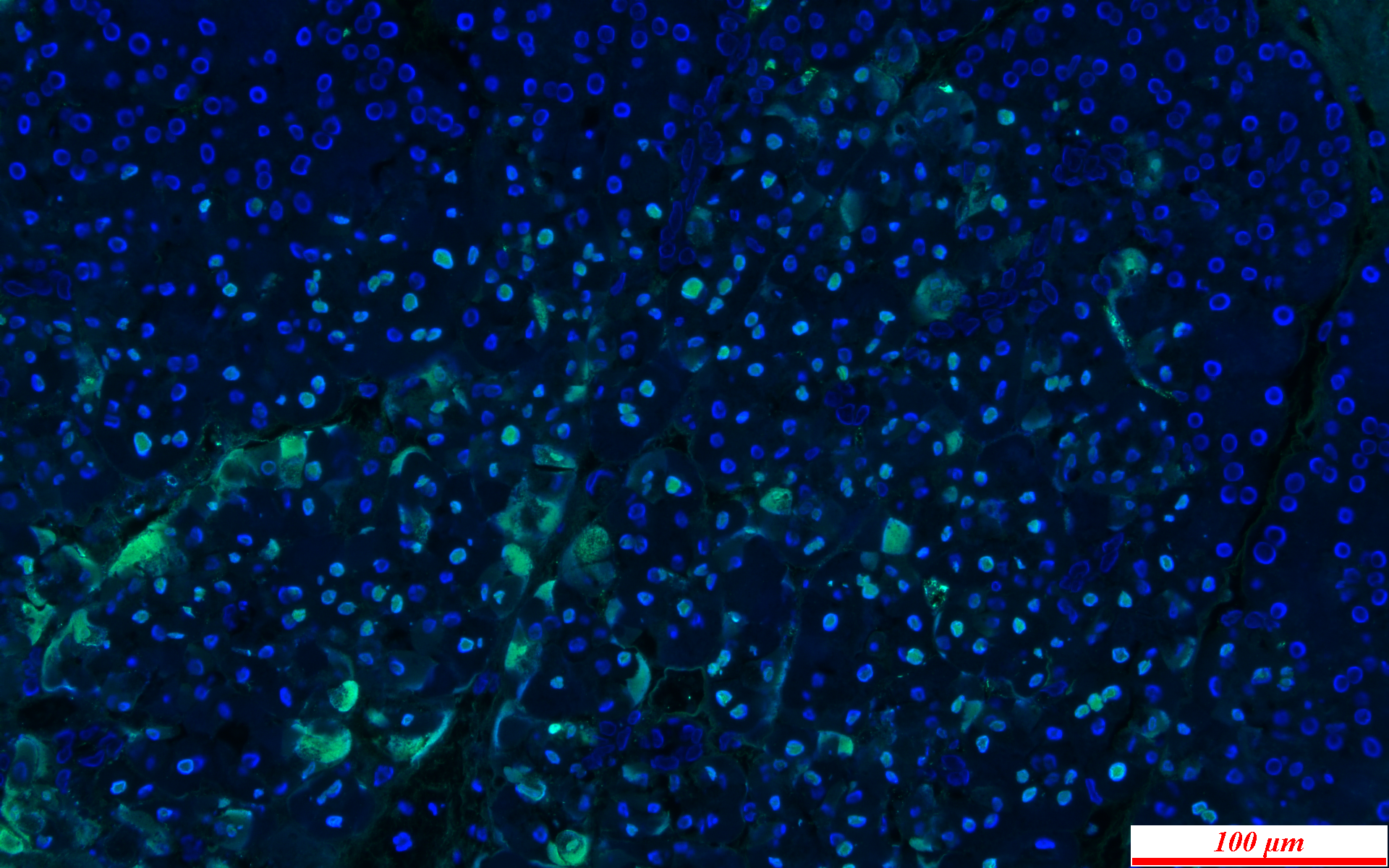

Supplement: Supplementary file 6 [file DataSheet_7.zip › Fig.11microscopy images of CH24h/ch24h Merge.tif]

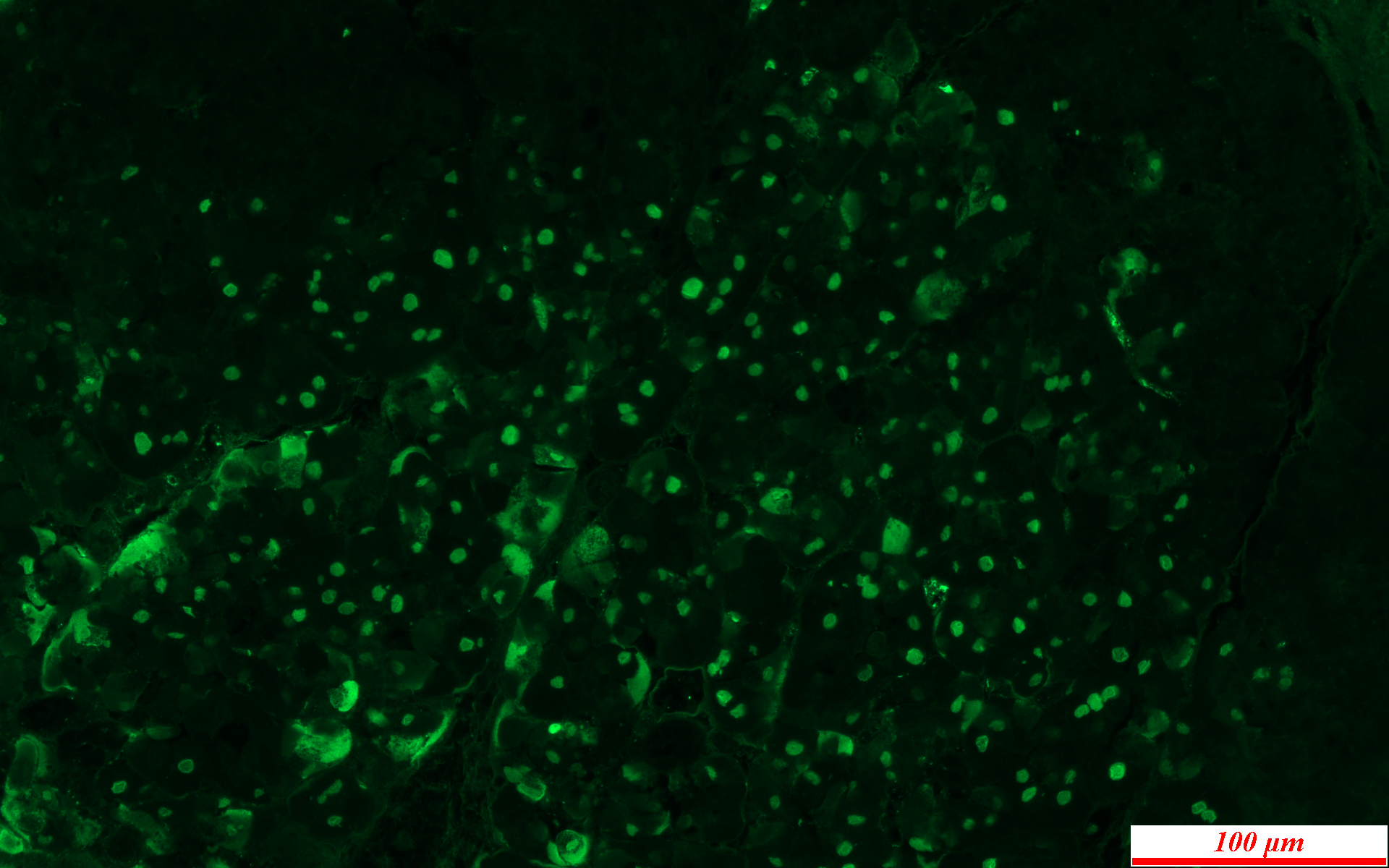

Supplement: Supplementary file 6 [file DataSheet_7.zip › Fig.11microscopy images of CH24h/ch24h Tunel.tif]

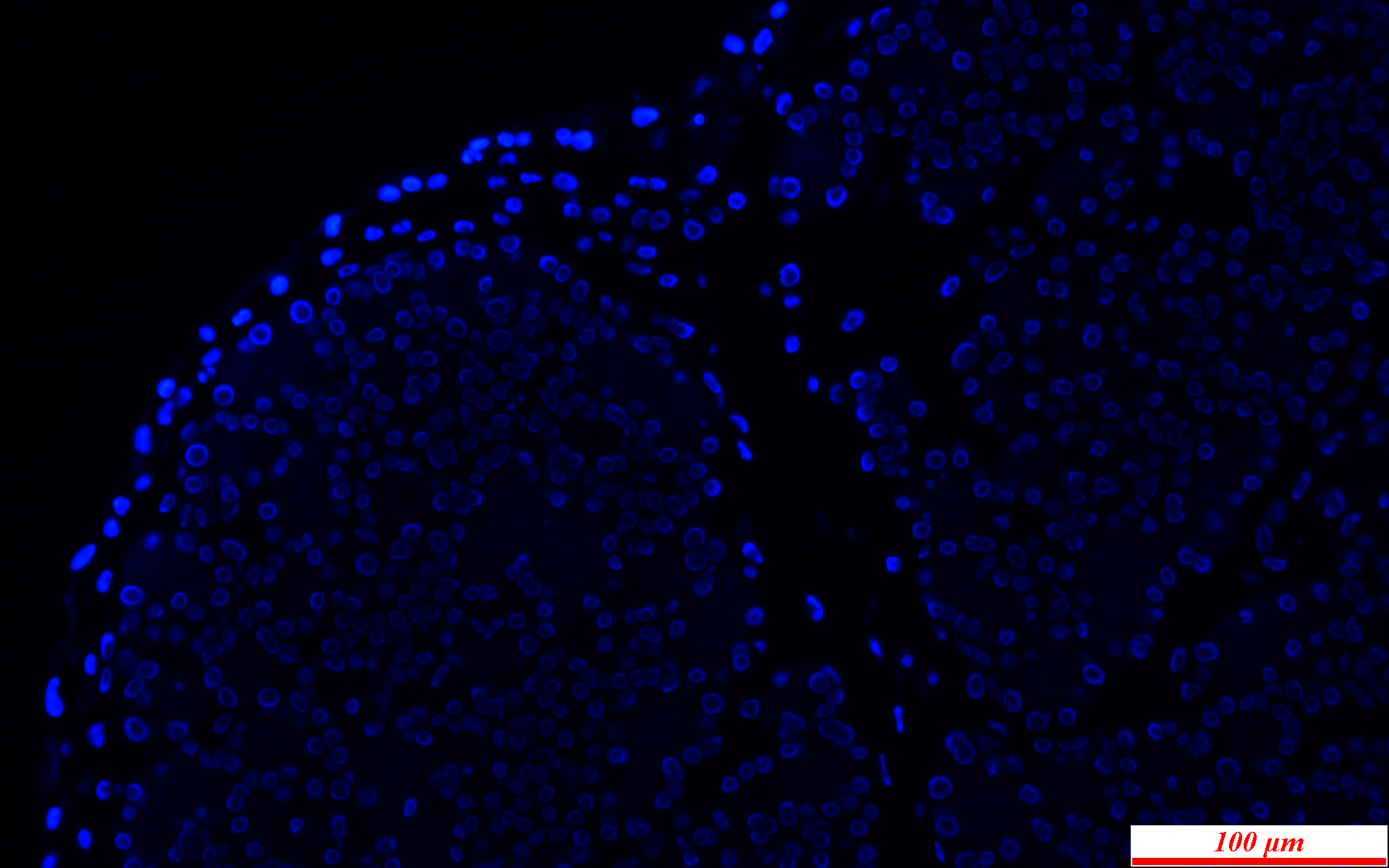

Supplement: Supplementary file 7 [file DataSheet_8.zip › Fig.11microscopy images of SHAM12h/sham12h DAPI.tif]

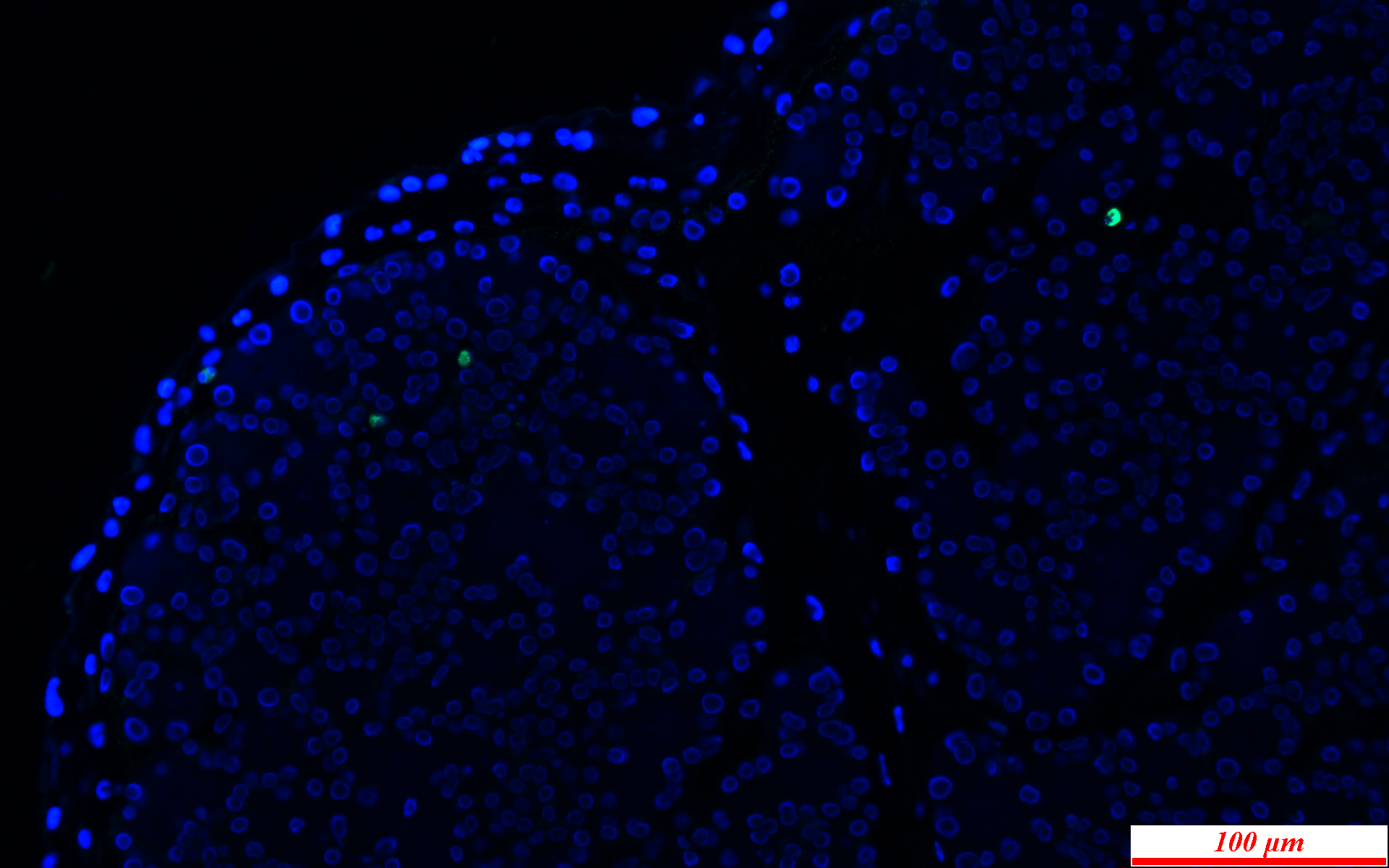

Supplement: Supplementary file 7 [file DataSheet_8.zip › Fig.11microscopy images of SHAM12h/sham12h Merge.tif]

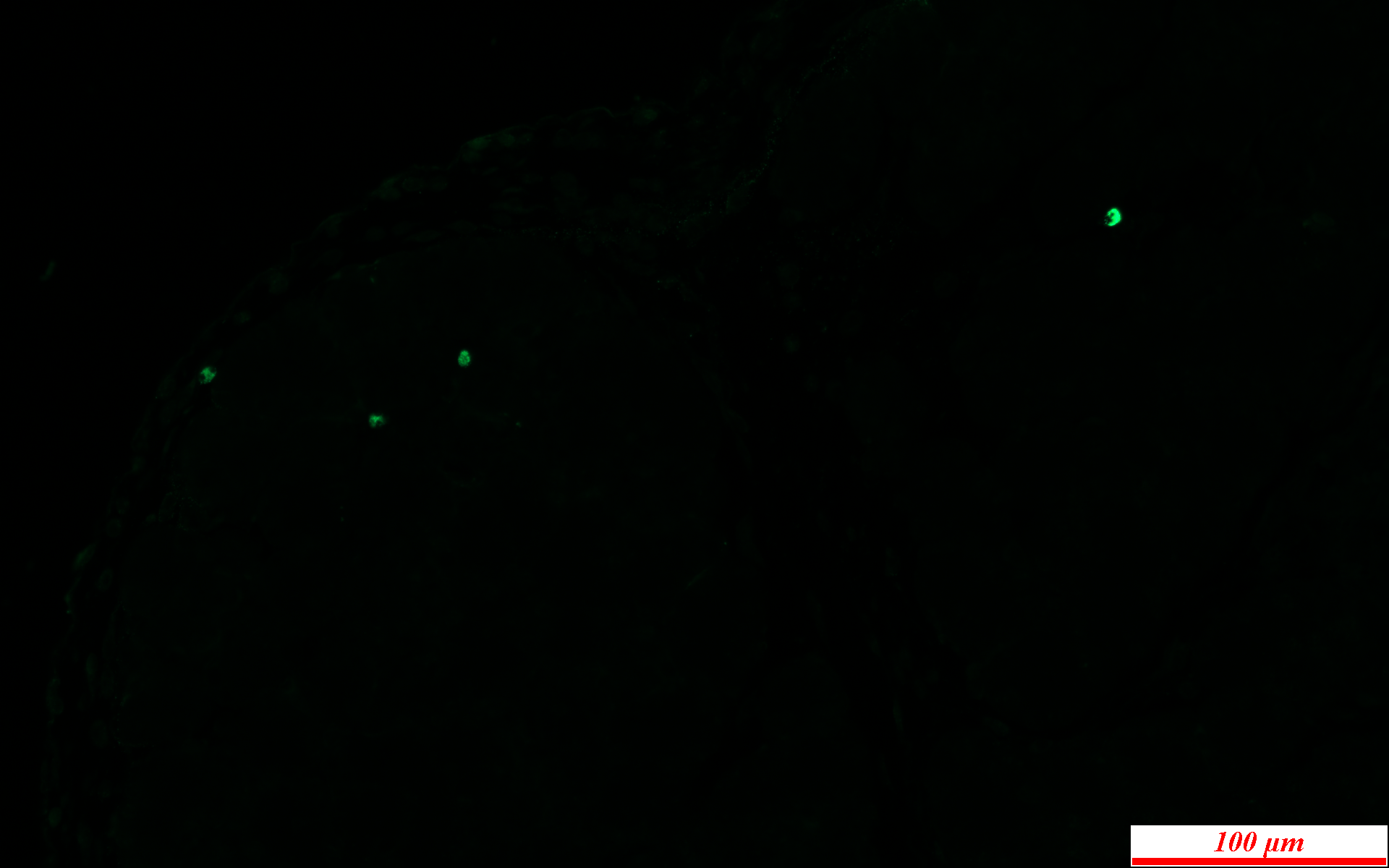

Supplement: Supplementary file 7 [file DataSheet_8.zip › Fig.11microscopy images of SHAM12h/sham12h Tunel.tif]

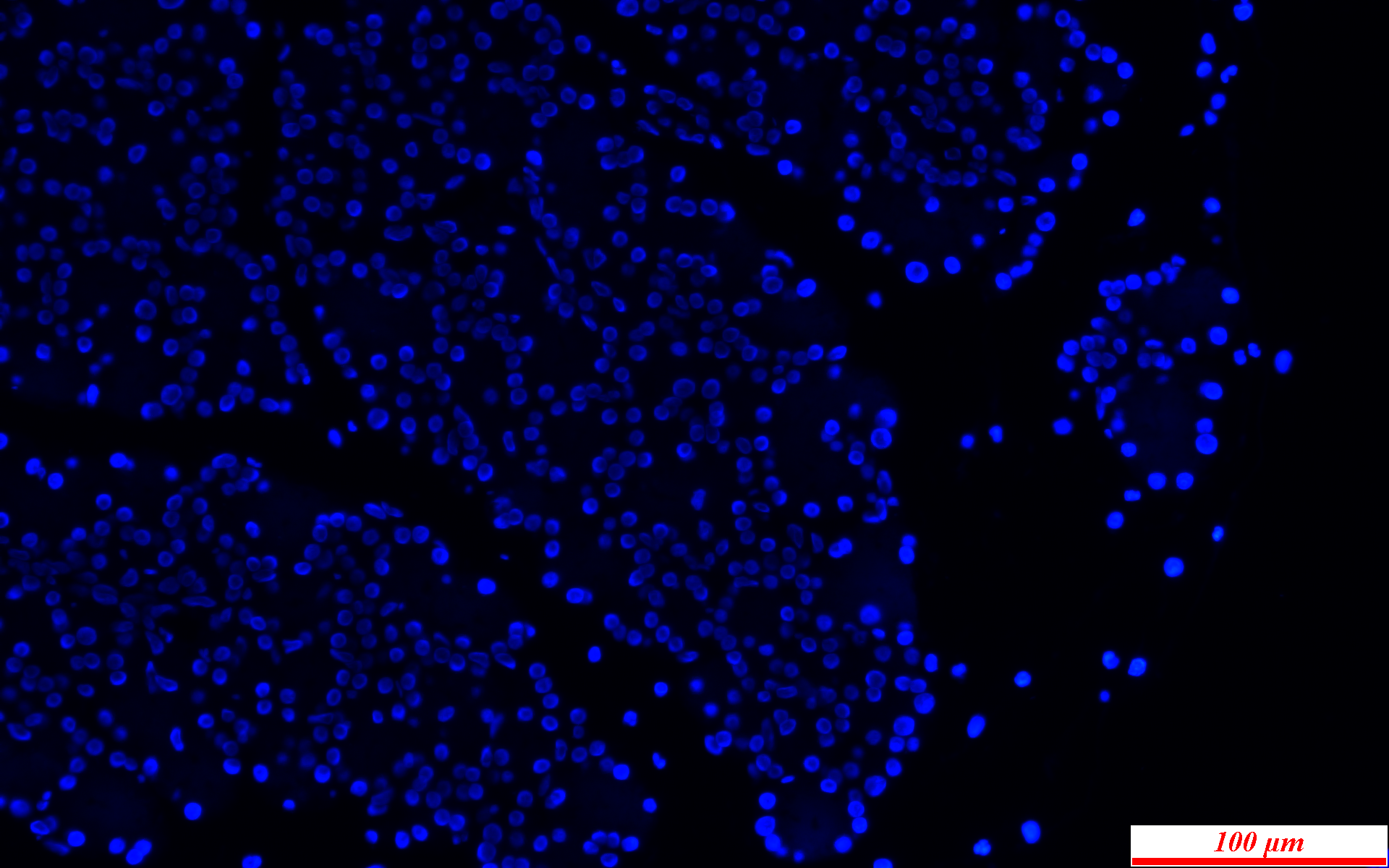

Supplement: Supplementary file 8 [file DataSheet_9.zip › Fig.11microscopy images of SHAM24h/sham24h DAPI.tif]

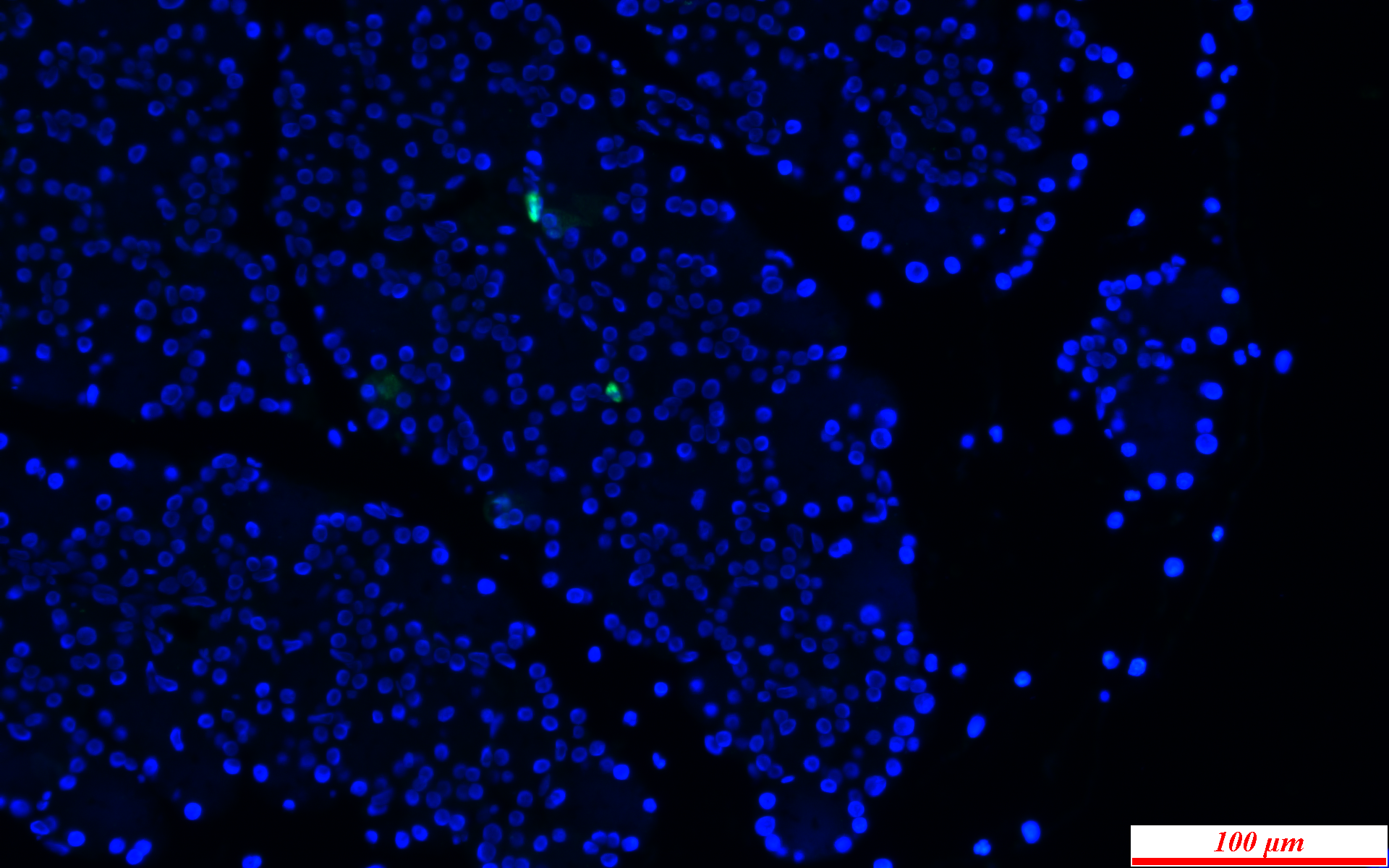

Supplement: Supplementary file 8 [file DataSheet_9.zip › Fig.11microscopy images of SHAM24h/sham24h Merge.tif]

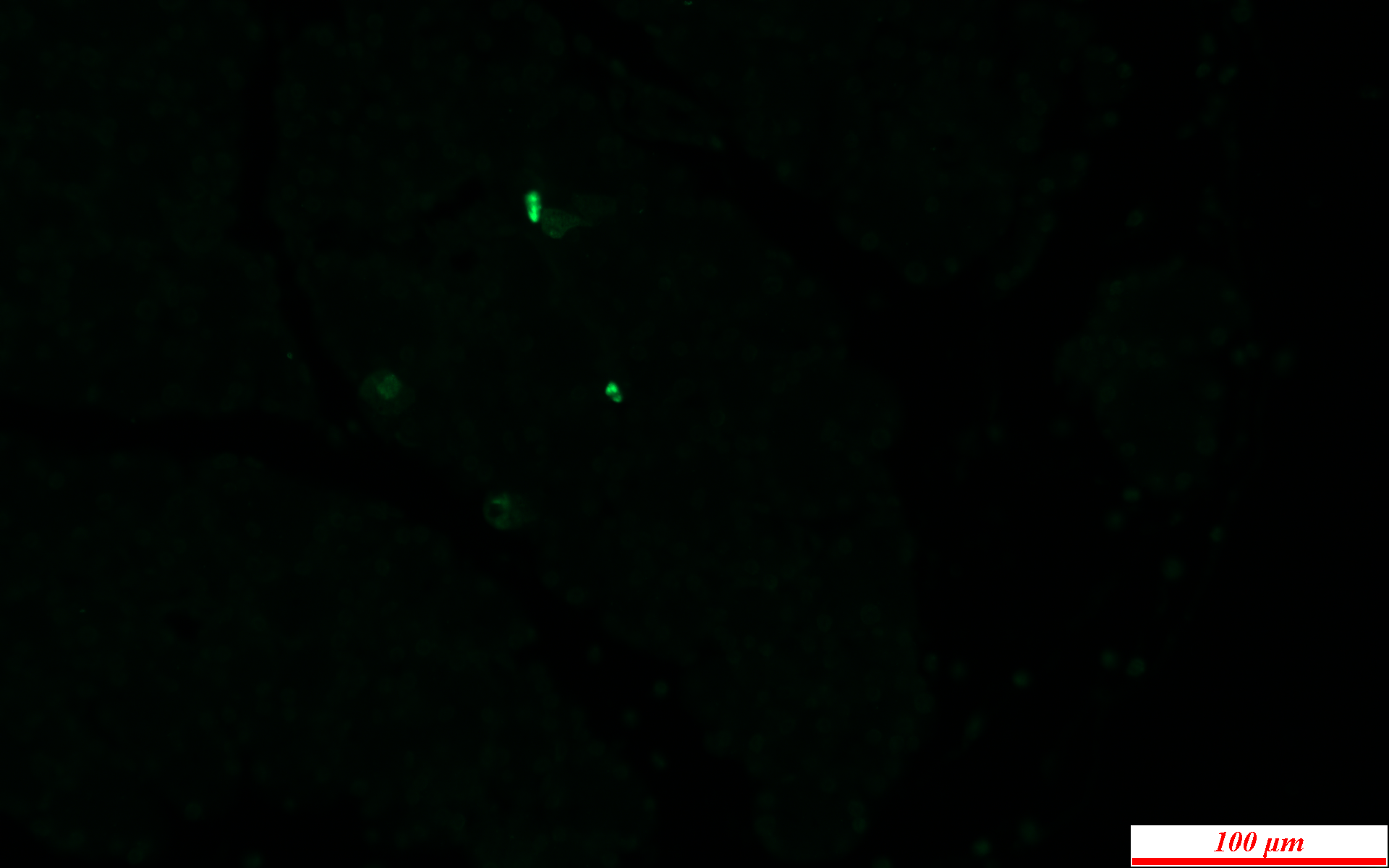

Supplement: Supplementary file 8 [file DataSheet_9.zip › Fig.11microscopy images of SHAM24h/sham24h Tunel.tif]

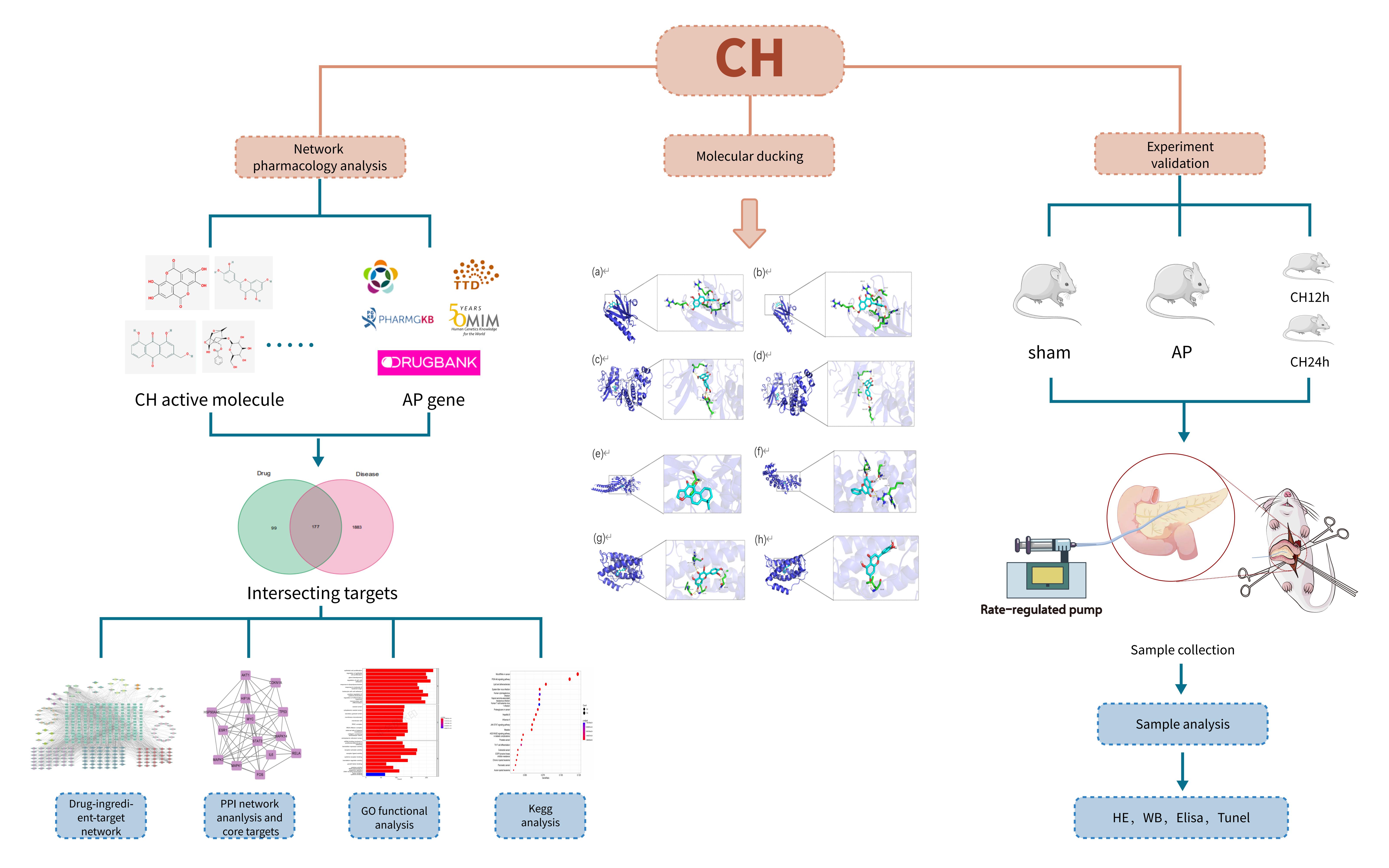

Supplement: Supplementary file 9 [file DataSheet_10.zip › Figures(without raw image)/Fig.1..jpg]

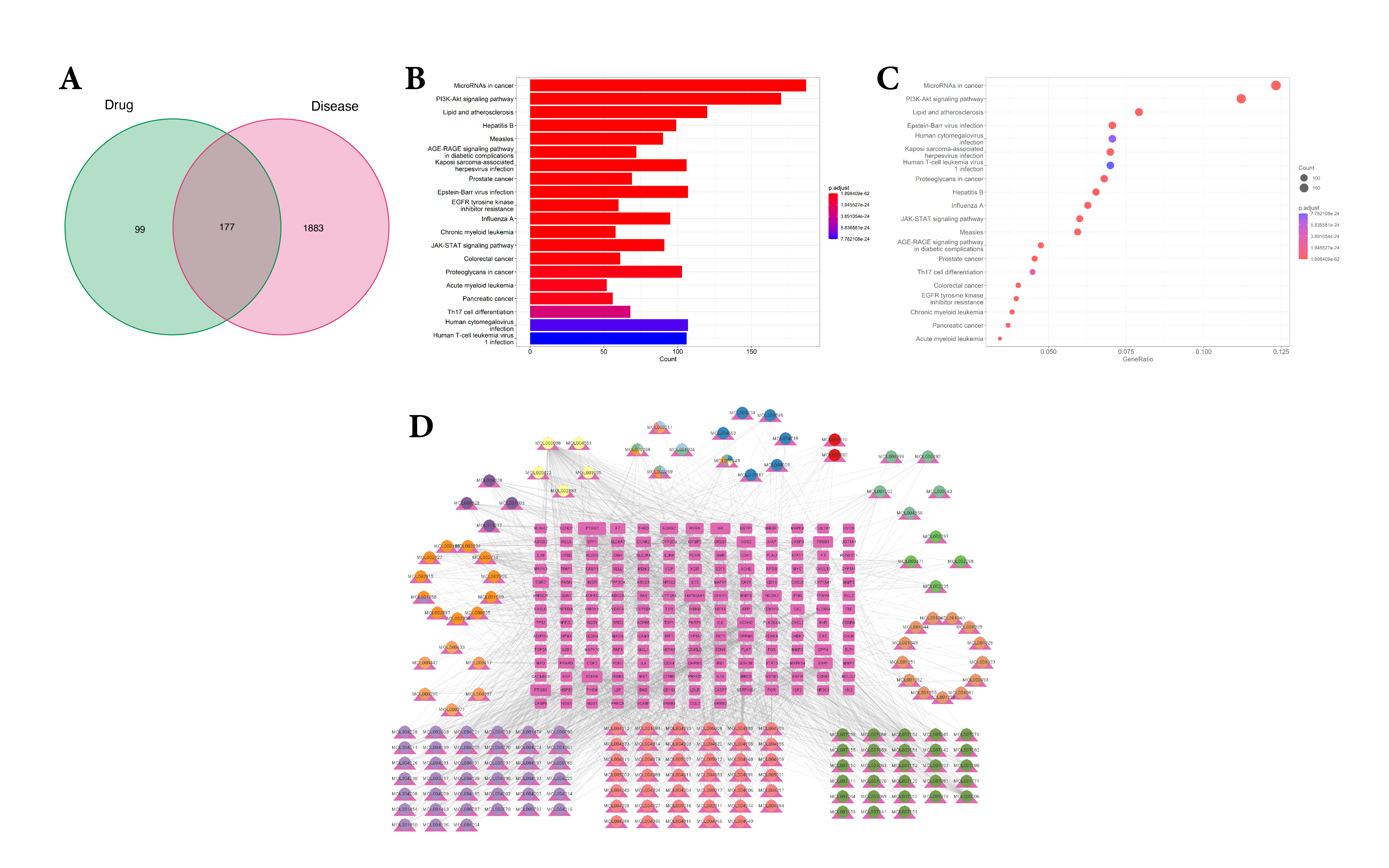

Supplement: Supplementary file 9 [file DataSheet_10.zip › Figures(without raw image)/Fig.2..jpg]

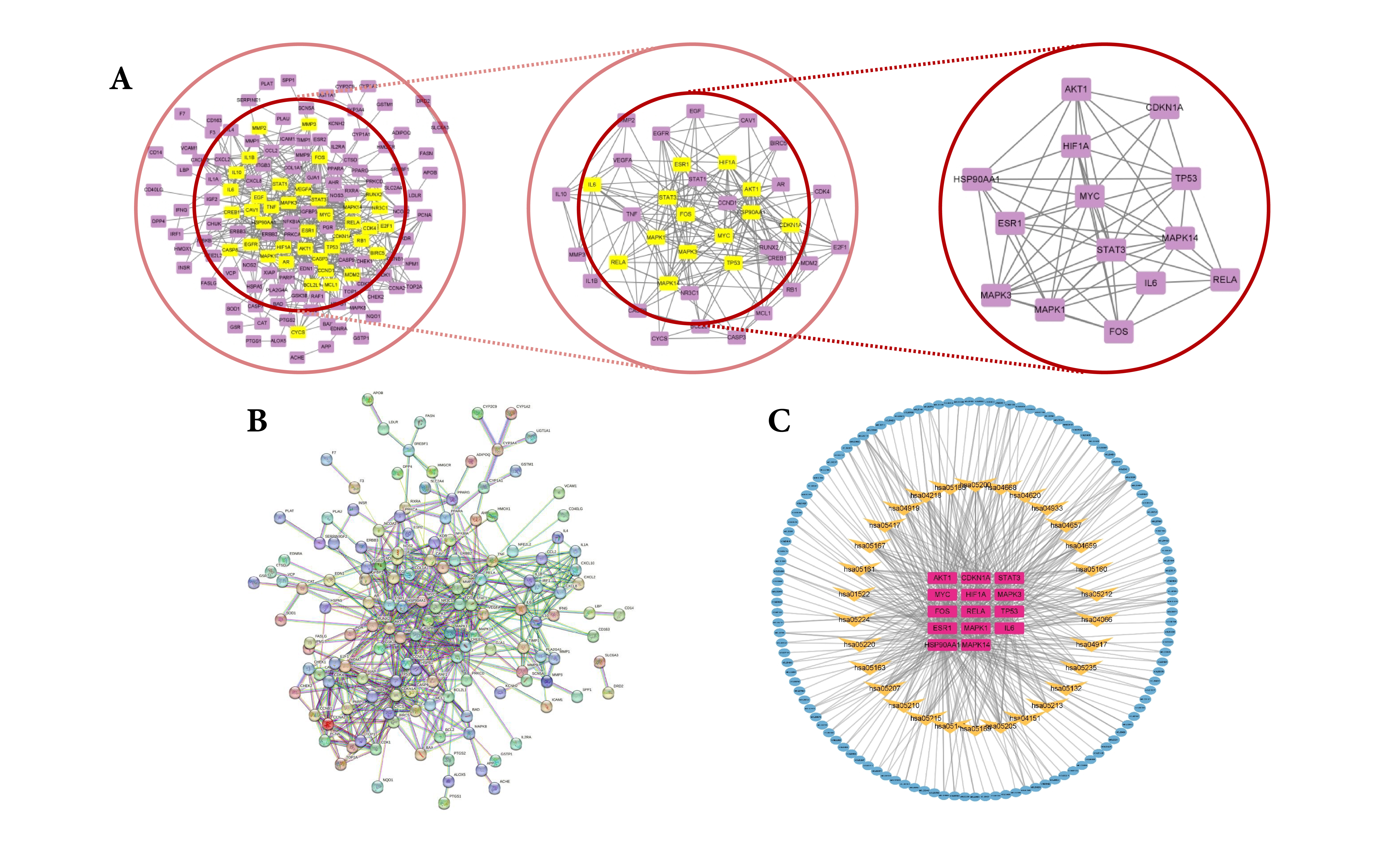

Supplement: Supplementary file 9 [file DataSheet_10.zip › Figures(without raw image)/Fig.3..jpg]
